# Long-term live imaging and multiscale analysis identify heterogeneity and core principles of epithelial organoid morphogenesis

**DOI:** 10.1186/s12915-021-00958-w

**Published:** 2021-02-24

**Authors:** Lotta Hof, Till Moreth, Michael Koch, Tim Liebisch, Marina Kurtz, Julia Tarnick, Susanna M. Lissek, Monique M. A. Verstegen, Luc J. W. van der Laan, Meritxell Huch, Franziska Matthäus, Ernst H. K. Stelzer, Francesco Pampaloni

**Affiliations:** 1 Physical Biology Group, Buchmann Institute for Molecular Life Sciences (BMLS), Goethe-Universität Frankfurt am Main, Frankfurt am Main, Germany; 2 Frankfurt Institute for Advanced Studies and Faculty of Biological Sciences, Goethe-Universität Frankfurt am Main, Frankfurt am Main, Germany; 3 Department of Physics, Goethe Universität Frankfurt am Main, Frankfurt am Main, Germany; 4 Deanery of Biomedical Science, University of Edinburgh, Edinburgh, UK; 5 Experimental Medicine and Therapy Research, University of Regensburg, Regensburg, Germany; 6 Department of Surgery, Erasmus MC – University Medical Center, Rotterdam, The Netherlands; 7 The Wellcome Trust/CRUK Gurdon Institute, University of Cambridge, Cambridge, UK; 8 Present address: Max Planck Institute of Molecular Cell Biology and Genetics, Dresden, Germany

## Abstract

**Background:**

Organoids are morphologically heterogeneous three-dimensional cell culture systems and serve as an ideal model for understanding the principles of collective cell behaviour in mammalian organs during development, homeostasis, regeneration, and pathogenesis. To investigate the underlying cell organisation principles of organoids, we imaged hundreds of pancreas and cholangiocarcinoma organoids in parallel using light sheet and bright-field microscopy for up to 7 days.

**Results:**

We quantified organoid behaviour at single-cell (microscale), individual-organoid (mesoscale), and entire-culture (macroscale) levels. At single-cell resolution, we monitored formation, monolayer polarisation, and degeneration and identified diverse behaviours, including lumen expansion and decline (size oscillation), migration, rotation, and multi-organoid fusion. Detailed individual organoid quantifications lead to a mechanical 3D agent-based model. A derived scaling law and simulations support the hypotheses that size oscillations depend on organoid properties and cell division dynamics, which is confirmed by bright-field microscopy analysis of entire cultures.

**Conclusion:**

Our multiscale analysis provides a systematic picture of the diversity of cell organisation in organoids by identifying and quantifying the core regulatory principles of organoid morphogenesis.

**Supplementary Information:**

The online version contains supplementary material available at 10.1186/s12915-021-00958-w.

## Background

Understanding the principles of collective cell behaviour in mammalian organs during development, homeostasis, regeneration, and pathogenesis requires simplified models mimicking the in vivo cell-cell and cell-matrix interactions. To this aim, organoids provide an ideal in vitro model. Organoids are three-dimensional (3D) cultures obtained from pluripotent stem cells (embryonic or induced pluripotent stem cells) or organ-derived adult stem cells [1]. Organoid systems recapitulating the brain and the majority of epithelial organs have been established. These systems reproduce aspects of organ-specific development and disease [2, 3] and are valuable for personalised [4, 5] and regenerative medicine [6]. Multicellular self-organisation determines organoid behaviour and morphology. For instance, epithelial organoids can acquire a spherical (“monocystic” (Additional file 1: Definitions), budding (“branched”), but also a dense (“polycystic” (Additional file 1: Definitions) phenotype [7, 8]. Organoids are therefore a valid model to understand the principles of tissue self-organisation at the mesoscale, which are largely unknown [9].

In order to fill this knowledge gap, a quantitative analysis at single-cell resolution is essential. A multiscale approach is required, capturing the cell-to-cell variability while monitoring the entire organoid system [10] (Additional file 1: Definitions).

The advancement of molecular biology allows quantifications of large-scale omics data at single-cell resolution. For example, high-throughput single-cell transcriptomics detect rare cell populations and trajectories between distinct cell lineages [11]. Unlike most single-cell molecular characterisations, time-resolved advanced microscopy enables both spatio-temporal analysis of the organoids’ global morphology and “zooming-in” on the fates of a single cell. In previous studies, Bolhaquiero et al. [12] were able to combine single-cell molecular and image-based analyses and proved chromosome segregation errors with up to 18-h-long image acquisitions in a confocal microscope. In an approach using an inverted light sheet microscope, Serra et al. [8] were able to perform 5-day long live acquisitions of individual organoids.

Ultimately, the experimental quantitative data on organoid dynamics should serve as a foundation for mathematical models, which predict the experimental outcome and test hypotheses about underlying mechanisms of observed behaviours by altering controllable parameters in silico [13]. In our study, we focus on two types of organoids one from mouse pancreas and one from human liver tumour as representatives for a spherical as well as a polycystic phenotype. Murine pancreas-derived organoids (mPOs) are used as a model to study pancreas development and the regeneration of pancreatic β cells [14]. Human cholangiocarcinoma-derived organoids (hCCAOs) are promising models to study personalised treatment of primary liver cancer [4]. As organoids retain the genetic and epigenetic characteristics of the donor they were derived from, understanding intra- and inter-organoid heterogeneity is a major challenge in the field. Broutier et al. [4] showed that hCCAOs retain the expression profile, the genetic mutations, and the histological features of the individual patients from which they were obtained. The morphology of these organoids are particularly heterogeneous, including the regular hollow sphere of healthy liver organoids, the more compact structure or the irregular hollow cysts displayed by hCCAOs. Li et al. [15] tested 129 chemotherapeutic compounds on 27 patient-derived liver cancer organoid lines, including hCCAOs. The organoids showed a high inter-patient functional heterogeneity concerning the response to drug treatment. Strikingly, the same heterogeneity was also found in organoid lines obtained from different regions of the same cancer resection, pointing intra-patient variability. As most of the research efforts are focused onto human organoids, the heterogeneity of murine pancreatic organoids has been poorly investigated. In fact, most of the evidence derives from our own study conducted in the LSFM4LIFE consortium and are presented in this work. So far, the organoid morphology has been analysed qualitatively by immuno-fluorescent marker localisation and bright-field observations at single time points. Here, in order to assess cellular dynamics in organoid cultures and identify their morphological organisational principles, we developed two complementary image-based analysis pipelines covering multiple scales: (1) A “light sheet pipeline”, based on light sheet-based fluorescence microscopy (LSFM), addressing the micro- (single cell) and the mesoscale (individual organoid). (2) A “bright-field pipeline”, based on bright-field microscopy, accessing both the meso- and macroscale (entire organoid culture) (Additional file 2: Fig. S1).

The light sheet pipeline relies on image acquisition with the Lightsheet Z.1 (Zeiss) microscope, for which we further developed a custom FEP-foil cuvette [16] (*further referred to as Z1-FEP cuvette*, Additional file 1: Definitions) as a sample holder. Based on tagging cells with fluorescent fusion proteins, this setup allowed for observations of cellular dynamics in live epithelial organoid cultures for up to 7 days while retaining optimal physiological conditions (3D ECM environment, precisely defined medium, constant pH, and controlled temperature). Primary cell cultures, such as organoids, require minimal exposure to phototoxic effects, which is given by low-energy exposure, due to a fast image acquisition as well as *z*-plane-confined illumination in LSFM [17, 18]. The confined illumination also yields a higher axial resolution compared to other microscopy systems, while still facilitating the acquisition of large samples [19, 20]. These features enable us to monitor large numbers of organoids simultaneously (approx. 100–200 organoids, depending on seeding density) in a maximum volume of about 8 mm^3^. The high image quality allows for detailed visualisation of highly dynamic processes using various image analysis programmes. In a qualitative approach, we also demonstrate that the image quality is promising regarding the tracking of cell movements using commercially available software. Furthermore, the data acquired by LSFM allow extraction and quantification of several organoid features, including cell number and organoid volume at the micro- and mesoscale via our validated nuclei segmentation [21, 22]. Based on the quantitative data obtained from the light sheet pipeline (cell count and cell division rates), we developed a 3D agent-based model (Additional file 1: Definitions) to investigate the underlying mechanics driving single-organoid behaviour. Such models provide a technique to represent a wet-lab experiment under idealised conditions [23]. In contrast to the basic mechanisms proposed by the model of Ruiz-Herrero et al. [24], which describes the dimensionless radius for hydraulically gated oscillations in spherical systems, we built a full elastic 3D model, based on the core principles formulated in Stichel et al. [25]. We expect organoids to have a similar morphogenic behaviour to other 3D-cell cultures, such as MDCK cysts and epithelial tissues, namely, they grow by mitosis, display an apical-basal polarisation [26], and secrete osmotically active substances into their lumen [27]. Further, we assume that neighbouring cells are tightly connected via cell-cell junctions [28] and the cell layer ruptures if the internal pressure reaches a critical point.

The single-cell resolution achieved by the light sheet pipeline is necessary for studying single-cell dynamics and collective cell dynamics in individual organoids in depth. However, the large amounts of data acquired by this pipeline require considerable computational resources, which hinder the extraction and quantification of macroscale (entire organoid culture) features. We therefore developed the bright-field pipeline that measures luminal size changes at individual-organoid resolution based on projected luminal areas (Additional file 1: Definitions). This pipeline enables the observation of entire organoid cultures (approx. 100–200 organoids within 25 μl ECM droplets, depending on seeding density) over several days while retaining optimal physiological conditions. In addition, the bright-field setup allows label-free image acquisition, which ensures minimal exposure to phototoxic effects. Quantification of the projected luminal areas over time yields features on a mesoscale level, such as minimal and maximal area of individual organoids, which are used to determine the median area increase of the entire culture at the macroscale level.

Our light sheet data indicate that epithelial organoids show size oscillations (expansion and decline phases) (Additional file 1: Definitions), which are frequently observed in small organoids (diameter < 400 μm), but much less in large organoids (diameter > 400 μm). This is reflected in our 3D agent-based model, which indicates the size oscillations arise in response to an interplay of an increase of the internal pressure, the cell division dynamics, and the mechanical properties of the single cells. The critical internal pressure due to release of osmotically active substances into the lumen is reached earlier in organoids with increased surface-to-volume ratios (small organoids) compared to organoids with reduced surface-to-volume ratios (large organoids). We further verified these findings by quantifying the size oscillations in entire organoid cultures using the bright-field pipeline.

In summary, our approach reveals the dynamics of organoid cultures from single-cell and single-organoid scale to the complete culture scale, ascertaining the core regulatory principles (Additional file 1: Definitions) of their multicellular behaviour.

## Results

### Long-term live imaging with LSFM allows detailed visualisation of dynamic processes in organoid morphogenesis and reveals high heterogeneity in single-cell and individual-organoid behaviour

To gain deeper insights into the dynamic cellular processes occurring within organoid systems, we developed Z1-FEP cuvette holders for live imaging with the Zeiss Lightsheet Z.1 microscope system (Additional file 3: Fig. S2). As previously described [16], ultra-thin FEP-foil cuvettes are sample holders for LSFM which preserve physiological culture conditions for organoid cultures and allow the acquisition of high-resolution images at a single-cell level. Using the Z1-FEP cuvette, we recorded the formation and development of hCCAOs expressing H2B-eGFP (nuclei marker) and LifeAct-mCherry (F-actin cytoskeleton marker) and mPOs expressing Rosa26-nTnG (nuclei marker) for up to 7 days. The medium was exchanged every 48 h to ensure sufficient nutrient supply. Temperature and CO_2_ levels were controlled to ensure optimal growth conditions (Additional file 4: Fig. S3, Additional file 5: Fig. S4). The setup enabled us to monitor dynamic processes at high temporal and spatial resolution in up to 120 organoids simultaneously contained in one Z1-FEP cuvette (in this example in a total volume of 5.2 mm^3^ of technically possible 8 mm^3^) (Additional file 6: Fig. S5a, Additional file 7: Fig. S6; Additional file 15: Video 1). The images acquired by LSFM allow for detailed qualitative inspections and detailed feature tracking of several dynamic cellular processes at single-cell resolution (Additional file 16: Video 2).

**Fig. 1:**
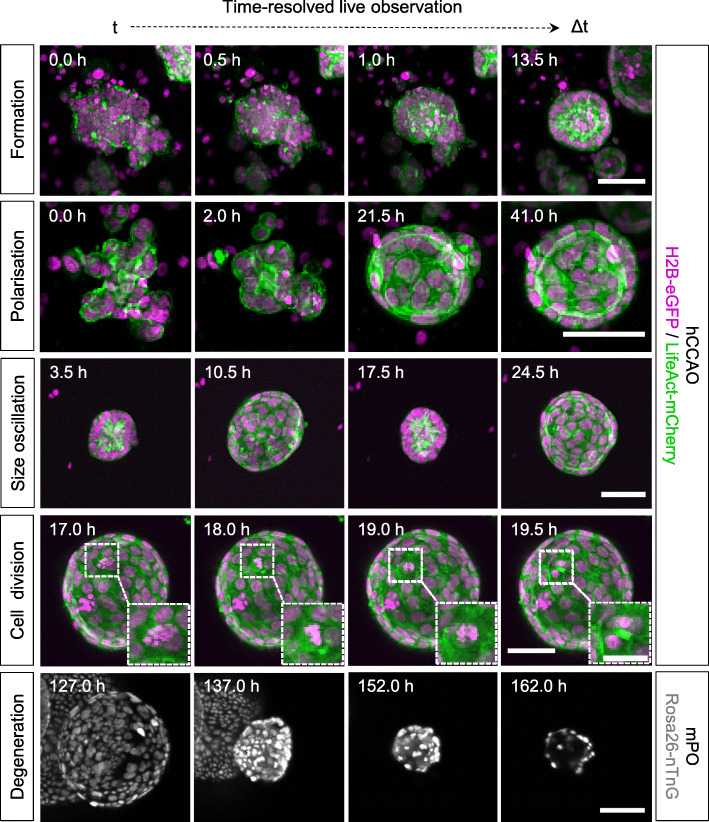
Time-resolved live LSFM recordings for detailed qualitative inspections of dynamic morphological processes in organoid development. hCCAOs and mPOs were seeded into Z1-FEP-cuvettes for long-term live observations. They expressed the nuclei marker H2B-eGFP (magenta) or Rosa26-nTnG (grey) and the F-actin cytoskeletal marker LifeAct-mCherry (green). About 120 organoids were recorded in image stacks up to 900 *z*-planes deep for at most 7 days. The figure shows excerpts of maximum intensity *z*-projections. Microscope: Zeiss Lightsheet Z.1; objective lenses: detection: W Plan-Apochromat × 20/1.0, illumination: Zeiss LSFM × 10/0.2; laser lines: 488 nm, 561 nm; filters: laser block filter (LBF) 405/488/561; voxel size: 1.02 × 1.02 × 2.00 μm^3^; recording interval: 30 min; scale bars: 50 μm, 25 μm (inset)


Visual inspection of the acquired data revealed that the initially seeded organoid cell clusters contract before the cells within the clusters start to rearrange and form spherical structures (Fig. 1, Formation). The cells within these spherical structures begin to polarise and form a lumen (in this example around 13.5 h), indicated by a stronger F-actin signal at the apical (luminal) side of the cell membranes. Potentially dead cells accumulate within the lumen, indicated by loss of the LifeAct-mCherry signal and by smaller nuclei with stronger H2B-eGFP signals, hinting towards apoptotic nuclear condensation [29]. The polarisation of cells in the epithelial monolayer is maintained during luminal expansion and is still clearly visible at later stages of organoid development (in this example around 41.0 h) (Fig. 1, Polarisation). We also observed size oscillations (Additional file 1: Definitions) during luminal expansion, where the organoid inflates and then suddenly collapses to about its initial size before it starts to expand again (Fig. 1, Size oscillation). The recording interval of 30 min also allows us to visually track single-cell division events (here: over a time course of 2.5 h) (Fig. 1, Cell division). We were also able to observe polarisation and cell division events in isolated single cells (Additional file 6: Fig. S5b), which remained dormant for relatively long periods during observation but eventually started to form organoids. We identified an overall shrinking of the organoid, nuclear condensation, and a fading nuclei signal to be hallmarks of organoid degeneration (Additional file 1: Definitions) (Fig. 1, Degeneration; Additional file 15: Video 1). This process is initiated upon extended culturing without further medium exchange (here: after about 100 h).

**Additional file 15: Video 1.** Time-resolved observations of epithelial organoids growing in Z1-FEP-cuvettes. hCCAOs expressing H2B-eGFP as nuclei marker (red) and LifeAct-mCherry as F-actin cytoskeletal marker (green) and mPOs expressing Rosa26-nTnG (grey) as nuclei marker were recorded over 10 h and 143 h respectively. The formation of organoids from the initially seeded cell clusters, including cell cluster contraction, cell polarisation, lumen formation and expansion can be followed. After about 100 h of observation some mPOs begin to display signs of degeneration due to extended culturing without further medium exchange. These signs of degeneration include an overall shrinking of the organoid, followed by nuclear condensation and fading of the nuclei signal. The temporal loss of signal during the acquisition happens due to evaporation and a subsequent constant loss of mounting medium in the imaging chamber. A manual refill re-established optimal acquisition conditions. The loss of the mounting medium does not alter the organoids within the cuvette since the cuvette separates the mounting medium from the organoid growth medium. Microscope: Zeiss Lightsheet Z.1; detection objective: W Plan-Apochromat 20x/1.0, illumination objective: Zeiss LSFM 10x/0.2; laser lines: 488 nm, 561 nm; filters: laser block filter (LBF) 405/488/561; voxel size: 1.02 × 1.02 × 2.00 μm^**3**^; recording interval: 30 min; scale bar: 50 μm. (MP4 10,289 kb)


**Additional file 16: Video 2.** Time-resolved 3D volume rendering of the formation process of an entire organoid culture observed within one Z1-FEP-cuvette. hCCAOs expressing H2B-eGFP as nuclei marker (red) and LifeAct-mCherry as F-actin cytoskeletal marker (green) were imaged for 5 days. The movie shows an excerpt of the first 10 h of the recorded data set. All organoids within the cuvette were segmented and tracked over these first 10 h of recording. The initial processes of cell cluster contraction, lumen formation and subsequent expansion are shown. Depending on the initial cell-cluster size, organoids differ in the time they need to establish a lumen. The changing colours indicate a newly segmented object at each time point. The segmentation and tracking serves only to visualise the behaviour of the organoids on a single cell level and is neither evaluated nor manually curated. Therefore, a segmented cell can disappear because of a mis-segmentation or the cell loses its signal upon cell death. Microscope: Zeiss Lightsheet Z.1; detection objective: W Plan-Apochromat 20x/1.0, illumination objective: Zeiss LSFM 10x/0.2; laser lines: 488 nm, 561 nm; filters: laser block filter (LBF) 405/488/561; voxel size: 1.02 × 1.02 × 2.00 μm^**3**^; recording interval: 30 min; 3D rendering and tracking software: Arivis Vision4D. (MP4 88,754 kb)

**Fig. 2:**
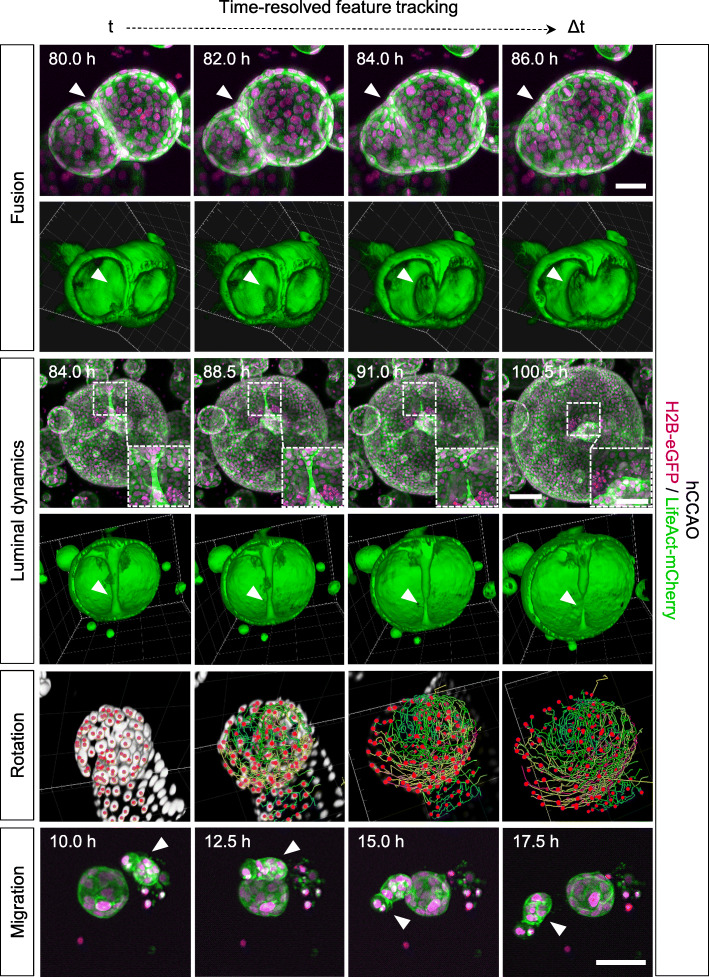
High-quality live LSFM image data provide an excellent basis for volume rendering and detailed feature tracking. It can be used for the quantitative description of cellular dynamics in organoid development. 3D renderings offer detailed views into processes such as organoid fusion and elucidate the spatial context in observed luminal dynamics. 3D cell tracking reveals the complex rotation of the epithelial cell monolayer. hCCAOs (seeded and maintained in Z1-FEP-cuvettes) expressed the nuclei marker H2B-eGFP (magenta) and the F-actin cytoskeletal marker LifeAct-mCherry (green). The figure shows segmented and tracked cell nuclei (Rotation; centroids—red; tracks—rainbow), excerpts of maximum intensity *z*-projections and 3D renderings of corresponding data sets. Segmentation, tracking, and 3D rendering were performed with Arivis Vision4D. Microscope: Zeiss Lightsheet Z.1; objective lenses: detection: W Plan-Apochromat × 20/1.0, illumination: Zeiss LSFM × 10/0.2; laser lines: 488 nm, 561 nm; filters: laser block filter (LBF) 405/488/561; voxel size: 1.02 × 1.02 × 2.00 μm^3^; recording interval: 30 min; scale bars: Fusion, Migration—50 μm, Luminal dynamics—100 μm, 50 μm (inset)


Image-based segmentation and three-dimensional (3D) volume rendering of the acquired data allow for even more detailed inspections of features observed in the highly dynamic organoid system. While most processes can already be followed in maximum intensity *z*-projections of the acquired image data, 3D volume rendering facilitates a more detailed understanding of the underlying cellular dynamics from different perspectives. We identified organoid fusion to be a frequent phenomenon in the investigated cultures (Fig. 2, Fusion; Additional file 17: Video 3). After the epithelial monolayers of both organoids touch each other, they initiate an opening connecting both lumens. This opening then expands while cells migrate into one connected monolayer. Similar dynamics of cells migrating into one connected monolayer were observed in the formation, subsequent retraction and eventual rupture of duct-like structures within the lumen of a large organoid (diameter ≥ 500 μm), which presumably emerged from fusion of multiple organoids (Additional file 1: Definitions) (Fig. 2, Luminal dynamics – *lower panel*; Additional file 18: Video 4, Additional file 19: Video 5). In other examples, single organoids show similar behaviours which can be described as luminal constriction, spontaneous “budding” or duct-like structure formation (Additional file 8: Fig. S7 and Additional file 9: Fig. S8). Volume rendering of cell nuclei revealed that small organoids (diameter at end of observation (48 h): 100 μm) with large nuclei (longest axis: 52 μm) show less cell divisions and overall less cell movement than larger organoids (diameter at end of observation (48 h): 180 μm) with smaller nuclei (longest axis: 32 μm) (Additional file 20: Video 6). This further underlines the heterogeneity in the investigated organoid systems.

**Additional file 17:** **Video 3.** Time-resolved 3D volume rendering of the fusion process of two organoids. hCCAOs expressing H2B-eGFP as nuclei marker (red) and LifeAct-mCherry as F-actin cytoskeletal marker (green) were recorded in a Z1-FEP-cuvette for 5 days. The movie shows an excerpt of the recorded data of 12 h spanning the fusion process of two organoids. The fusion process is visualised by 3D volume rendering of the data acquired for the cytoskeletal marker (LifeAct-mCherry – green). After the epithelial monolayers of both organoids touch, they begin to form an opening connecting both lumens within one hour. This opening then expands while cells migrate into one connected monolayer. Microscope: Zeiss Lightsheet Z.1; detection objective: W Plan-Apochromat 20x/1.0, illumination objective: Zeiss LSFM 10x/0.2; laser lines: 488 nm, 561 nm; filters: laser block filter (LBF) 405/488/561; voxel size: 1.02 × 1.02 × 2.00 μm^3^; recording interval: 30 min; 3D rendering software: Arivis Vision4D. (MP4 37,946 kb)


**Additional file 18:** **Video 4.** Time-resolved 3D volume rendering of intra-organoid luminal dynamics. hCCAOs expressing H2B-eGFP as nuclei marker (red) and LifeAct-mCherry as F-actin cytoskeletal marker (green) were recorded in a Z1-FEP-cuvette for a total of 132 h. The movie shows data recorded between 84 and 108 h. Luminal dynamics are visualised by 3D volume rendering of the data acquired for the cytoskeletal marker (LifeAct-mCherry – green) of a large organoid (diameter: ≥ 500 μm), presumably formed after fusion of multiple organoids. We can follow the formation, subsequent retraction and eventual rupture of duct-like structures within the organoid’s lumen. Microscope: Zeiss Lightsheet Z.1; detection objective: W Plan-Apochromat 20x/1.0, illumination objective: Zeiss LSFM 10x/0.2; laser lines: 488 nm, 561 nm; filters: laser block filter (LBF) 405/488/561; voxel size: 1.02 × 1.02 × 2.00 μm^3^; recording interval: 30 min; 3D rendering software: Arivis Vision4D.


**Additional file 19: Video 5.** Time-resolved 3D volume rendering of a growing liver organoid with cell segmentation and tracking. hCCAOs expressing H2B-eGFP as nuclei marker (red) and LifeAct-mCherry as F-actin cytoskeletal marker (green) were recorded in a Z1-FEP-cuvette for a total of 132 h. The movie shows data recorded between 84 and 108 h. Red spheres illustrate tracked cell nuclei and rainbow-coloured lines indicate the travelled tracks (colour code: red to blue – timepoint 84 to 108). Single as well as multiple organoid tracking is shown. Rotation as well as uni-directional cell movements are visible. Microscope: Zeiss Lightsheet Z.1; detection objective: W Plan-Apochromat 20x/1.0, illumination objective: Zeiss LSFM 10x/0.2; laser lines: 488 nm, 561 nm; filters: laser block filter (LBF) 405/488/561; voxel size: 1.02 × 1.02 × 2.00 μm^**3**^; recording interval: 30 min; 3D rendering and tracking software: Arivis Vision4D. (MP4 36,759 kb)


**Additional file 20: Video 6.** Alterations in rotation velocity of neighbouring organoids. Video shows two mPOs as an excerpt from an entire culture grown within one Z1-FEP-cuvette. The organoids expressed Rosa26-nTnG (grey) as nuclei marker and were imaged over 20 h. Besides the differences in nuclei size, both organoids display different behaviour. Cell tracking revealed a rotational motion of the epithelial cell monolayer of the organoid with the small roundish nuclei and no rotational motion of the organoid with the big, elongated cell nuclei. The two organoids are in close contact but do not fuse or interact with each other. Microscope: Zeiss Lightsheet Z.1; detection objective: W Plan-Apochromat 20x/1.0, illumination objective: Zeiss LSFM 10x/0.2; laser lines: 488 nm, 561 nm; filters: laser block filter (LBF) 405/488/561; voxel size: 1.02 × 1.02 × 2.00 μm^**3**^; recording interval: 30 min; 3D rendering and segmentation software: Arivis Vision4D. (MP4 61,855 kb)

The observed heterogenic dynamic processes in organoids and organoid systems were further visualised using feature tracking tools. Single-cell tracking revealed that the previously observed cell movement in larger organoids with smaller cell nuclei can be described as a uniform rotation of the epithelial cell monolayer (Fig. 2, Rotation; Additional file 18: Video 5, Additional file 20: Video 6). Furthermore, we observed that prior to organoid formation, some of the initially seeded cell clusters migrate through the ECM before they start to form a spherical structure (Fig. 2, Migration; Additional file 21: Video 7). In this example (Additional file 21: Video 7), the cell cluster travels at an average speed of 10 μm per hour (maximum speed: 23 μm per hour), covering a distance of about 250 μm in total. Additional examples of all described processes occurring in hCCAO and mPO cultures are displayed in Additional files 6-9: Figs. S5-S8.


**Additional file 21: Video 7.** Organoid cell cluster migration prior to organoid formation Video shows one mPO as an excerpt of an entire culture grown within one Z1-FEP-cuvette. The organoids expressed Rosa26-nTnG (grey) as nuclei marker and were imaged over 20 h. Prior to organoid formation, the initially seeded organoid cell cluster migrates through the ECM for about 25 h before the cells rearrange to form a spherical structure. The migrated distance is about 250 μm with an average speed of 10 μm (from green/minimum to red/maximum: 2.5 μm/h – 23 μm/h). Microscope: Zeiss Lightsheet Z.1; detection objective: W Plan-Apochromat 20x/1.0, illumination objective: Zeiss LSFM 10x/0.2; laser lines: 488 nm, 561 nm; filters: laser block filter (LBF) 405/488/561; voxel size: 1.02 × 1.02 × 2.00 μm^**3**^; recording interval: 30 min; 3D rendering and tracking software: Arivis Vision4D. (MP4 8503 kb)


**Additional file 22: Video 8.** Exemplary segmentation and tracking of the formation process of organoids. Video shows an mPO as an excerpt of an entire culture grown within one Z1-FEP-cuvette. The organoids expressed Rosa26-nTnG (grey) as nuclei marker. The movie shows an excerpt of the first 10 h a recorded data set of 6 days. The formation starts with a conglomeration of the cells towards one compact structure and ends with the establishment of a lumen. Each coloured dot represents one cell nucleus, but not each nucleus was tracked (lines). To ensure a proper segmentation and tracking higher resolved images need to be acquired, e.g. detection objectives with a higher magnification can be used. Microscope: Zeiss Lightsheet Z.1; detection objective: W Plan-Apochromat 20x/1.0, illumination objective: Zeiss LSFM 10x/0.2; laser lines: 488 nm, 561 nm; filters: laser block filter (LBF) 405/488/561; voxel size: 1.02 × 1.02 × 2.00 μm^**3**^; recording interval: 30 min; 3D rendering and tracking software: Arivis Vision4D. (MP4 11,909 kb)

### Long-term single-cell analysis of pancreas-derived organoids reveals cell-to-cell heterogeneity in cell proliferation

Next, we aimed for a deeper quantitative analysis of the dynamic cellular processes in luminal expansion, specifically regarding the observed size oscillations. The collected high-resolution LSFM images enabled the semi-automatic segmentation and quantitative feature extraction over the course of a 6-day acquisition. This provided robust, time-resolved data on cell nuclei numbers, organoid volume, surface area, the number of neighbouring cells for each cell, and the cell density.

**Fig. 3:**
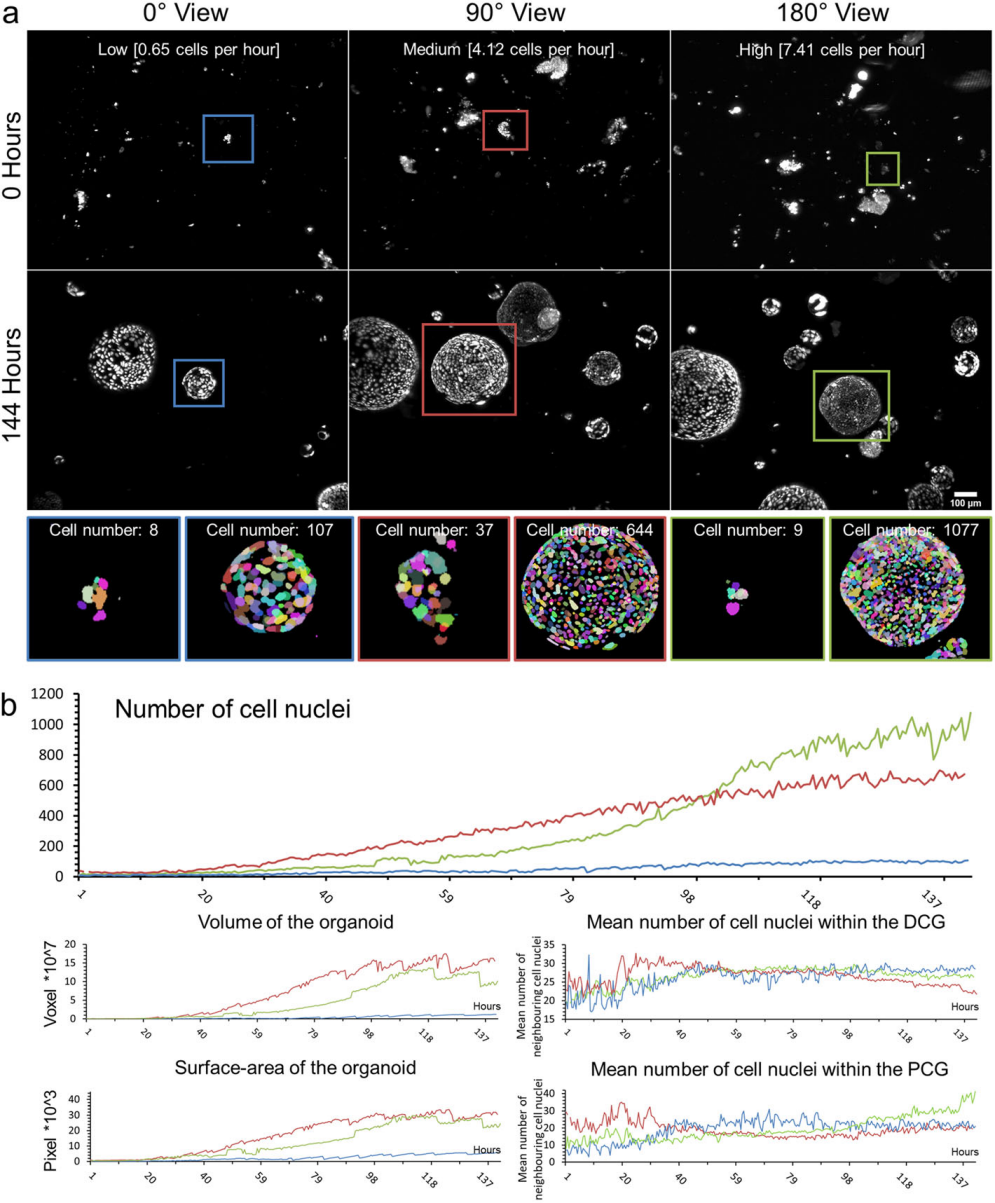
Long-term single-cell analysis of mPOs reveals heterogeneity of proliferation potentials. mPOs (seeded and maintained in one Z1-FEP-cuvettes) expressed the nuclei marker Rosa26-nTnG (grey). Organoids were imaged for 6 days and analysed with our previously published nuclei segmentation pipeline [22]. **a** Three representative organoids are shown directly after seeding (0 h, upper row) and after 6 days (144 h, lower row). Every row shows one view of the same Z1-FEP-cuvette. Hence, all displayed organoids were grown simultaneously within one FEP-cuvette. The close-ups display the segmentation of the organoid at the corresponding time point. Different colours refer to individual cell nuclei. The coloured frames indicate organoids with different proliferation rates—green/high, blue/low, and red/medium. **b** From top to bottom, corresponding evaluations of volume, surface area, and neighbourhood relationships (DCG: Delaunay cell graph; PCG: proximity cell graph). Microscope: Zeiss Lightsheet Z.1; objective lenses: detection: W Plan-Apochromat × 20/1.0, illumination: Zeiss LSFM × 10/0.2; laser lines: 561 nm; filters: laser block filter (LBF) 405/488/561; voxel size: 1.02 × 1.02 × 2.00 μm^3^; recording interval: 30 min; scale bar: 100 μm

Using our previously published segmentation pipeline [16, 22], we processed one time-lapse dataset of an mPO culture, which resulted in a total number of 288 segmented time points. To evaluate the segmentation performance, we generated ground truth data sets to estimate the F score (0.74 to 0.83, Additional file 10: Fig. S9, Additional file 23: Table S2). From the segmented data, we chose to analyse three representative organoids. One small organoid (diameter < 400 μm), one large organoid (diameter > 400 μm), and one which was size-comparable to the large organoid but showed a higher cell number. The three mPOs expressed Rosa26-nTnG as a nuclei marker (Fig. 3).

We observed that in individual organoids, the number of cells increases at different rates even if they have similar initial cell numbers. We show that an organoid with an initial cell number of eight increases at a low rate (average: 0.65 cells per hour) and reaches a maximum number of 107 cells after 6 days, whereas an organoid starting with nine cells increases at a high rate (average: 7.41 cells per hour) and ends up with 1077 cells after 6 days (Fig. 3a, blue and green frames). Since the splitting procedure results in different sizes of cell clusters, we also analysed one organoid that started with 37 cells and reaches a total number of 644 after 6 days (average: 4.12 cells per hour) (Fig. 3a, red frames).

To further understand the heterogeneity in proliferation potential and the collective cell behaviour in general, we also quantified the volume and surface area of the organoid as well as the neighbourhood relationships of the single cells (Fig. 3b). We observed that the organoid with the largest final number of cells did not show the largest volume and surface area (final cell number: 1077, final volume: 10 × 10^7^ voxels, final surface area: 24 × 10^3^ pixels, Fig. 3b, green lines). In this organoid, the mean number of neighbouring cells was higher within the proximity cell graph (PCG), meaning that cells are neighbours if they are closer than a certain distance (distance: 50 pixels, final PCG-value: 35), in comparison to the organoid with the largest volume and surface area (final PCG-value: 19, final cell number: 644, final volume: 15 × 10^7^ voxels, final surface area: 30 × 10^3^ pixels, Fig. 3b, red lines). These findings correlate with different cell densities, displayed by the number of neighbouring cells within the Delaunay cell graph (DCG) of 26 and 21 respectively.

**Fig. 4:**
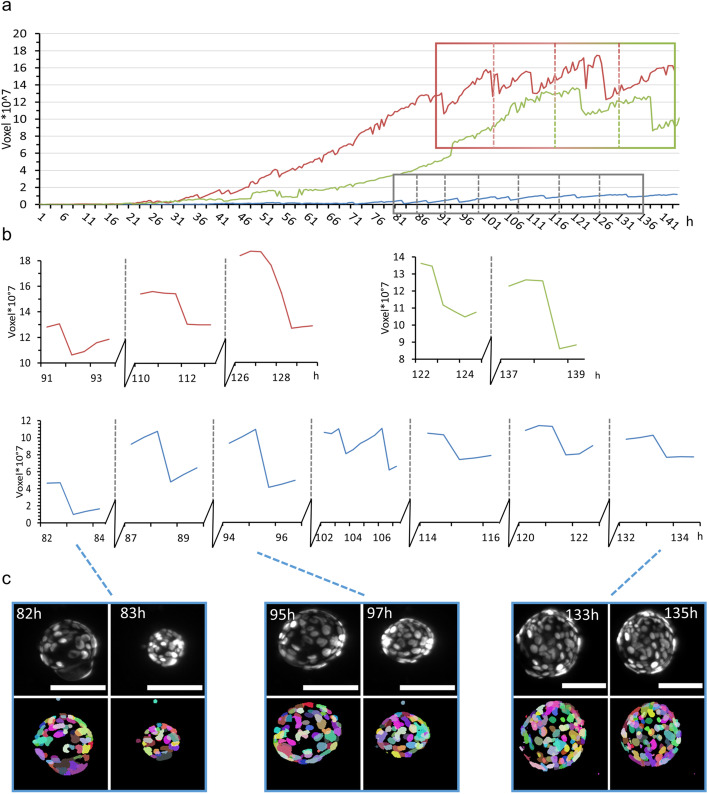
Volume analysis of three representative mPOs reveals different oscillation frequencies*.* MPOs (seeded and maintained in one Z1-FEP-cuvettes) expressed the nuclei marker Rosa26-nTnG (grey). Organoids were imaged for 6 days and three representative organoids were analysed with our previously published nuclei segmentation pipeline [22] in regard to size oscillation events. A size oscillation lasts between 30 min and 2 h. **a** Volume over time for each organoid approximated from the cell nuclei segmentation. **b** Close-up of three (red), two (green) and seven (blue) size oscillation events with more than 5% volume reductions are shown. **c** Typical images of organoid size oscillation. The upper row shows the nuclei in grey in the raw image, the lower row the segmented cell nuclei. Each colour illustrates a single-cell nucleus. Microscope: Zeiss Lightsheet Z.1; objective lenses: detection: W Plan-Apochromat × 20/1.0, illumination: Zeiss LSFM × 10/0.2; laser lines: 561 nm; filters: laser block filter (LBF) 405/488/561; voxel size: 1.02 × 1.02 × 2.00 μm^3^; recording interval: 30 min; scale bar: 100 μm

All three organoids showed frequent size oscillations (Fig. 4). Thus, we analysed the size oscillations based on the organoid volume (Fig. 4c shows an example of size oscillation in the segmented data). However, over the time course of 6 days, the small organoid (Fig. 4a, b, blue lines) showed seven oscillations, whereas the two larger organoids showed three (Fig. 4a, b, red lines) and two (Fig. 4a, b, green lines) events, respectively. Interestingly, we did not observe any correlation between the size oscillation events and the changes in cell number or number and distance of neighbouring cells. Further, we did not observe any size oscillation events in the first 80 h (within the 5% threshold) nor did we observe any synchronised oscillation behaviour between the organoids within one culture.

### Scaling law derived from simplifying assumptions indicates a dependence of size oscillation events on cell division dynamics

To solve the mechanical principles underlying size oscillation events, we developed a mathematical model based on the following assumptions. Since organoids are spherical single-layer multicellular clusters, they are described by their volume *V*(*t*) and the number of superficial cells *N*(*t*) at time point *t*. We propose a functional relationship for an organoid’s increase in volume $\dot{V}(t)$, which is derived from two processes: (a) The internal pressure of an organoid increases with time, due to an influx following the segregation of an osmotic active substance by the cells. (b) Due to mitosis, the cell number *N*(*t*) grows and the surface area *A*(*t*) increases (Fig. 1, Cell division).

We hypothesise that the increase of the cell number $\dot{N}(t)$ can balance the increase in inner pressure of an organoid and prevent size oscillation events. In the following, we show that this requires the cell count *N*(*t*) to grow faster than or equal to *N*(*t*)~*t*^2^. In return, we expect the occurrence of size oscillations in the case where the cell number increases slower than *N*(*t*)~*t*^2^. Our estimation is based on the following relations and simplifying assumptions:

i. Organoids form spheres with a volume of
  $$ V=\frac{1}{6}\pi \times {d}^3 $$

and a surface area of

$$ A=\pi \times {d}^2 $$

The relation between volume and surface area can be written as

$$ V\sim {A}^{3/2} $$

ii. Every cell produces substances, which are secreted into the lumen of the organoid. We assume that the production rate is constant in time and the same for all cells, and therefore proportional to the number of superficial cells.

$$ \dot{n}(t)\sim N(t) $$

iii. We further assume that the relation between secreted substance and osmotic pressure (Π) follows the van-‘t-Hoff law

$$ \Pi =c\times {i}_{vH}\times R\times T=\frac{n}{V}\times {i}_{vH}\times R\times T\sim \frac{n}{V} $$

iv. Because of cell division, the surface area grows as a function of time, *A* = *A*(*t*). We neglect cell growth and assume that the cell count *N*(*t*) is proportional to the surface of the organoid, *A*(*t*).

v. The total amount of the substance inside the lumen, *n*, is the accumulated substance produced during organoid growth, therefore

$$ n\sim \int A(t) $$

This gives us the relation

$$ \Pi \sim \frac{\int A(t)}{A{(t)}^{3/2}} $$

In order to avoid a rupture, the growth of the surface *A*(*t*) has to balance the resulting osmotic pressure Π arising from constant production of *n* by *A*. We can compute the functional form of *A*(*t*) which leads to a constant osmotic pressure Π. We require

$$ \frac{\int A(t)}{A{(t)}^{3/2}}=\mathrm{const}. $$

This relationship is fulfilled when *A*(*t*)~*t*^2^. This scaling law provides the following direct implications: A constant cell division rate causes the cell count to increase exponentially. Exponential growth is faster than quadratic; due to the theoretical considerations here we expect no rupture and subsequent size oscillation events. Some of the organoids, however, show a quasi-linear increase in cell numbers, which corresponds to a mitosis rate that is decreasing with 1/*t*. A linear increase is slower than *t*^2^; hence, in these organoids, we expect rupture and size oscillations.

In addition, we point out that the surface to volume ratio of a sphere changes with the radius, since the volume grows much faster than the surface. Since the osmotically active substance in the lumen is produced by the surface, this implies that smaller organoids reach a critical internal pressure earlier than large organoids.

### Agent-based mathematical model captures the experimental organoid dynamics and confirms theoretical considerations

**Fig. 5:**
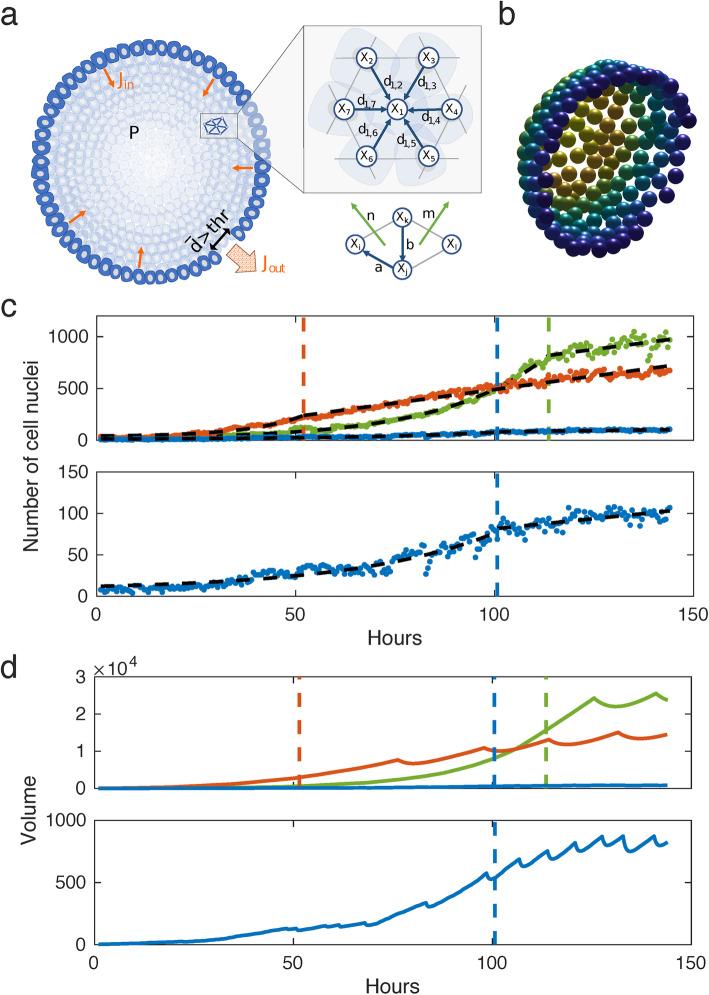
Computational simulation of a multi-agent object. **a** Illustration of the general model. **b** Snapshot of a simulated sphere cut in half. **c** Piecewise exponential-linear fit (black dashed lines) to the growth rates for the long-term single-cell analysis of mPOs. The colours resemble organoids shown in Fig. 3. The dots indicate the cell count of the organoids. The coloured dashed lines indicate the transition from exponential to linear growth. **d** Volumes of the simulated organoids. The coloured dashed lines indicate the transition from an exponential to a linear growth rate. The coloured solid lines show the volumes of the spheres

In order to confirm our hypotheses, we developed a mechanical 3D agent-based model for organoid size oscillations, based on the experimental data obtained by long-term single cell analysis of mPOs (Fig. 5a; Additional file 11: Fig. S10).

**Fig. 6:**
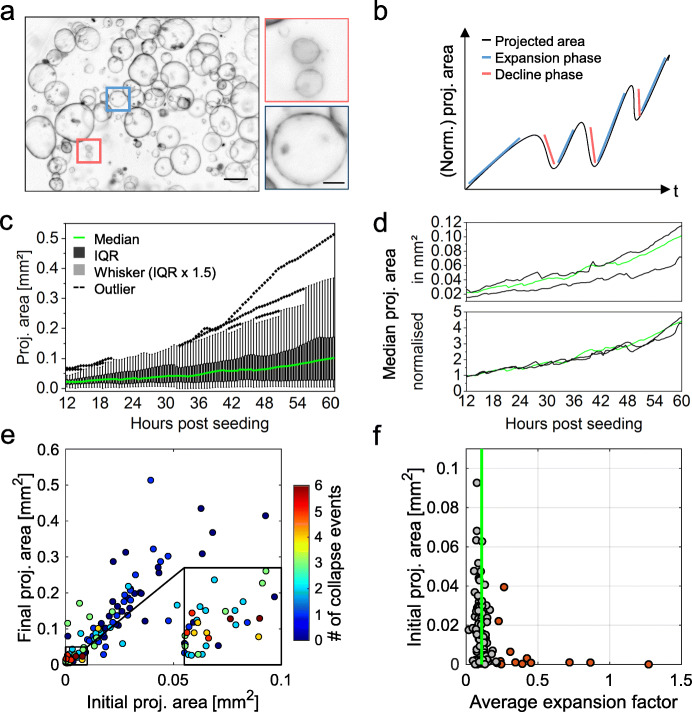
Analysis of multiple monocystic mPOs reveals heterogeneity as well as core regulatory principles. **a** Overview bright-field images of mPOs displaying a monocystic phenotype. Microscope: Zeiss Axio Observer Z.1; objective lenses: Plan-Apochromat × 5/0.16, avg. *z*-projection, voxel size: 1.29 × 1.29 × 50 μm^3^, scale bar overview: 500 μm, close-up: 25 μm. **b** Schematic plot of feature extraction based on time-resolved bright-field images. Multiple features, such as the projected luminal area, expansion phases, and size oscillation events, were analysed. **c** The projected areas of single organoids growing within one well were analysed for 48 h, starting 12 h after seeding and revealed high heterogeneity in the projected areas. A high intercultural heterogeneity is illustrated by a broad inter quartile range (black) and outliers (dashed lines) within the box plot (*n* = 34). **d** Medians of projected areas of three wells (technical replicates) differ. The medians of the normalised projected areas are coherent between individual wells. Median shown in **c** is highlighted in green (*n* = 34, 31, 35). **e** The colour code signals the amount of registered size oscillation events. Smaller organoids display an increased number of oscillation events. Close-up reveals location of organoids collapsing four to six times (*n* = 100). **f** The median of the average expansion factor is 0.11 (green line). Independent of their initial projected area [mm^2^], 50% of all organoids display an average expansion factor between 0.09 and 0.14. Here, 12% of the organoids feature an average expansion factor above 0.22 and are marked as outliers (red) (*n* = 100)

We hypothesise that the organoids can be represented as elastic spheres with a growing surface due to cell division (Fig. 5b). The agents represent single cells applying mechanical forces onto neighbouring cells. Concerning the mechanical properties of the cells, we used a general framework describing epithelial cell-cell interaction, which was previously used to resolve the underlying dynamics of lung cancer cell migration and local cell fate clustering in inner cell mass organoids [25, 30]. We hypothesise that the observed polarisation of the cells (Fig. 1) is maintaining the spherical shape of the organoids. Thus, a bending potential is added to the model. Based on the findings of Ruiz-Herrero et al. [24], we assume that the cells continuously secrete a substance into the lumen, which leads to an osmotic influx. This influx leads to an increase in the internal pressure which, however, can be balanced by an increase in volume. When the average distance of neighbouring cells exceeds a certain limit, the organoid shell ruptures, leading to a substantial outflow of liquid and deflation of the organoid. Following the law of parsimony, we consider the mechanical properties, cell size, and each cell’s contribution to the osmotic influx to be homogeneous for all organoids. In agreement with the data, cell division dynamics in the simulations differ between the organoids, but are also assumed homogeneous within the same organoid. Based on these assumptions, we derived that the organoid can balance the inner pressure when the cell count increases at least quadratically (Additional file 24: Supplementary theoretical considerations). Furthermore, for small organoids, the ratio between surface and volume is smaller than for large organoids. Therefore, small organoids should reach a critical pressure for leakage faster than large organoids. Thus, we expect the size oscillations to critically depend on (a) the cell division dynamics and (b) the organoid size. The latter (b) is confirmed by the data obtained through bright-field analysis (Fig. 6e).

The model is used to support the theoretical considerations and to qualitatively reproduce the size oscillations of the three analysed mPOs (Fig. 4a, b). Hereby, the cell division rate is directly extracted from the experimental data (Fig. 3b). Simulations of two large organoids do not show a size oscillation during phases of exponential cell number increase but start to oscillate after transitioning to a linear growth (Fig. 5c, d, green and red lines). Simulations with a small organoid exhibit size oscillation even during the initial exponential growth, which confirms our hypothesis that small organoids are more prone to rupture and deflation (Fig. 5c, d, blue lines; Fig. 6e).

Hence, the simulation results show a large qualitatively agreement in the size oscillations with the experimental data and also coincide with the analytical results.

### Time-resolved macro- and mesoscale analysis reveals organoid-to-organoid heterogeneity as well as core regulatory principles

The processes observed using LSFM suggest a vast variety of complex dynamic processes in organoid cultures. In order to analyse the growth characteristics on a macroscale level and to confirm the predictions suggested by the computational model, we established a pipeline based on time-resolved bright-field observations. The analysis allows to characterise a culture’s global behaviour. Via semi-automated watershed-based segmentation, the pipeline allows for quantification of the projected luminal areas [mm^2^] over time of several organoids in parallel (Additional file 2: Fig. S1). Subsequently, from the normalised projected areas, the relative size increase is evaluated. Further, expansion phases (timing, slope, duration), size oscillation events (timing, slope, duration), and minimum and maximum projected luminal areas are identified for individual organoids (Fig. 6b).

In Fig. 6c, the projected areas of 34 pancreas organoids growing within one well are plotted over 48 h. The projected areas illustrate the high heterogeneity, with an area distribution widening over time. After 48 h of observation, the projected areas have a median of 0.1 mm^2^, while their interquartile range (IQR) ranges from 0.03 to 0.17 mm^2^.

Further, we demonstrate that the bright-field pipeline provides consistent and robust growth analysis data in technical replicates (Additional file 1: Definitions) (three wells, *n* = 34, 31, 35) (Fig. 6d). The median values of the normalised projected areas show no significant differences between the technical replicates (Kruskal-Wallis ANOVA).

The extracted features can further be used in downstream analyses to categorise organoid behaviour. We found that the size of an organoid is crucial for the number of oscillation events it displays. The bright-field analysis shows that over the observation period the organoids increase their projected area approximately to the six-fold area. Initially smaller organoids (area < 0.01 mm^2^) feature more size oscillation events, while initially larger organoids display less oscillation events (area > 0.01 mm^2^) (Fig. 6e). This coincides with the mathematical considerations that the surface to volume ratio is important for the oscillating behaviour of the organoids. To test whether the mathematical model can also reproduce this observation, we use the initial and final size of the organoids which are known from the experiments. Since no cell division dynamics are extracted from the bright-field pipeline, we assume for simplicity a linear increase in the cell numbers for all simulated organoids. In this setting, the model strongly agrees with the experimental data, reproducing the tendency that initially smaller organoids display an increased number of size oscillation events (Additional file 12: Fig. S11). Thus, the model confirms an influence of organoid size and growth onto the oscillation events. Besides that, the average expansion factor (a measure for expansion speed consistency) with a median average value of 0.11 is similar between organoids with various initial areas—50% of all values range between 0.09 and 0.14, while only 12% of the evaluated organoids are outliers with values above 0.22 (Fig. 6f). Further, the linear correlation between the initial area and the final area becomes apparent (*R*^2^ = 0.7445), which shows that the growth is independent of the initial area (Additional file 13: Fig. S12a). This indicates strong similarities in expansion speed consistency between individual organoids within one culture despite their (high) size heterogeneity.

Besides the already mentioned features, other extracted features facilitate the definition of quantitative reference parameters of organoid systems. By comparing the final area to the maximum area, for example, continuous growth of mPOs during the analysed time window is proven. A comparison of the initial area to the minimum area identifies size oscillation events or overall descending size progression within organoid cultures. In mPOs, the minimum area falls only slightly below the initial area, which can be associated with oscillation events (Additional file 13: Fig. S12c). Besides the average expansion factor, analysis of the maximum expansion factor indicates expansion speed variations within organoid cultures. As a variable factor, the maximum expansion can be used to compare different culture conditions (Additional file 13: Fig. S12b). An additional feature, which is likely to change upon differentiation or other perturbations (e.g. drug treatment), is the organoid circularity. In healthy mPOs, the circularity is 0.9 on average and the deviation around the average narrows over time (Additional file 13: Fig. S12e). In addition to the analysis of monocystic epithelial organoids like mPOs, our bright-field pipeline can also be used to analyse deviating organoid morphologies like polycystic hCCAOs (Additional file 14: Fig. S13a-f). Polycystic hCCAOs show an average circularity of 0.8 over the course of 48 h of observation (Additional file 14: Fig. S13b). Therefore, as a general culture feature, the circularity can serve as an additional quality control parameter.

## Discussion

We describe two complementary light sheet and bright-field imaging pipelines for the time-resolved, multiscale quantitative analysis of single-cell and collective cell behaviours in organoids. The goal is the characterisation of the heterogeneity of organoid cultures in their entirety.

**Fig. 7:**
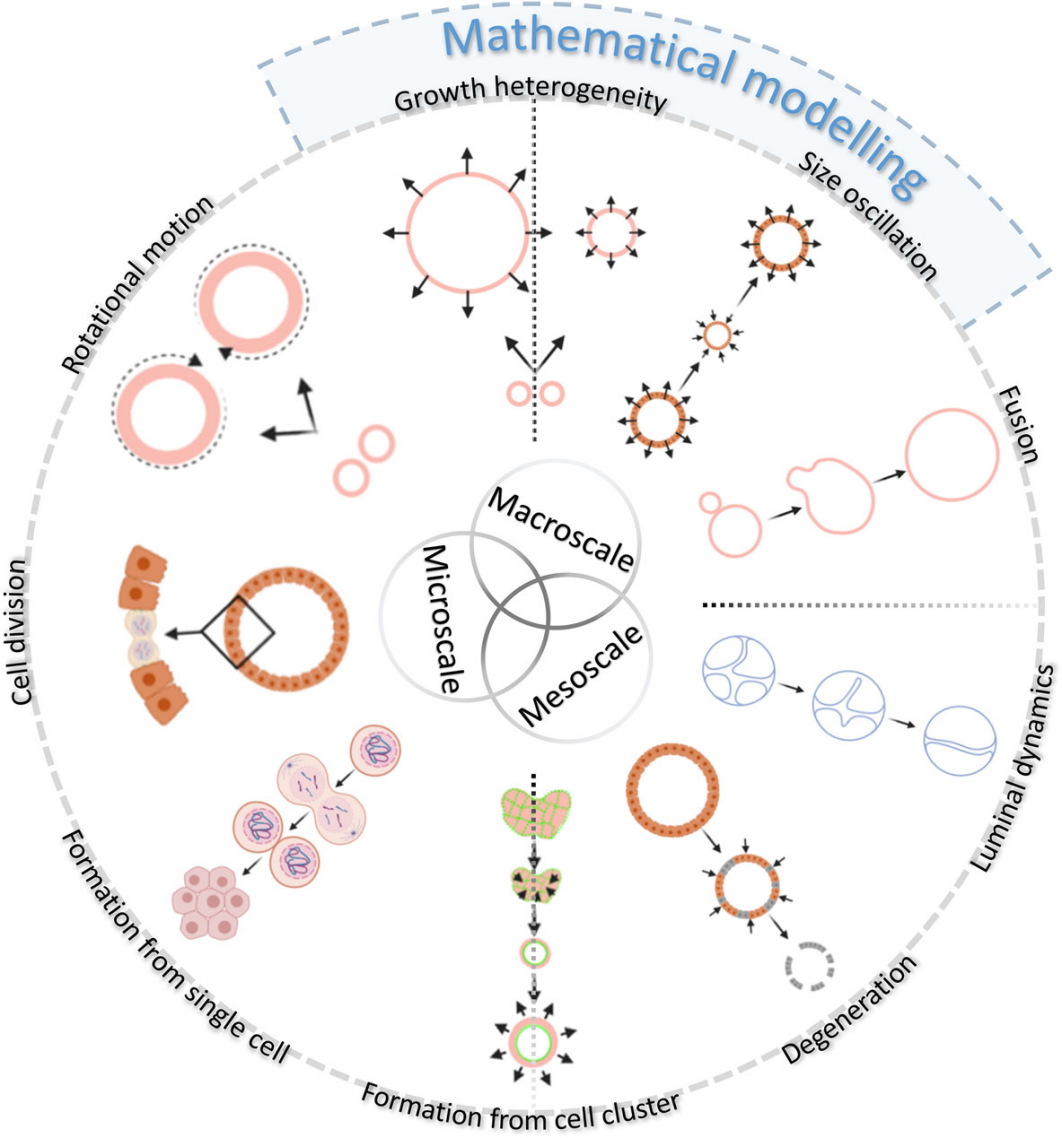
The infographic shows an overview of all the observed organoid dynamics at multiple scales. The organoids display a wide range of motional and morphogenetic events, at single-cell (microscale), individual-organoid (mesoscale), and at entire-culture level (macroscale). In addition, the infographic shows the events that have been mathematically modelled. Graphic created with BioRender.com

The light sheet pipeline led to the identification of several dynamic processes typical of organoid cultures on micro- (single-cell) and mesoscale (individual-organoid) levels. The resolution and contrast of the light sheet images allowed the quantification and qualitative visualisation of these processes with nuclei segmentation and single-cell tracking. The bright-field pipeline allowed quantifying the dynamics of individual organoids as well as of entire organoid cultures on a macroscale level. An infographic summarising all the observed organoid dynamics is shown in Fig. 7.

In both pipelines, we choose a time resolution of 30 min to capture the growth behaviour of organoids. We selected this time interval as a technical compromise in the need for a high temporal resolution to resolve dynamic processes (e.g. budding or cell rearrangement processes [8]), and the need to observe these processes over long observation periods (e.g. to study cell differentiation [14, 31]). Thereby, the segmentation of individual time points achieves F scores between 0.74 and 0.83. Compared to previously published segmentation of fixed and stained samples, the F score is lower [21, 22]. However, taking into account the uneven fluorescence signal in living samples, the large number of individual cells per organoid and the multitude of analysed time points (with the same settings), the F score is acceptable.

In previous studies, chromosomal segregation errors in organoids were monitored using confocal or spinning disc microscopy, capturing single organoids in a *z*-range of 60 μm (3–4 min intervals) [12]. The observation of single mitotic events with LSFM over multiple days, from the initial seeding to the plateau phase of growth, can therefore increase the throughput and allow the analysis of larger organoids to monitor single cell behaviour. Serra et al. used an inverted LSFM to analyse the development of single organoids originating from single cells. By parallelisation, they were able to image multiple organoids [8]. Our light sheet pipeline combines a parallelised acquisition of more than 100 organoids, within a volume of up to 8 mm^3^ given by the Z1-FEP-cuvette, with a high spatial (1000 *z*-planes, 2 μm spacing), as well as a high temporal resolution and still allows long-term observations. To demonstrate the feasibility of cell tracking, we utilised the commercially available software Arivis Vision4D in a qualitative approach to visualise rotation in some of the organoids. When tracking cell movements during formation however, the setup is still not adequate and needs adaptations (Additional file 22: Video 8). Since not all cells can be segmented and tracked, we would recommend a higher magnification lens (e.g. × 63) and a higher temporal resolution to improve the segmentation and allow the detailed analysis of the formation process as well. Additionally, manual curation and a validation of the Arivis Vision 4D tracking would be needed to reduce artefacts and quantify the tracking for downstream modelling.

Our analysis of mPOs and hCCAOs reveals their highly heterogeneous and multi-faceted growth patterns and common morphological dynamics independent of their carcinogenic or healthy origin. This matches the observation of intrinsic abilities of single intestinal organoid cells to form asymmetric structures [8], as well as former studies that have not addressed heterogeneity directly, but already showed variable organoid sizes and irregularly occurring rupture events [32–35]. As mentioned, mPOs and hCCAOs are derived from adult ductal cells. Both cell types reside within the mature organ and contribute to regeneration after extensive or chronic injury [36]. As liver and pancreas emerge from the same layer of endoderm during embryonic development, we assume that the tissue-specific progenitor cells still share some characteristics, as cell migration and epithelial rearrangements are concerned.

Adult tissue-derived organoids can develop from single cells or cell clusters, although starting from single cells results in lower organoid formation efficiency, which hampers the systematic analysis of cellular behaviours [8]. Starting from cell clusters, the cell survival and therefore the multiplication rate of cell material is higher. The production of large amounts of material for a potential clinical application is therefore ensured [37]. However, starting from clusters, the heterogeneity of the culture dynamics increases. Since our pipelines capture both aspects, they support the understanding of clonal formation as well as the determination of quality control parameters for clinical applications of organoids. We illustrate at multiple scales that frequent size oscillations in mPOs are common during the period of growth. Size oscillation was also more sporadically observed in the hCCAOs (Fig. 1, Size oscillation). The change of luminal volume in organoids is poorly investigated. So far, indications are growing, that it is determined by ions and liquid flow. Ion channels are the main drivers of lumen variations. For instance, the cystic fibrosis transmembrane conductance regulator (CFTR) channel triggers fluid secretion in the luminal compartment leading to a fast enlargement of the lumen. Since cystic fibrosis patients have in many cases impaired or not functional CFTR, an organoid-based assay has been developed to identify which patients are responsive to a CFTR-rescuing pharmacological therapy [5]. In general, a closed single-layer epithelium forming a lumen is one basic building-block of many epithelial organs. Lumen enlargement is an essential step in ductal and acinar epithelial morphogenesis [38]. The mechanism of luminogenesis has been extensively investigated with 3D-cultured MDCK cells (MDCK: Madin-Derby Canine Kidney cell line) [39]. The mechanical cues mediated by lumen enlargement and oscillations are also essential during embryo development [40]. Strikingly, the release of the blastocyst from the zona pellucida is driven by an active swelling and shrinking of the lumen. In our investigation, we were able to illustrate that size oscillations occur more often in smaller organoids, showing that the initial size of the cell cluster is a crucial factor. This observation is in contrast to previous findings by Sebrell et al. who observed a trend to more size oscillation in large (> 200 μm) organoids [32].. Since they analysed human gastric epithelial organoids, this raises the question of how organoid systems differ in their behaviour and growth even if they are both of epithelial origin and grown under similar culture conditions. This further illustrates the need to define core regulatory principles of organoid systems.

Furthermore, we observed and defined the fusion process of organoids. This process has never been described for organoids in 3D in comparable temporal and spatial resolution before and shows similarities to several processes in mammalian embryonic development [41] and organ maintenance [42]. Kim et al. analysed the fusion of the palatal shelfs during craniofacial development and identified three general stages for tissue fusion, which are comparable to our observed organoid fusions. It is initialised by the convergence of two epithelial layers, migratory movements towards one epithelial layer and subsequent rupture of the single cell layer. Dumortier et al. showed that the formation of the blastocoel during mouse pre-implantation is a result of a highly dynamic process of cell-cell ruptures and fusion events that lead to a final large lumen [43]. They predict that hydraulic fracturing of cell-cell contacts guides the rupturing process, which is consistent with our mechanical 3D agent-based model and our data. In addition to organoid fusion, we observed and described luminal dynamics within single organoids and presumably upon organoid fusion. Both processes, fusion and luminal dynamics, were frequently observed in different cultures but not often within one replicate. These dynamic behaviours appear to depend on several different factors, which render them difficult to quantify and predict. For instance, the initial seeding density and the resulting proximity of the organoids plays a crucial role in organoid fusion. We hypothesise that a similarity of cell character and rotation behaviour also works in favour for fusion. Consequently, we think that differences in cell character and rotation behaviour hinder the fusion of neighbouring organoids. For luminal dynamics, we assume that the underlying character of cells within one organoid plays a crucial role in the potential of the organoid to constrict its lumen or to form “buds” and duct-like structures. We hypothesise that a strong ductal character is required to evoke these behaviours. To confirm these theories, more analyses (potentially employing specific cellular markers) need to be conducted.

The multi-faceted dynamic behaviour of organoids is reflected in their motion. We were able to show that organoids differ in their overall rotation speed and rotation direction and that not all organoids show rotational behaviour. Indeed, it remains an exciting question, possibly also related to the inter-organoid heterogeneity, why not all investigated organoids show rotation. The rotational motion of organoids has been so far poorly described. Sebrell et al. related the rotation of gastric epithelial organoids to the passage number and patient/donor history [32]. However, epithelial rotation has been investigated in other 3D cell culture systems, such as MDCK cysts [44–47]. Wang et al. investigated the rotation of 3D human mammary epithelial acini and identified that cell polarity and microtubules are essential for rotation [48]. Moreover, epithelial rotation seems to have a function in Drosophila reproductive physiology [49]. In conclusion, organoids display a promising model to further investigate collective cellular rotational behaviour.

Computational and mathematical in silico models are a valuable tool to understand the underlying mechanics of 3D cell culture behaviour [50]. They can be used to predict organoid behaviour in conditions that are challenging to implement in experiments or when perturbations of normal conditions occur [50, 51]. However, only relatively few models for organoid systems have been developed [52]. Here, we implemented a mechanical 3D agent-based model that relies on a limited set of assumptions (namely intercellular forces, internal pressure of the organoid, bending energy of the surface, and cell division). We showed that the model is a valuable instrument for the description of spatio-temporal dynamics of organoids. We were able to recreate the qualitative growth curve of the three segmented organoids and showed that the frequent size oscillation of organoids is not directly associated with mitosis (for further experimental analysis of the mechanisms underlying organoid size changes see also Yang et al. [53]). Instead, the model indicates that the decline process relies on cells losing cell contacts due to mechanical stress exerted by the internal luminal pressure [24]. Further, the model confirms that the size oscillation dynamics are dependent on the organoid volume-to-surface ratio and its dynamics with exponential and linear growth phases. Additionally, the implemented model indicates a coupling of the elasticity of the cells and the formation of the internal pressure. It would, in principle, be possible to estimate one of the parameters if the other is determined experimentally. This is something we would like to address in the future. The disagreement between the simulation and data concerning the volume of the medium and large organoid results from the fact that the cell size differs between the two organoids. The large organoid exhibits a higher cell density, which implies a smaller cell size compared to the medium size organoid. Different cell sizes are not considered in the current version of the model but can be included to reflect these different phenotypes.

Our light sheet pipeline shows that the small organoid has a higher size oscillation frequency (Additional file 1: Definitions) than the larger organoids. The theoretical considerations and the mathematical model support this observation: size oscillations are affected by an increased surface-to-volume ratio. The bright-field pipeline further confirms this observation. In summary, the simulation of epithelial organoid growth predicts organoid behaviour and helps to understand the intrinsic mechanisms responsible for the organoid phenotype. Further, it is straightforward to generate a cost- and time-effective tool to predict possible outcomes of external stimuli like drug treatments for instance [52].

The bright-field pipeline enables the quantification of culture dynamics on meso- and macroscale levels, generating robust data on organoid growth behaviour and allowing the quantification of heterogeneity in whole organoid cultures. The pipeline has been extensively applied in the LSFM4LIFE (www.lsfm4life.eu) and the Onconoid Hub projects to measure and optimise the growth of human pancreas-derived organoids in synthetic hydrogels and identify novel drug candidates for the treatment of intrahepatic cholangiocarcinoma (manuscripts in preparation). In perspective, the same analysis is suitable to determine parameters of organoid growth for stem cell therapy [2, 54–56] and to characterise patient-specific responses for optimising personalised drug treatments or assaying the onset of resistance in cancer therapy [4, 56–58].

## Conclusion

Our multiscale analyses of diverse organoid cultures have great potential for further investigations of epithelial organoids and many other complex culture systems. We were able to define core regulatory principles of epithelial organoid systems and identify serval behavioural patterns, which can reflect embryonic developments or injury-associated healing processes. Organoids can therefore serve as model organisms to gain deeper insights of these processes and help to understand fundamental epithelial rearrangement processes.

## Material and methods

### Organoid culture

hCCAOs were initiated from primary liver tumour biopsies of cholangiocarcinoma patients (~ 0.5 cm^3^) collected during surgery performed at the Erasmus MC – University Medical Center Rotterdam (NL) and cultured as previously described [4]. mPOs were obtained from Meritxell Huch (Gurdon Institute, Cambridge, UK) and cultured as described [59].

### Transgenic murine pancreas-derived organoids

The transgenic mPO line was obtained from Meritxell Huch’s laboratory at the Wellcome Trust/CRUK Gurdon Institute, University of Cambridge, UK. The cells were isolated from the adult pancreas of the Rosa26-nTnG mouse line (B6;129S6-Gt (ROSA)26Sortm1(CAG-tdTomato*,-EGFP*)Ees/J, stock no. 023035, The Jackson Laboratory, Bar Harbour, Maine) according to the isolation protocol [60].

### Viral transduction of human liver-derived tumour organoids

hCCAOs were transduced using a third generation lentivector (pLV-Puro-EF1A-H2B/EGFP:T2A:LifeAct/mCherry, vector ID: IK-VB180119-1097haw, custom-made by and commercially obtained from AMSBIO, Abingdon, UK) for stable expression of the fluorescent fusion proteins H2B-eGFP to visualise cell nuclei and LifeAct-mCherry to visualise the F-actin cytoskeleton. Lentiviral particles were commercially obtained from AMSBIO (Abingdon, UK). Viral transduction of organoids for stable expression of fluorescent markers in hCCAOs was performed according to a protocol published by Broutier et al. [60] with slight modifications. In brief, organoids were dissociated into small cell clusters by mechanical fragmentation in pre-warmed (37 °C) trypsin and subsequent incubation for 5–10 min. All centrifugation steps were carried out at room temperature. Positive (transduced) organoids were selectively picked under semi-sterile conditions instead of being selected by puromycin administration and were expanded into positively labelled cultures without sorting.

### Light sheet pipeline

In order to generate single-cell resolved high-content data of organoid dynamics, the previously published ultra-thin FEP-foil cuvette [16] was used. To implement it into the Zeiss Lightsheet Z.1 system (Carl Zeiss AG, Oberkochen, Germany), a new positive module was produced, and the cuvette was connected with a capillary.

### Fabrication of positive moulds for vacuum forming

We designed positive moulds of the cuvettes for the use in the Zeiss Lightsheet Z.1 system by using the free CAD software “123D Design” (version 2.2.14, Autodesk). We 3D-printed the positive moulds by using the service of the company Shapeways. Before use, the positive moulds were inspected by stereomicroscopy and cleaned by immersion in an ultrasonic bath.

### Cuvette fabrication with vacuum forming

For a detailed description of the cuvette fabrication with vacuum forming refer to Hötte et al. [16]. In brief, a 10 cm × 10 cm squared patch of FEP-foil (50 μm thickness, batch no. GRN069662, Lohmann Technologies, Milton Keynes, UK) was clamped into the vacuum-forming machine (JT-18, Jin Tai Machining Company), heated up to 280 °C and pressed onto the square cross-section positive mould described in Additional file 3: Fig. S2d-e. The positive mould was assembled with a 2 mm × 2 mm 3D-printed square cross-section rod and a glass capillary (borosilicate glass 3.3, material no. 0500, Hilgenberg GmbH, Malsfeld, Germany), cut to a length of about 15 mm with a diamond cutter (Additional file 3: Fig. S2d-e). After vacuum forming of the FEP foil, the square cross-section rod was carefully removed, leaving the FEP cuvette supported by the glass capillary, which serves as mechanically stable connection with the Zeiss Z.1 holder. A shrinking tube (flame retardant polyolefin tube, size 3, cat. no. E255532, G-APEX, Yuanlin City, Taiwan) was used to close the FEP cuvette and to connect it to the glass capillary connected with the Zeiss Lightsheet Z.1 xyz stage (Blaubrand intraMark 200 μl micropipette, cat. no. 708757, BRAND GmbH & Co. KG, Wertheim am Maim, Germany) (Additional file 3: Fig. S2f). In order to pipette the samples into the cuvette, the capillary was removed. Finally, the complete FEP cuvette setup was cleaned with a detergent solution (1% Hellmanex-II in ultrapure water), sterilised in 75% ethanol for at least 2 h and washed twice with PBS.

### Specimen preparation

Organoids were cultured as described. During the splitting procedure, 20 μl of ECM containing mPO/hCCAO cell clusters were filled into the cuvette. To avoid air bubbles, the use of a 20 μl pipette tip is recommended (TipOne 10/20 μl, STARLAB, Hamburg, Germany). Subsequently, a glass capillary (Blaubrand intraMark 200 μl micropipette) that has been filled with expansion medium beforehand was connected. To ensure no leakage, the connections between the shrinking tube, the FEP cuvette, and the glass capillary were wrapped with Parafilm (Additional file 3: Fig. S2). For imaging, the FEP cuvette attached to the capillary was inserted in the Zeiss Lightsheet Z1. The imaging medium in the Zeiss Lightsheet Z1 chamber was DMEM (without phenol red) dosed with 2% penicillin and streptomycin and HEPES (1:100). During the time of observation, the medium exchange was conducted under semi-sterile conditions directly at the microscope with a 10 μl microloader tip (Microloader Tip 0.5–10 μl/2–20 μl, Eppendorf AG, Hamburg, Germany) every 2 days. The organoid density within a cuvette can be varied by using more cell organoid fragments. However, with increasing amount of cells, the frequent medium exchange should be enhanced. Due to the monolayered luminal character of epithelial organoids, only minor effects of shadowing and light scattering were observed in the conducted experiments. However with increasing cell density, it may gain importance and need to be considered.

### Image acquisition and microscopic feature extraction

Image stacks of the entire Z1-FEP-cuvette containing the mPOs/hCCAOs were acquired with the Zeiss Lightsheet Z1 microscope. The mPO cells expressed Rosa26-nTnG and were exited with a 561 nm laser. The hCCAO cells expressed H2B-eGFP and LifeAct-mCherry and were exited with a 488 nm and 561 nm laser. Both cell lines were imaged with a Carl Zeiss W Plan-Apochromat × 20/1.0 UV_VIS objective and illuminated from two sides with Zeiss LSFM × 10/0.2. During the image acquisition, the chamber was temperature- and CO_2_-controlled and constantly filled with pre-warmed DMEM containing 2% penicillin and streptomycin and HEPES (1:100). Dependent on the number of *z*-slices, views, and tiles, the size of a long-term live imaging data set recorded with LSFM is usually in the hundreds of GB up to TB range (Additional file 6: Fig. S5 and Additional file 7: Fig. S6). To handle the data, the acquired image stacks were inspected and specific regions were cropped and exported via ZEN 3.2 (Blue edition, freeware) as TIFF files per *z*-slice and time point. mPO image stacks were cropped towards the corresponding organoid (Fiji, ImageJ) and all single time points of each organoid were segmented and processed separately by using the previous published multiscale image analysis pipeline [22] with the configurations mentioned in Additional file 23: Table S1. For feature extraction and the surface approximation, the configurations mentioned in Additional file 23: Table S1 were used.

### Evaluation of segmentation performance

To evaluate the nuclei segmentation performance of the image analysis pipeline previously published [22] for live organoids, we generated ground truth (GT) data sets as a comparison to the segmentation result. For each individual organoid, a random time point was selected to manually identify the nuclei centroids of the entire organoid. A previously described custom programme [21, 22] was used to identify cell nuclei correctly detected by the segmentation (true positives, TP) and falsely detect cell nuclei (false positives, FP) and the number of cell nuclei in the GT that was not detected by the segmentation (false negatives, FN). The numbers were determined by checking, if exactly one segmented nuclei centroid was found in the spherical neighbourhood range of ten voxels of a ground truth centroid. If multiple centroids were found in the neighbourhood range, the closest was registered as TP. Based on TP, NP, and FN, the recall, precision, and F score were calculated as described in Schmitz et al. [22] (Additional file 10: Fig. S9 and Additional file 23: Table S2).

### Movement visualisation using Arivis Vision4D

3D volume rendering and 3D cell tracking was performed with Arivis Vision4D (Version: 3.1.3, Arivis AG, Munich, Germany) to visualise cell movement and rotation. Prior the use of Arivis Vision4D, the single *z*-stacks per time point were combined as hyper-stacks (Fiji/ImageJ). For the segmentation, image stacks were filtered with “Particle enhancement” (Diameter: 10, Lambda: 1). Single-cell nuclei were subsequently segmented with “Blob Finder” (Segment value: 500 μm, Threshold: 5, Watershed level: 1.303, NormalizePerFrame: true, SplitSensitivity: 82%) and tracked with “Segment Tracker” (Motion type: Brownian Motion (centroid), Max. distance: 5 μm, Track: Fusion: false – Divisoins: true, Weighting: Multiple).

### Fiji/ImageJ

Organoid and nuclei sizes for the visual inspections part of the results were measured manually on maximum intensity *z*-projections of the acquired fluorescence image data using Fiji/ImageJ.

### Mechanical 3D agent-based model

An individual cell-based model was implemented to explain the size oscillations of the pancreas-derived organoids. The mathematical model was given as a set of stochastic differential equations that were solved using the Euler-Maruyama method.

To describe the pancreas-derived organoid, we assumed it has a roughly spherical shape, with cells forming a monolayer filled with fluid at a different pressure relative to the environment. The volume of the organoid is affected by two mechanisms: (a) the influx of liquid caused by an osmotic imbalance or active pumping of the cells, and (b) cell division. While (a) is increasing the internal pressure, (b) leads to a relaxation of the surface.

Each cell was described by a small set of features, i.e. a position in 3D space and a cell size denoted by its radius. Displacement of the cells was described as a response to three forces: (1) external forces exerted by surrounding cells, given as a spring potential, (2) internal pressure of the organoid pushing the cells outwards, given by the ideal gas law, and (3) a surface bending energy, keeping the organoid in its spherical shape.

Cell division was adjusted to match the experimental data, obtained by long-term single-cell analysis of pancreas-derived organoids, but can easily be adapted to other growth dynamics.

If the average distance of neighbouring cells exceeds a certain limit, we assumed the mechanical stress to be too high and a leakage in the shell of the organoid emerges. Through the rupture, the internal liquid is released and the internal pressure decreases. Thus, the mechanical forces, exerted on the cells, might relax and the organoid deflates. When the average distances between all neighbouring cells fall below a given threshold, the shell closes and the liquid stops to be released.

For the initial model setup, we utilised the cell count data retrieved from the light sheet pipeline. To reproduce the experimental observations from the bright-field analysis pipeline, we assumed simple cell proliferation dynamics. Due to the lack of exact cell proliferation dynamics from the bright-field pipeline, we estimated the initial cell number based on the projected area of the organoid and taking into account the light sheet microscopy data. The assumed cell proliferation dynamics are fitted to the linear increase in the projected area of the organoids, retrieved from the bright-field analysis.

### Bright-field pipeline

#### Specimen preparation

For the bright-field analysis, organoids were seeded in 25 μl ECM (Matrigel, Corning, New York) droplets in suspension culture plates (48-well, Greiner Bio-One, Kremsmünster, Austria), overlaid with 250 μl expansion medium and cultured for 12 h before imaging. They were then imaged every 30 min in a 3 × 3 tile imaging (15% overlap) mode using the Zeiss Cell Observer Z.1, fully equipped with an incubation chamber and motorised stage using a Plan-Apochromat × 5/0.16 objective, with a pixel size of 1.29 μm × 1.29 μm. In total, ten planes throughout the droplet were imaged, with a *z*-distance of 50 μm (mPOs) and 65 μm (hCCAOs), respectively, capturing a *z*-range of 450 to 585 μm.

#### Image processing and organoid segmentation

Organoid growth rates were determined using a python custom-made pipeline for bright-field-based image segmentation. The whole pipeline was equipped with a general user interface. The recorded time-lapse image stacks were pre-processed with Fiji (ImageJ version 1.51n, Java version 1.8.0_6 (64-bit)) by reducing the dimensionality of the raw data set from 9 (3 × 3) tiles with 10 *z*-planes each to one stitched image with one *z*-plane per time frame using the functions Average Intensity *z*-Projection, Subtract Background (Rolling ball radius: 700 pixels, Light background, Sliding paraboloid, Disable smoothing), and Grid/Collection stitching [61] (Type: Grid: row-by-row, Order: Right & Down). The resulting image stacks were further subjected to filtering (Median, Radius: 5 pixels) and background subtraction (Rolling ball radius: 500 pixels, Light background, Sliding paraboloid), and the projected luminal areas of the organoids were using the Fiji plugin Morphological Segmentation (MorphoLibJ [62] ➔ Segmentation ➔ Morphological Segmentation; Border Image, Tolerance: 10 (mPOs), 12 (hCCAOs), Calculate dams: true, Connectivity: 6). Subsequently, the segmented images were subjected to manual editing in which incompletely segmented organoids and organoids overlaying each other were excluded. Segmented luminal areas were measured with the Fiji plugin Region Morphometry (MorphoLibJ [62] ➔ Analyse ➔ Region Morphometry). The results were plotted and statistically evaluated (Kruskal-Wallis ANOVA, *p* < 0.05) using OriginPro 2019 or Excel. For a normalisation, the projected areas were normalised to the median of the fifth time point.

#### Mesoscopic feature extraction

Quantitative features were extracted using a Python script and were defined as follows: A size oscillation event consists of a decline phase followed by an expansion phase. The start of a decline phase was defined as the time point after which the area declines by 5%, and the end is marked if the area increases again. Expansion phases were defined between the end of a decline phase and the start of the following decline phase. As additional criterion, the duration of expansion phases is greater than or equal to five time points, and the correlation coefficient of the fitted polynomial is above 0.9. The number of decline and expansion phases per organoid was determined including their duration and slope. Subsequently, maximum and average expansion slopes were computed. The average expansion factor is specified as the average slope of all detected expansion phases per organoid. The maximum expansion factor is specified as the maximum slope of all detected expansion phases per organoid. Outliers in average expansion were defined as smaller than the first quartile minus 1.5 × IQR or above the third quartile plus 1.5 × IQR. The circularity was monitored continuously and is defined as 4π(area/perimeter^2^). Its standard deviation is displayed as the average standard deviation in all analysed wells. Organoids displaying a circularity below 0.6 were considered as deficiently segmented and were excluded from further analyses. Due to deficient segmentation during organoid formation, the projected area was normalised to the fifth time point of acquisition.

## Supplementary Information

**Additional file 1:** Definitions. Definitions of the terminology used in this work and list of abbreviations.

**Additional file 2:** **Fig. S1.** Light sheet and bright field time-resolved observation allow quantitative analyses of micro-, meso- and macroscale dynamics. Using a light sheet-based fluorescent microscope time-resolved image stack of organoids are recorded. The high-resolution images are subjected to nuclei segmentation [22] for the quantification of dynamics on single cell level (microscale). Besides that, dynamics, such as size oscillation events, of individual organoids (mesoscale) can be analysed. The restricted throughput of this pipeline is matched with the analyses based on time-resolved bright field images. Here, the dynamics of high numbers of organoids are quantified based on the normalised (norm.) projected (proj.) luminal areas. The pipeline also enables the observation of entire organoid cultures (macroscale) within individual wells.

**Additional file 3:** **Fig. S2.** Ultra-thin FEP-foil cuvette holders for live recordings with the Zeiss Lightsheet Z.1 microscope system. (a) Illustration of the general setup of the Zeiss Lightsheet Z.1 microscope. (b) Close-up of the microscope chamber with the downwards directed Z1-FEP-cuvette enclosing the sample. (c) Close-up of the sample holder. The shrinking tube that seals the FEP cuvette and connects it with the glass capillary is depicted in black. (d) CAD-derived drawings of positive moulds of the FEP cuvette and the glass capillary needed to produce the Z1-FEP-cuvette. (e) Printed mould with a glass capillary used to form the Z1-FEP-cuvette in the vacuum forming process. (f) Ready-to-use Z1-FEP-cuvette. (g) mPOs grown for 7 days in the Z1-FEP-cuvette.

**Additional file 4:** **Fig. S3.** Validation of the temperature properties of the Zeiss Lightsheet Z.1 microscope. (a) Illustration of the temperature distribution inside of the Zeiss Lightsheet Z.1 microscope chamber and the corresponding measurement landmarks. Beside the open, upper part with a slightly lower value, the temperature is equally distributed throughout the chamber. (b) Results of the measurement of the heating-up time. The included heating unit of the microscope needs to heat up the medium starting from room temperature (21 °C). After 12 min the medium reaches the physiological temperature of 37 °C.

**Additional file 5:** **Fig. S4.** Validation of the pH properties of the Zeiss Lightsheet Z.1 microscope. (a) Illustration of the pH-value distribution inside the chamber of the Zeiss Lightsheet Z.1 microscope and the corresponding measurement landmarks. After filling the chamber with buffered media, the pH-value is evenly distributed at 7.5 throughout the chamber. (b) The constant CO_2_ fumigation that is directed over the liquid column is not able to recover a lower pH-value over time. The pH-value of the medium changes from 8.5 to 8 but it never reaches the physiologically necessary 7.5 (liquid depth: 3 cm). The same is observed at 1 cm and 2 cm liquid depth. At the bottom of the chamber, the pH-value does not change within 48 h. (c) Once the inserted medium has the right pH-value, the incubation system is able to keep it on the same level for more than 2 days.

**Additional file 6:** **Fig. S5.** Overview of entire hCCAO cultures within one Z1-FEP-cuvette and observation of isolated single-cell dynamics. hCCAOs expressed the nuclei marker H2B-eGFP (magenta) and the F-actin cytoskeletal marker LifeAct-mCherry (green). (a) Maximum intensity z-projection of the entire field of view in the Lightsheet Z1 microscope. One cuvette (i) with low organoid density and one cuvette (ii) with high organoid density are displayed. We counted about 120 organoids in the cuvette (ii) with high organoid density. Organoids show different sizes and isolated cell nuclei are visible in the interspaces. Scale bar: 250 μm. (b) Excerpts of the maximum intensity z-projections shown in (a). Isolated single organoid cells show signs of polarisation and undergo cell division. Scale bars: Cell division, Polarisation - 10 μm, Formation – 20 μm. Microscope: Zeiss Lightsheet Z.1; objective lenses: detection: W Plan-Apochromat 20x/1.0, illumination: Zeiss LSFM 10x/0.2; laser lines: 488 nm, 561 nm; filters: laser block filter (LBF) 405/488/561; voxel size: 1.02 × 1.02 × 2.00 μm^3^; recording interval: 30 min.

**Additional file 7:** **Fig. S6.** Representative overview images of three different mPO cultures grown in Z.1-FEP-cuvettes. (a) mPO grown within the Z.1-FEP-cuvette were kept in an incubator as a control for organoids grown within the Z.1 microscope. Images were taken directly after seeding, after 6 days and 10 days. (b) Two representative mPO cultures expressing the nuclei marker Rosa26-nTnG (grey) were imaged with the Zeiss Z.1 microscope over 6 days. Dependent on the number of views, tiles, z-planes and the temporal resolution, the amount of data which is generated and needs to be processed varies between hundreds of gigabyte and tens of terabyte (cuvette 1: total acquisition time: 6 days, temporal resolution: 30 min, views: 1, tiles: 1, total size: 220 GB; cuvette 2: total acquisition time: 6 days, temporal resolution: 30 min, views: 4 (only one is shown), tiles: 1, total size: 1054 GB). Microscope: (a) Zeiss Axio Imager, (b) Zeiss Lightsheet Z.1; objective lenses: (a) Plan S 1.0x FWD 81 mm, (b) detection: W Plan-Apochromat 20x/1.0, illumination: Zeiss LSFM 10x/0.2; laser lines: 561 nm; filters: laser block filter (LBF) 405/488/561; voxel size: 1.02 × 1.02 × 2.00 μm^3^, recording interval: 30 min; scale bar: 100 μm.

**Additional file 8:** **Fig. S7.** Dynamic processes observed in organoid morphogenesis. hCCAOs expressed the nuclei marker H2B-eGFP (magenta) and the F-actin cytoskeletal marker LifeAct-mCherry (green) and were cultured in Z1-FEP-cuvettes for long-term live observation. The figure shows excerpts of maximum intensity z-projections. Manual tracking of cell nuclei was performed using the *Manual Tracking* plugin in *Fiji*. Microscope: Zeiss Lightsheet Z.1; objective lenses: detection: W Plan-Apochromat 20x/1.0, illumination: Zeiss LSFM 10x/0.2; laser lines: 488 nm, 561 nm; filters: laser block filter (LBF) 405/488/561; voxel size: 1.02 × 1.02 × 2.00 μm^3^; recording interval: 30 min; scale bars: 50 μm, 25 μm (inset).

**Additional file 9:** **Fig. S8.** Time-resolved, representative images of the growth and heterogeneity in mPOs. Illustrated are single organoids extracted from three different cultures showing rotation, size oscillation, luminal dynamics, fusion and different cell nucleus sizes. In addition, cell division events at a late stage (day 4, 5, 6) and the formation of differently sized organoid fragments are shown. Besides the luminal dynamics, which are imaged by the use of a bright field microscope, all behaviour patterns and appearances were observed from organoids expressing the nuclei marker Rosa26-nTnG (grey) and grown within the Z.1-FEP-cuvette inside the Z.1 Lightsheet microscope. The red rectangles indicate the corresponding close-up, the red arrows indicate the position within the organoid where the event occurs, the curved arrow indicate the direction of rotation and the red circle indicates the volume change during a size oscillation event. Microscope fluorescence images: Zeiss Lightsheet Z.1; Plan S 1.0x FWD 81 mm, detection: W Plan-Apochromat 20x/1.0, illumination: Zeiss LSFM 10x/0.2; laser lines: 561 nm; filters: laser block filter (LBF) 405/488/561; voxel size: 1.02 × 1.02 × 2.00 μm^3^; recording interval: 30 min; Microscope bright field images: Zeiss Axio Observer Z.1; objective lenses: Plan-Apochromat 5x/0.16, voxel size: 1.26 × 1.26 × 4 μm3, avg. z-projection.

**Additional file 10:** **Fig. S9.** Visualisation of segmentation performance in live mPOs expressing nuclear tdTomato. a) Maximum intensity z-projections of raw image stacks of three different mPOs. Image quality ensures a clear separation amongst the labelled nuclei, which is essential for semi-automated nuclei segmentation. Different colours indicate individual nuclei in overviews and close-ups of segmented nuclei. Microscope: Zeiss Lightsheet Z.1; objective lenses: detection: W Plan-Apochromat 20x/1.0, illumination: Zeiss LSFM 10x/0.2; laser lines: 561 nm; filters: laser block filter (LBF) 405/488/561; voxel size: 1.02 × 1.02 × 2.00 μm3; recording interval: 30 min, scale bar: 100 μm b) Evaluation of segmentation performance for different organoids. The performance was measured against a manually determined ground truth for the organoid I (red), II (blue) and III (green). The performance metrics recall, precision and F score are determined based on the number of true positives, false negatives and false positives (Supplementary Table 2). TP: true positives; FN: false negatives; FP: false negatives.

**Additional file 11:** **Fig. S10.** Illustration of the input, the assumptions and the output of the model. Measured cell counts and cell division dynamics are used to initialise the simulations. Organoid behaviour is based on the following main assumptions [1]. Each cell produces a substance with constant rate Jin, the substance leads to increase of internal pressure [2]. Cell displacement is driven by mechanical cell-cell-interactions, internal pressure and a surface energy of the organoid [3]. If the organoid shell ruptures, substance is released to the outside with flux Jout, releasing pressure and leading to a contraction of the sphere until the cell-cell connections are restored. The output of the model is the volume data as a function of time of the simulated organoids.

**Additional file 12:** **Fig. S11.** Analysis of simulated monocystic mPOs. Simulations starting with heterogenous initial cell numbers confirm an influence of organoid size onto the number of oscillation events. The colour code signals the number of registered size oscillation events. The amount of size oscillations is dependent on the initial size of the organoids. While small organoids tend to show frequent inflation-deflation oscillations, initially larger organoids seem less prone to oscillation events.

**Additional file 13:** **Fig. S12.** mPO feature extraction using the bright field analysis pipeline. (a) The initial and final projected luminal areas correlate positively in healthy mPOs (R^2^-value = 0.7445). (b) The maximum slope of the expansion phases are in average higher than the average slope. (c) The minimum area falls in average slightly below the initial area. (d) Furthermore, the final area equals the maximum area, which indicates continuous growth – green: linear trend line, m: slope, red: f(x) = 1x. (e) Average circularity over time of organoids grown in three wells. Average standard deviation estimated within the three wells is indicated. Mathematically possible values range between 0 and 1.

**Additional file 14:** **Fig. S13.** Bright field pipeline allows detailed analysis of polycystic hCCAOs. (a) Polycystic hCCAOs display a dense phenotype. Microscope: Zeiss Axio Observer Z.1; objective lenses: Plan-Apochromat 5x/0.16, avg. z-projection, voxel size: 1.29 × 1.29 × 65 μm^3^, scale bar overview: 500 μm, close-up: 25 μm. (b) The average circularity is around 0.8 over time. (c) Similarly to monocystic organoid cultures, the detected projected luminal areas and growth behaviours are heterogeneous. Box plot analysis, median in green (*n* = 87) (d) While the median projected luminal areas of three different wells (technical replicates) vary, the normalised projected area increase is similar (n = 87, 34, 63). (e) The initial projected area correlates with the final projected area (R^2^ = 0.8744) with a linear regression slope m = 1.5857. (f) The organoids display a similar average expansion factor independent of their initial size with an average of 0.02 and outliers (red) lying above 0.078.

**Additional file 23:** **Table S1.** Used settings for the segmentation and post-processing of the data obtained with the light-sheet pipeline. **Table S2**. Evaluation of segmentation performance for different organoids. The performance was measured against a manually determined ground truth for organoid I (red), II (blue) and III (green). The performance metrics recall, precision and F score are determined from the number of true positives, false negatives and false positives. Values range from 0 (worst performance) to 1 (optimal performance). GT: number of cell nuclei in the ground truth; SC: number of cell nuclei determined by segmentation; TP: true positives; FN: false negatives; FP: false negatives.

**Additional file 24:** Supplementary Theoretical Considerations. A mechanical model to describe the dynamics of pancreas organoids.

## Data Availability

The light sheet image datasets generated during and/or analysed during the current study are available in the Zenodo repository, (doi: 10.5281/zenodo.4419985) [63]. The bright-field analysis pipeline is available in the GitHub repository (https://github.com/physical-biology-group/Three-dimensional-cell-biology.git) [64]. Additional raw microscopy data can be obtained from the corresponding author (fpampalo@bio.uni-frankfurt.de).
