# Supplementary material for: Long-term live imaging and multiscale analysis identify heterogeneity and core principles of epithelial organoid morphogenesis
Source: BMC Biol. 2021 Feb 24;19:37. doi: 10.1186/s12915-021-00958-w (PMC7903752; doi:10.1186/s12915-021-00958-w)
Supplement: Supplementary file 9 — Additional file 1: Definitions. Definitions of the terminology used in this work and list of abbreviations. [file 12915_2021_958_MOESM1_ESM.pdf]

Definitions

- **Agent-based model:** A computational model, in which cells are represented as autonomous decision-making agents. Agents interact with their environment based on a given ruleset. Agent-based models allow for a high level of physical detail and can reproduce complex behaviour patterns.
- **Biological replicate:** An independently performed experiment with either a new organoid line or a complementary passage of the same organoid line.
- **Core regulatory principles:** Mechanistic understanding of multicellular systems (Sasai *et al.*, 2013).
- **hCCAO:** Human cholangiocarcinoma-derived organoids.
- **mPO:** Murine pancreas-derived organoids.
- **Monocystic:** Spherical mono-layered epithelium with a single lumen.
- **Multiscale organoid analysis:** The analysis of an entire organoid culture at the three levels of observation:
  - **Microscale**: Single cell
  - **Mesoscale**: Individual organoid
  - **Macroscale**: Entire organoid culture
- **Non-invasive imaging:** Organoids are imaged without the use of any additional fluorescent dye, any additional substance or any chemical-physical influence besides the medium exchange to ensure optimal growth conditions.
- **Organoid degeneration:** Overall shrinking of the organoid accompanied by nuclear condensation and fading nuclei signals.
- **Organoid luminal dynamics:** Formation, retraction and rupture of duct-like structures within the lumen of an organoid.
- **Organoid size:** The term "size" refers to an organoid's volume [voxels] based on surface approximations derived from the light sheet image data or to the organoid's projected luminal area [mm^2] based on segmented equatorial planes derived from the bright field image data.
- **Polycystic:** An irregularly shaped multi-layered epithelium surrounding multiple lumens.
- **Projected luminal area:** The segmented and measured equatorial plane of an organoid.
- **Size oscillation event:** Size alteration of an epithelial organoid characterised by the following phases:
  - **Decline phase:** Starts with the time point, upon which the volume/area is 5% smaller than at the previous time point. The decline phase ends when volume/area increases again.
  - **Expansion phase:** Starts with the time point at the end of a decline phase and ends the start of the following decline phase if this phase comprises more than five time points.
- **Size-oscillation frequency:** The rate with which size oscillation events occur.
- **Technical replicate:** One well of organoids that contains several individual organoids of identical origin.
- **Z1-FEP-cuvette:** Custom sample holder for live imaging with the Zeiss Lightsheet Z.1 microscope system based on the previously described ultra-thin FEP-foil cuvette (Hötte *et al.*, 2019).
