# Supplementary material for: Long-term live imaging and multiscale analysis identify heterogeneity and core principles of epithelial organoid morphogenesis
Source: BMC Biol. 2021 Feb 24;19:37. doi: 10.1186/s12915-021-00958-w (PMC7903752; doi:10.1186/s12915-021-00958-w)
Supplement: Supplementary file 10 — Additional file 2: Fig. S1. Light sheet and bright field time-resolved observation allow quantitative analyses of micro-, meso- and macroscale dynamics. Using a light sheet-based fluorescent microscope time-resolved image stack of organoids are recorded. The high-resolution images are subjected to nuclei segmentation [22] for the quantification of dynamics on single cell level (microscale). Besides that, dynamics, such as size oscillation events, of individual organoids (mesoscale) can be analysed. The restricted throughput of this pipeline is matched with the analyses based on time-resolved bright field images. Here, the dynamics of high numbers of organoids are quantified based on the normalised (norm.) projected (proj.) luminal areas. The pipeline also enables the observation of entire organoid cultures (macroscale) within individual wells. [file 12915_2021_958_MOESM2_ESM.pdf]

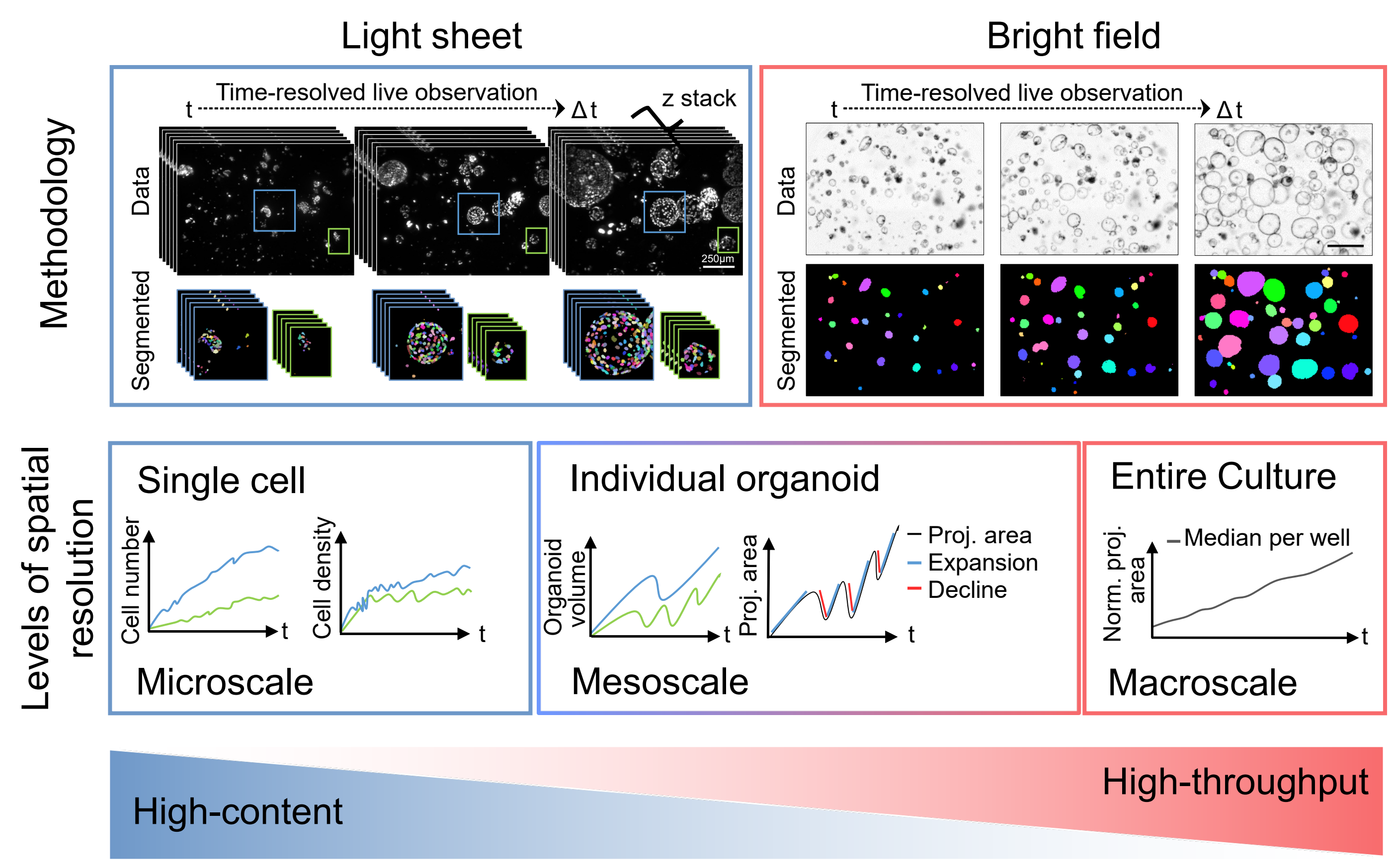

Light sheet
Bright field
Methodology
Time-resolved live observation
t
Δt
z stack
Data
Segmented
250μm
Levels of spatial resolution
Single cell
Cell number
Cell density
t
Microscale
Individual organoid
Organoid volume
Proj. area
Proj. area
Expansion
Decline
Mesoscale
Entire Culture
Norm. proj. area
Median per well
Macroscale
High-content
High-throughput
