# Supplementary figures and images for: Long-term live imaging and multiscale analysis identify heterogeneity and core principles of epithelial organoid morphogenesis
Source: BMC Biol. 2021 Feb 24;19:37. doi: 10.1186/s12915-021-00958-w (PMC7903752; doi:10.1186/s12915-021-00958-w)

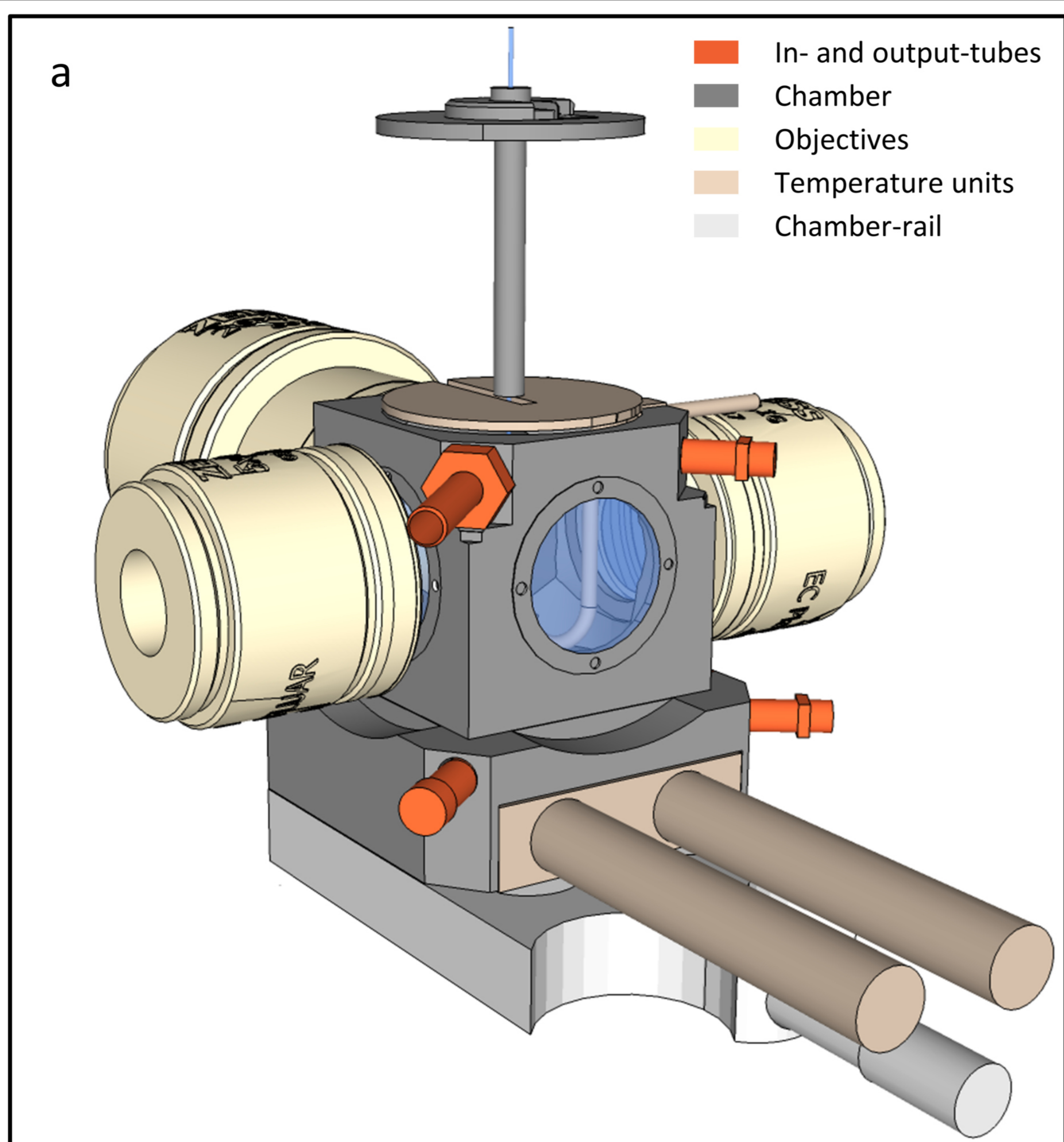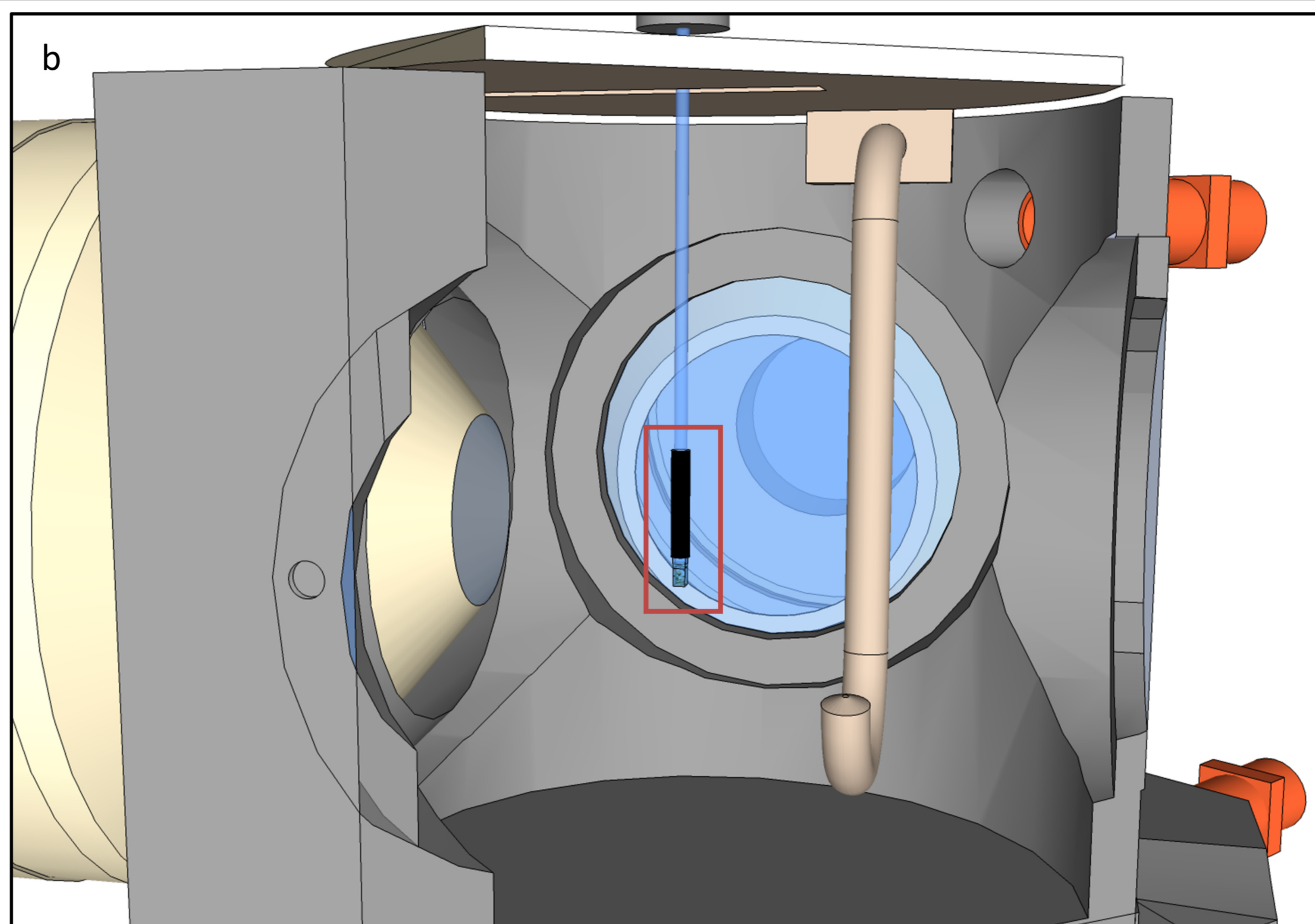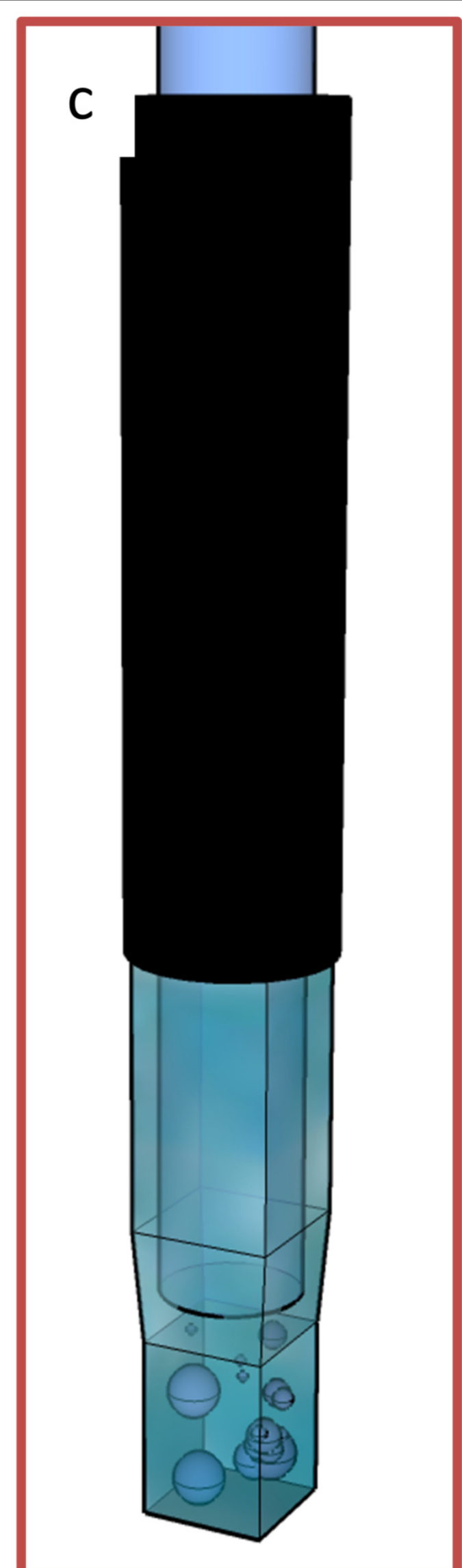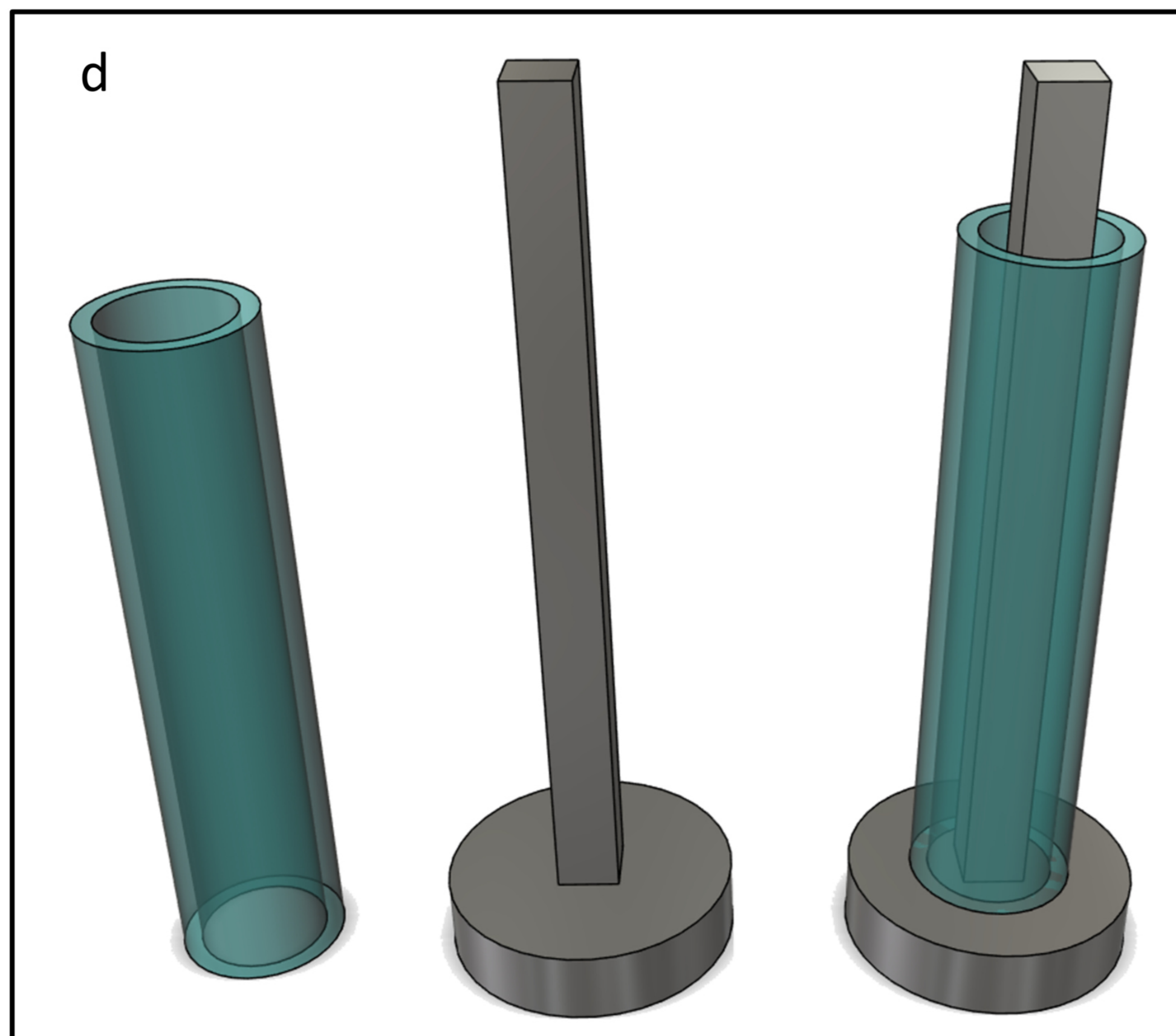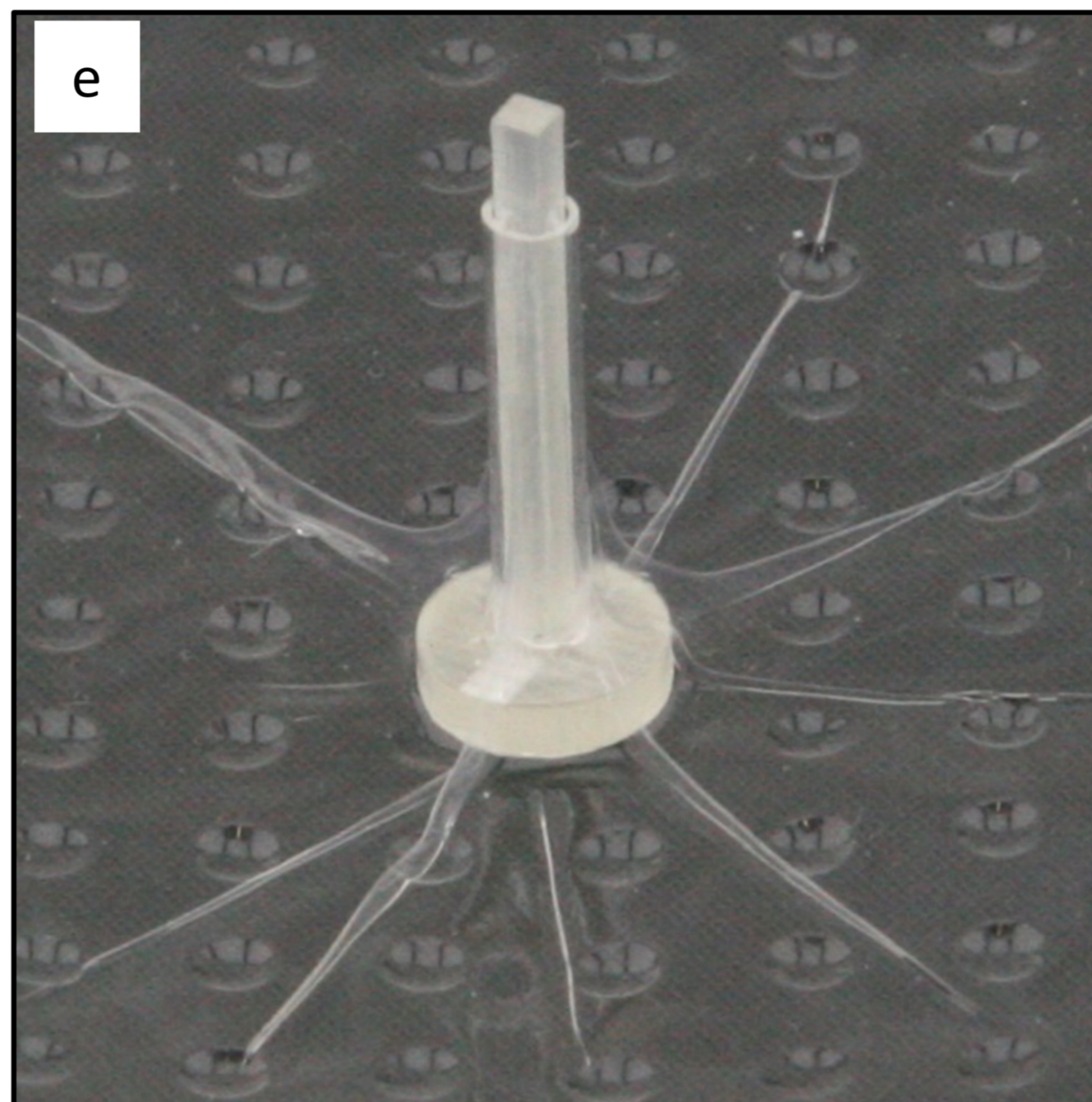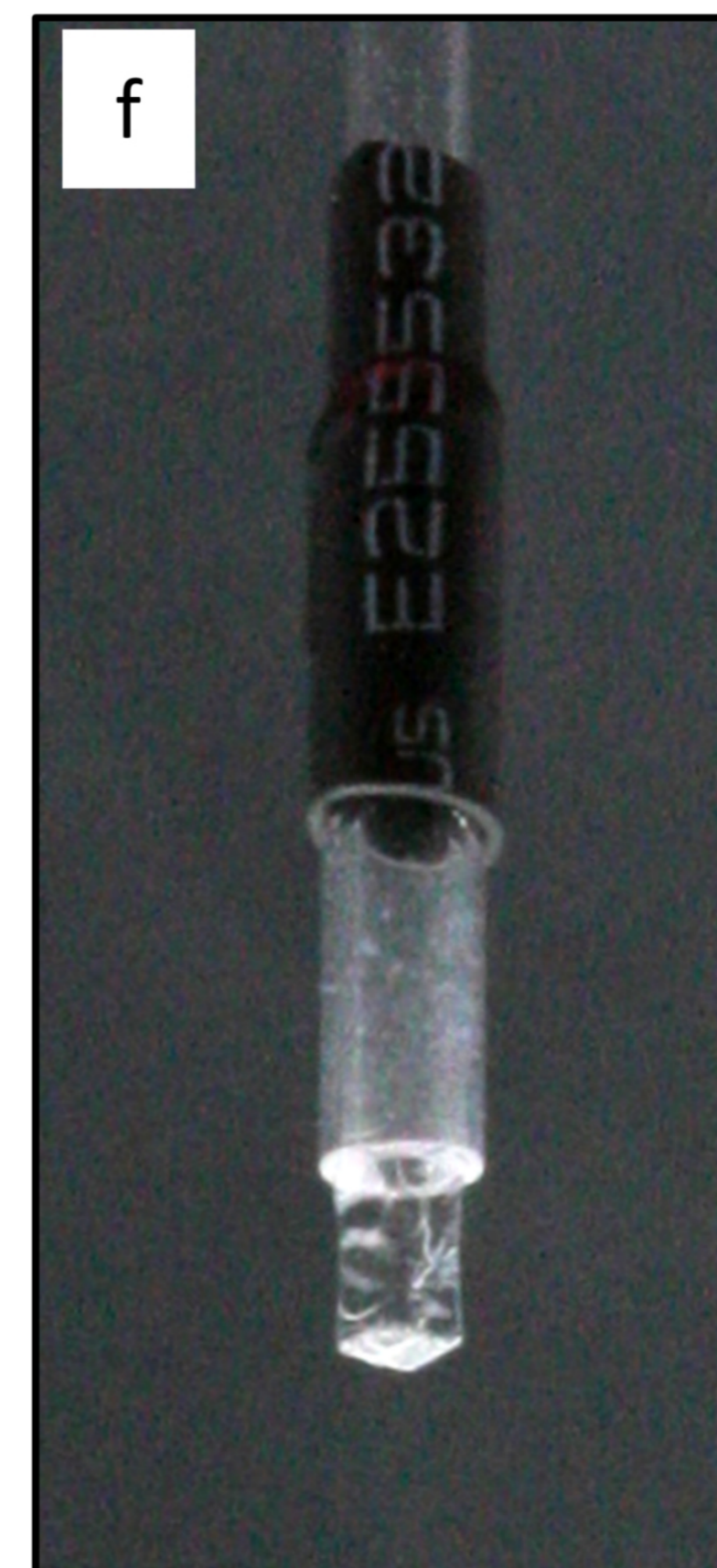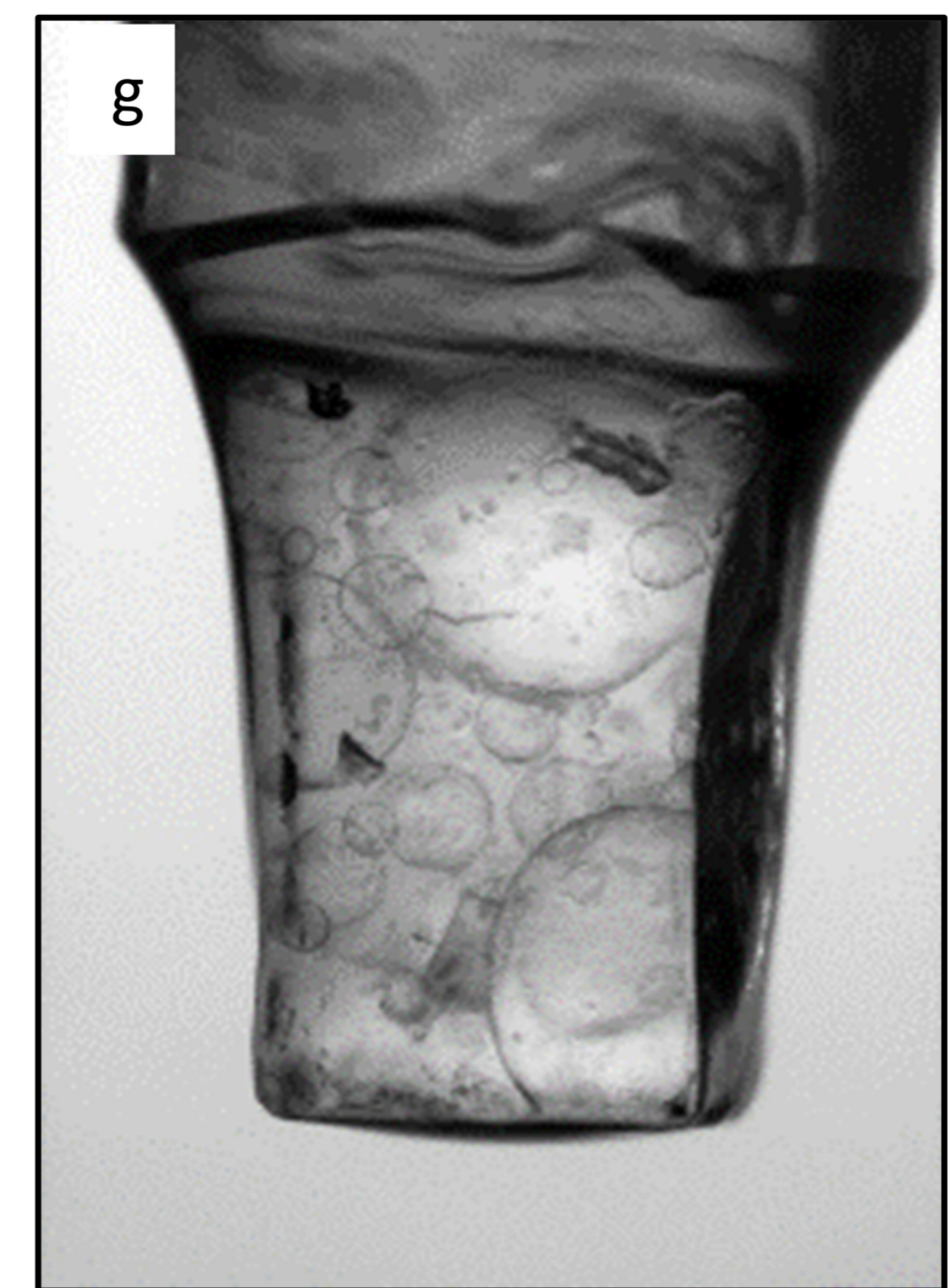

Supplement: Supplementary file 11 — Additional file 3: Fig. S2. Ultra-thin FEP-foil cuvette holders for live recordings with the Zeiss Lightsheet Z.1 microscope system. (a) Illustration of the general setup of the Zeiss Lightsheet Z.1 microscope. (b) Close-up of the microscope chamber with the downwards directed Z1-FEP-cuvette enclosing the sample. (c) Close-up of the sample holder. The shrinking tube that seals the FEP cuvette and connects it with the glass capillary is depicted in black. (d) CAD-derived drawings of positive moulds of the FEP cuvette and the glass capillary needed to produce the Z1-FEP-cuvette. (e) Printed mould with a glass capillary used to form the Z1-FEP-cuvette in the vacuum forming process. (f) Ready-to-use Z1-FEP-cuvette. (g) mPOs grown for 7 days in the Z1-FEP-cuvette. [file 12915_2021_958_MOESM3_ESM.pdf]

a

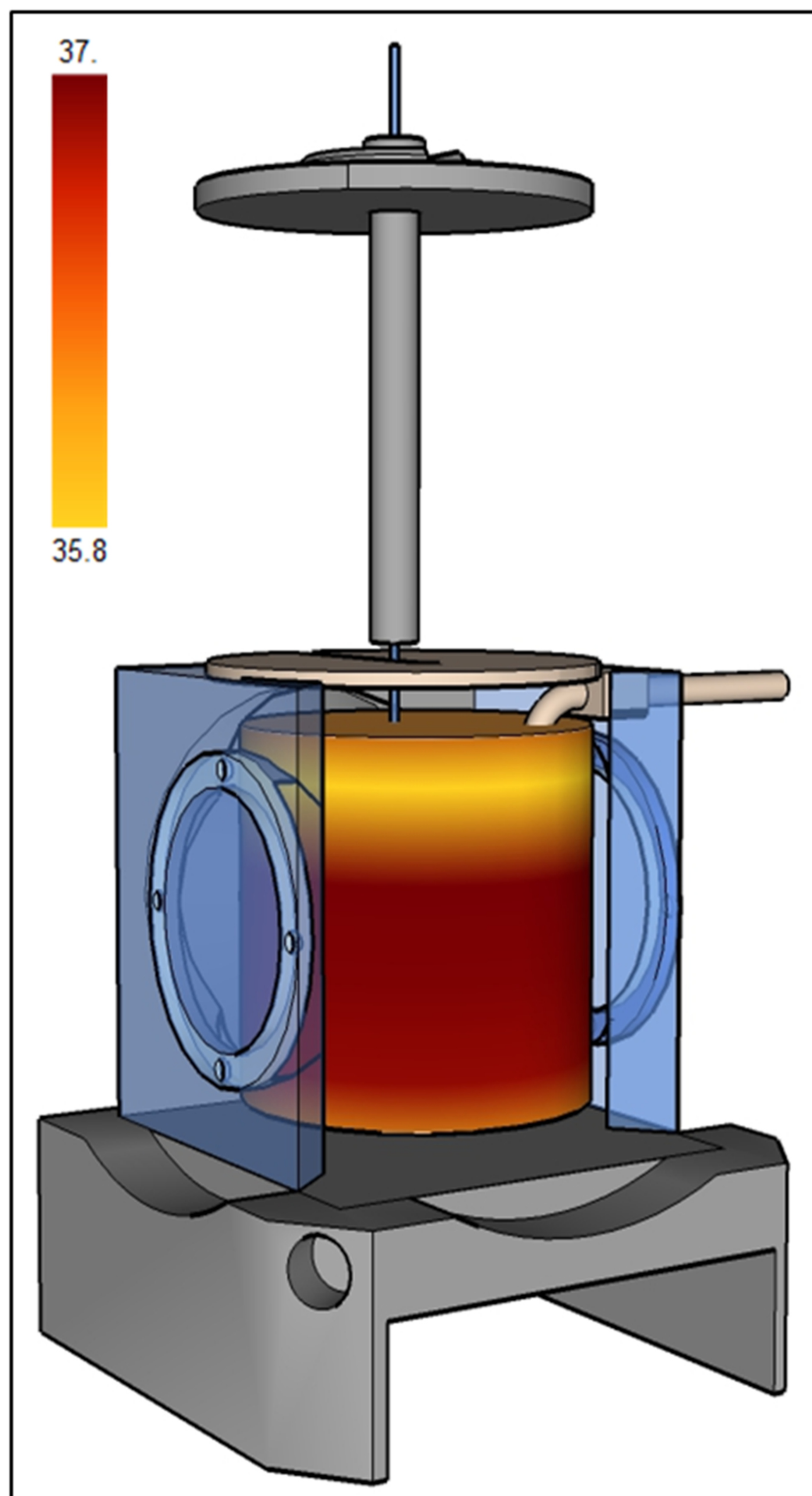

Temperature - landmarks

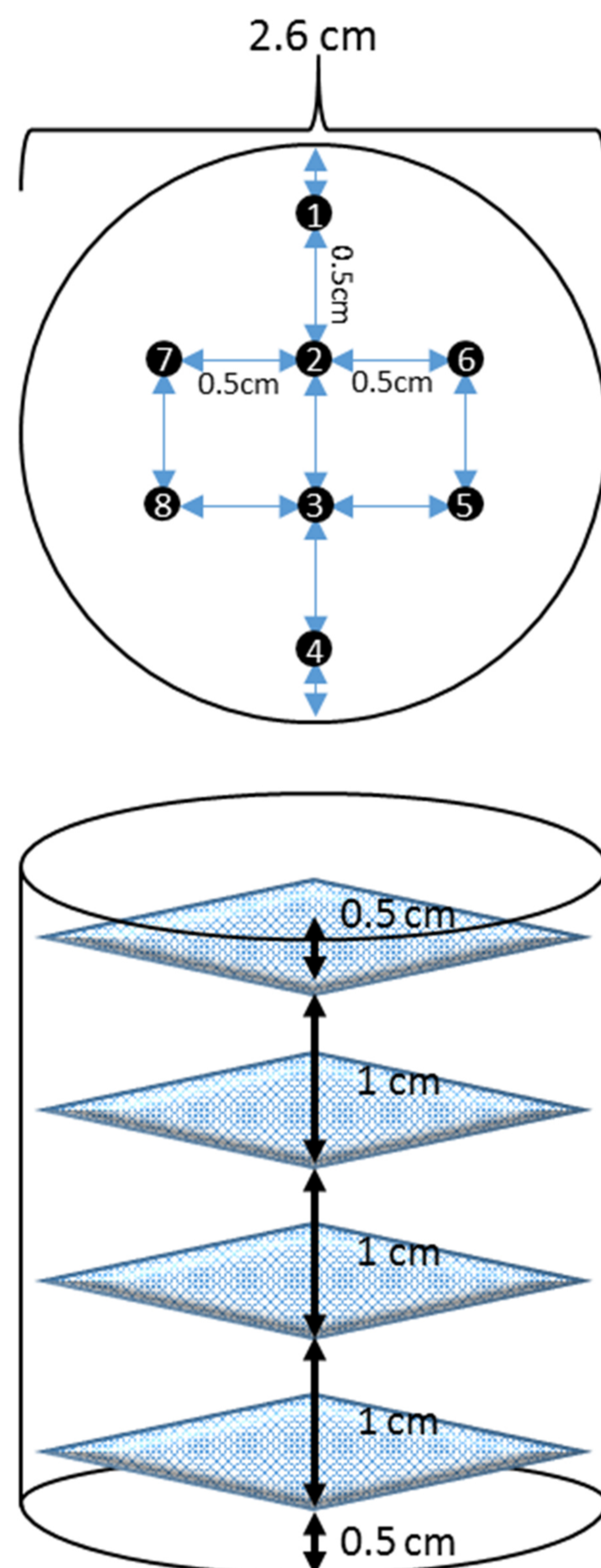

b

Heating-up time

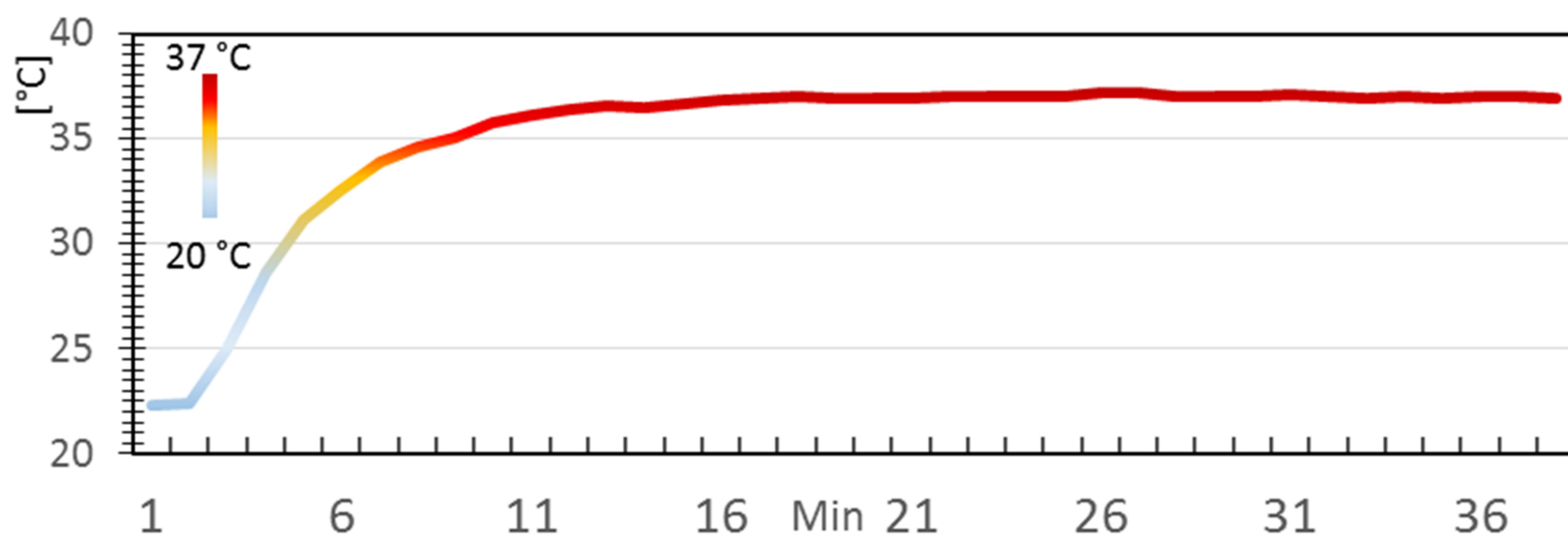

Supplement: Supplementary file 12 — Additional file 4: Fig. S3. Validation of the temperature properties of the Zeiss Lightsheet Z.1 microscope. (a) Illustration of the temperature distribution inside of the Zeiss Lightsheet Z.1 microscope chamber and the corresponding measurement landmarks. Beside the open, upper part with a slightly lower value, the temperature is equally distributed throughout the chamber. (b) Results of the measurement of the heating-up time. The included heating unit of the microscope needs to heat up the medium starting from room temperature (21 °C). After 12 min the medium reaches the physiological temperature of 37 °C. [file 12915_2021_958_MOESM4_ESM.pdf]

a

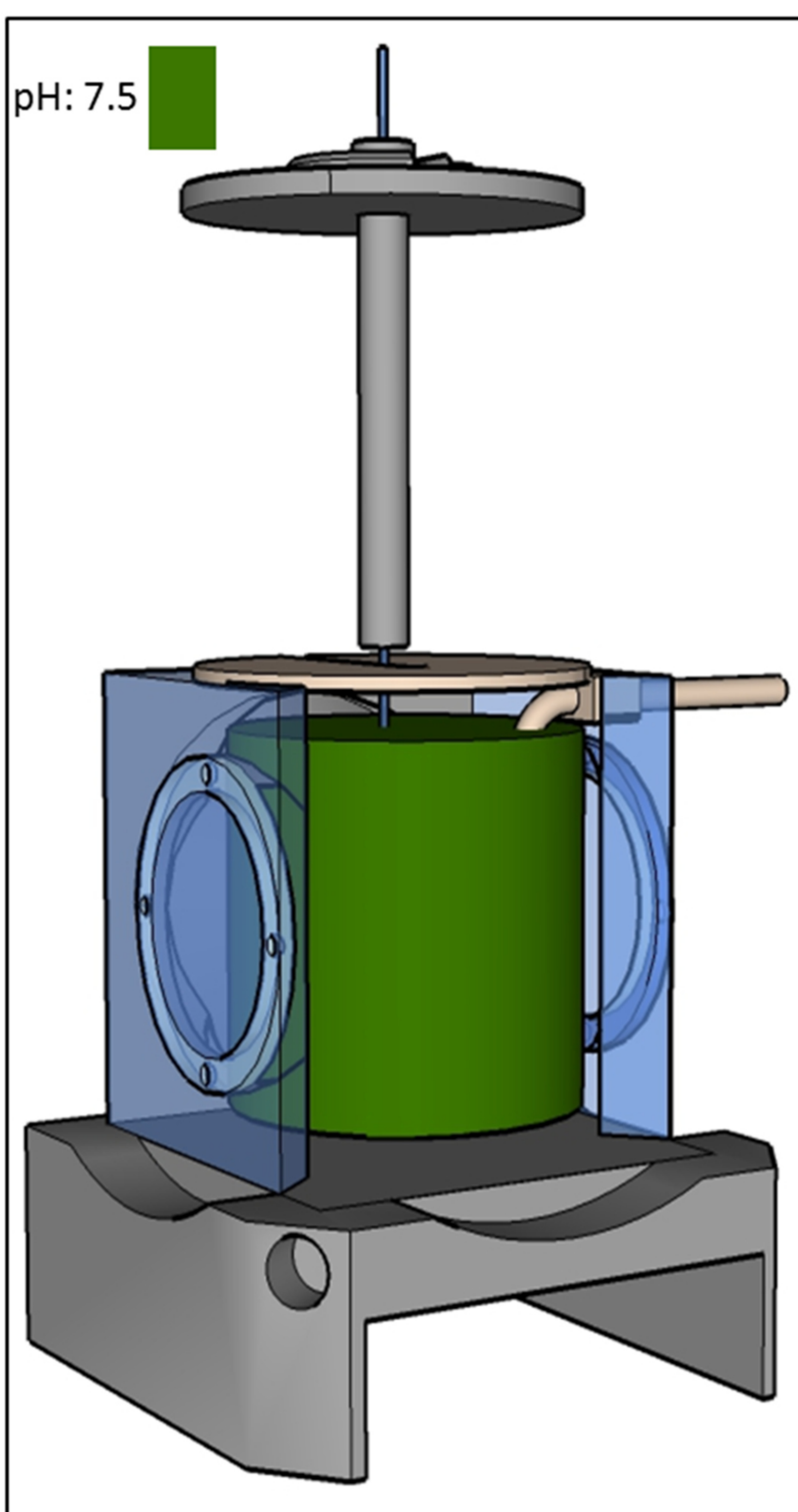

pH - landmarks

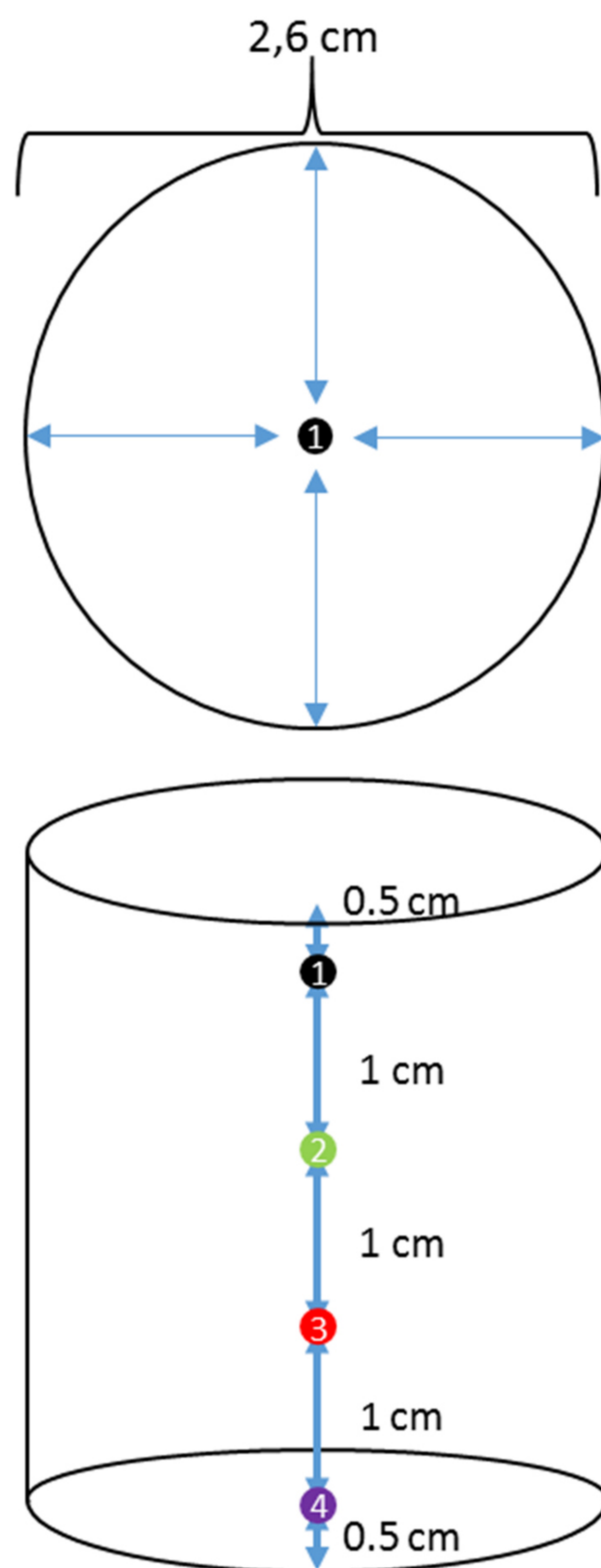

b

pH-value recovery potential over time

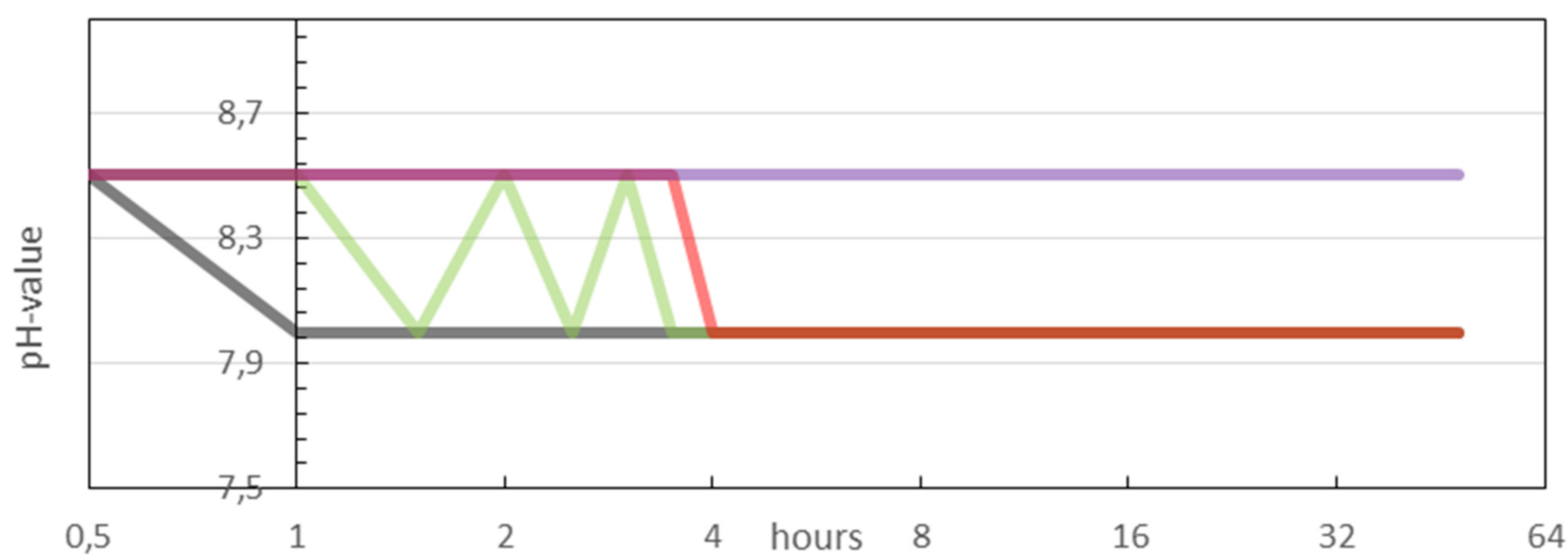

c

Average pH-value over time

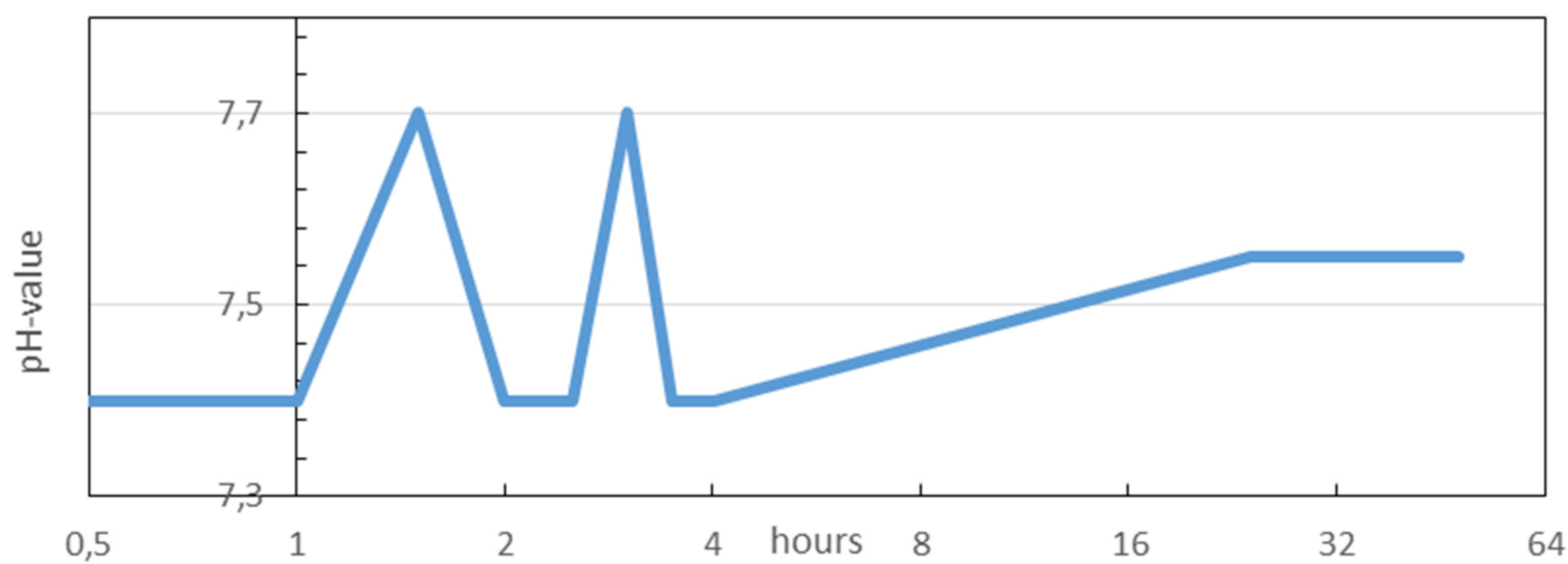

Supplement: Supplementary file 13 — Additional file 5: Fig. S4. Validation of the pH properties of the Zeiss Lightsheet Z.1 microscope. (a) Illustration of the pH-value distribution inside the chamber of the Zeiss Lightsheet Z.1 microscope and the corresponding measurement landmarks. After filling the chamber with buffered media, the pH-value is evenly distributed at 7.5 throughout the chamber. (b) The constant CO2 fumigation that is directed over the liquid column is not able to recover a lower pH-value over time. The pH-value of the medium changes from 8.5 to 8 but it never reaches the physiologically necessary 7.5 (liquid depth: 3 cm). The same is observed at 1 cm and 2 cm liquid depth. At the bottom of the chamber, the pH-value does not change within 48 h. (c) Once the inserted medium has the right pH-value, the incubation system is able to keep it on the same level for more than 2 days. [file 12915_2021_958_MOESM5_ESM.pdf]

Time-resolved live observation

t ----->  $\Delta t$

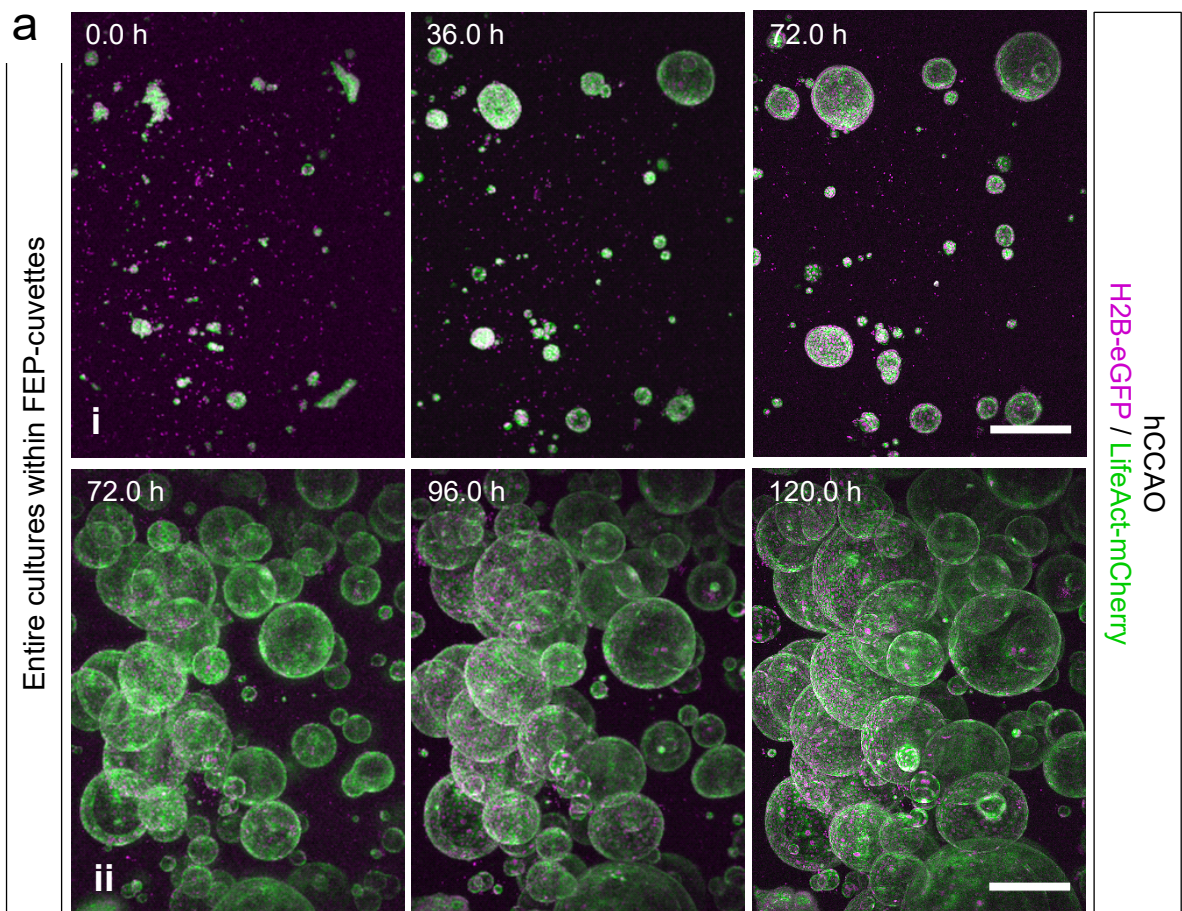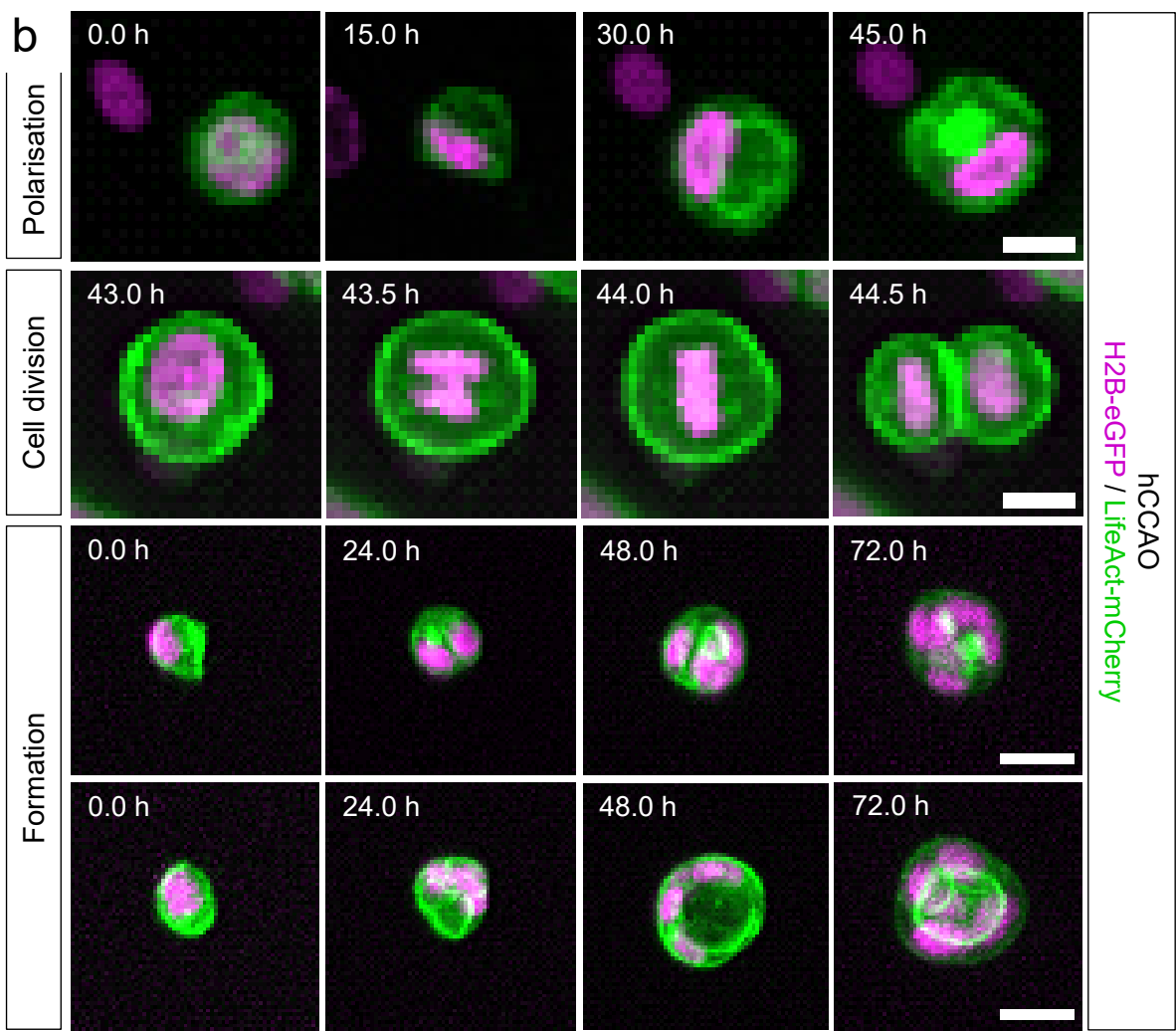

Supplement: Supplementary file 14 — Additional file 6: Fig. S5. Overview of entire hCCAO cultures within one Z1-FEP-cuvette and observation of isolated single-cell dynamics. hCCAOs expressed the nuclei marker H2B-eGFP (magenta) and the F-actin cytoskeletal marker LifeAct-mCherry (green). (a) Maximum intensity z-projection of the entire field of view in the Lightsheet Z1 microscope. One cuvette (i) with low organoid density and one cuvette (ii) with high organoid density are displayed. We counted about 120 organoids in the cuvette (ii) with high organoid density. Organoids show different sizes and isolated cell nuclei are visible in the interspaces. Scale bar: 250 μm. (b) Excerpts of the maximum intensity z-projections shown in (a). Isolated single organoid cells show signs of polarisation and undergo cell division. Scale bars: Cell division, Polarisation - 10 μm, Formation – 20 μm. Microscope: Zeiss Lightsheet Z.1; objective lenses: detection: W Plan-Apochromat 20x/1.0, illumination: Zeiss LSFM 10x/0.2; laser lines: 488 nm, 561 nm; filters: laser block filter (LBF) 405/488/561; voxel size: 1.02 × 1.02 × 2.00 μm3; recording interval: 30 min. [file 12915_2021_958_MOESM6_ESM.pdf]

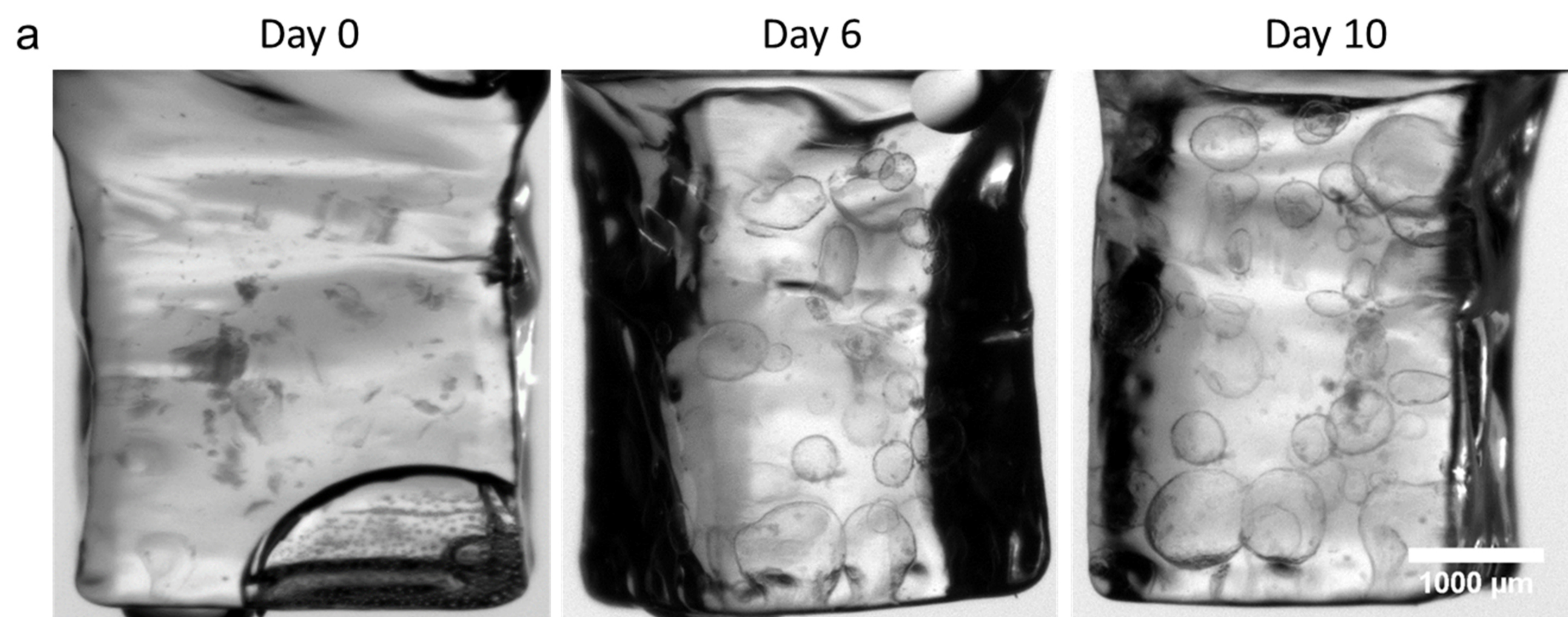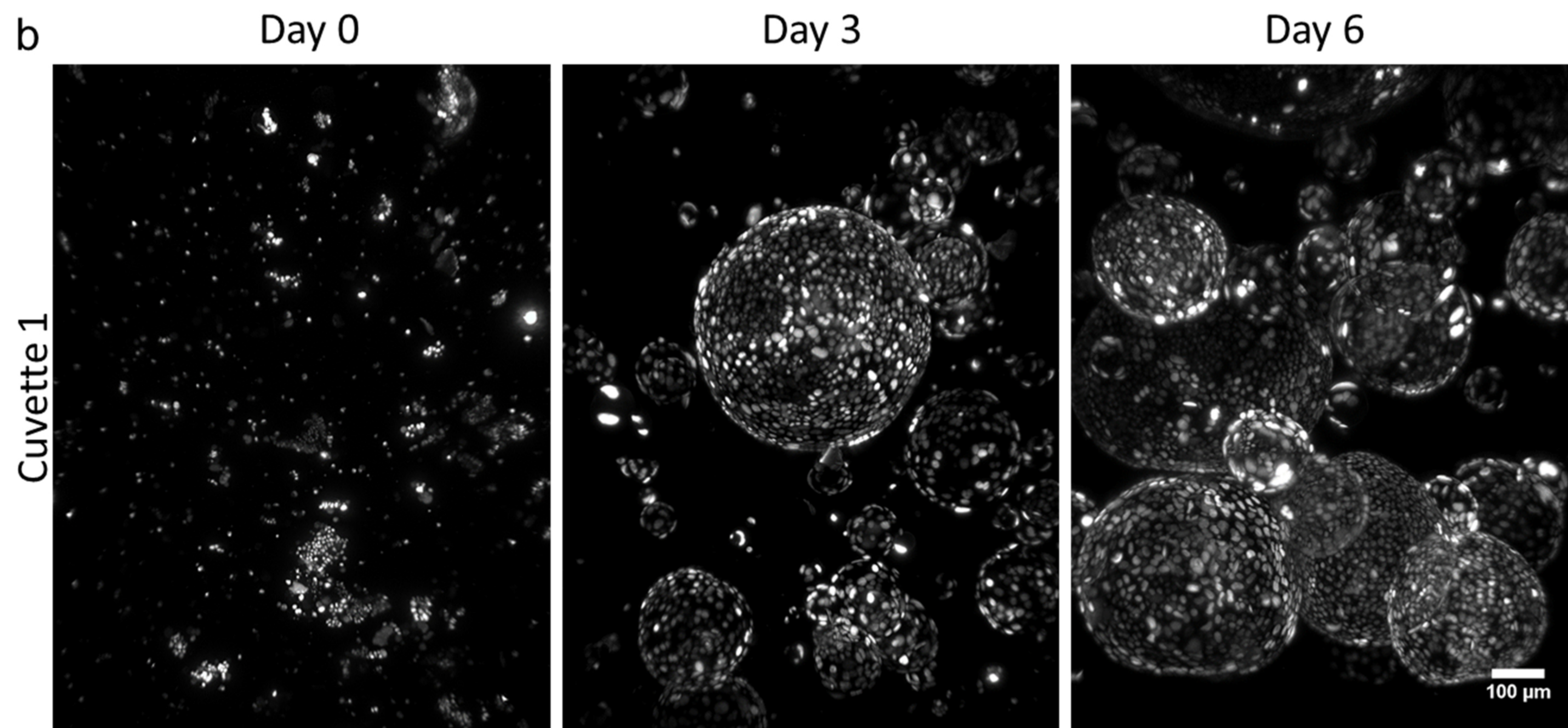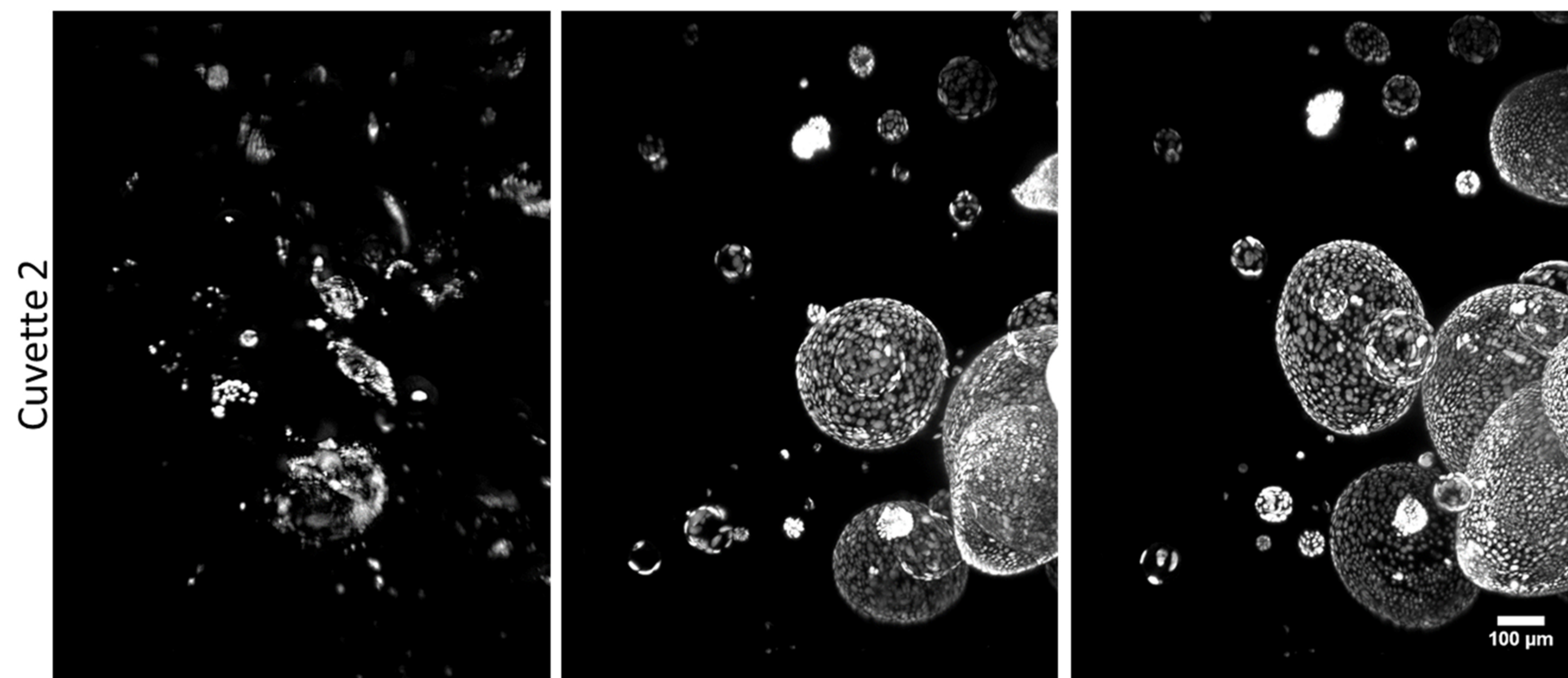

Supplement: Supplementary file 15 — Additional file 7: Fig. S6. Representative overview images of three different mPO cultures grown in Z.1-FEP-cuvettes. (a) mPO grown within the Z.1-FEP-cuvette were kept in an incubator as a control for organoids grown within the Z.1 microscope. Images were taken directly after seeding, after 6 days and 10 days. (b) Two representative mPO cultures expressing the nuclei marker Rosa26-nTnG (grey) were imaged with the Zeiss Z.1 microscope over 6 days. Dependent on the number of views, tiles, z-planes and the temporal resolution, the amount of data which is generated and needs to be processed varies between hundreds of gigabyte and tens of terabyte (cuvette 1: total acquisition time: 6 days, temporal resolution: 30 min, views: 1, tiles: 1, total size: 220 GB; cuvette 2: total acquisition time: 6 days, temporal resolution: 30 min, views: 4 (only one is shown), tiles: 1, total size: 1054 GB). Microscope: (a) Zeiss Axio Imager, (b) Zeiss Lightsheet Z.1; objective lenses: (a) Plan S 1.0x FWD 81 mm, (b) detection: W Plan-Apochromat 20x/1.0, illumination: Zeiss LSFM 10x/0.2; laser lines: 561 nm; filters: laser block filter (LBF) 405/488/561; voxel size: 1.02 × 1.02 × 2.00 μm3, recording interval: 30 min; scale bar: 100 μm. [file 12915_2021_958_MOESM7_ESM.pdf]

# Time-resolved live observation

t ----->  $\Delta t$

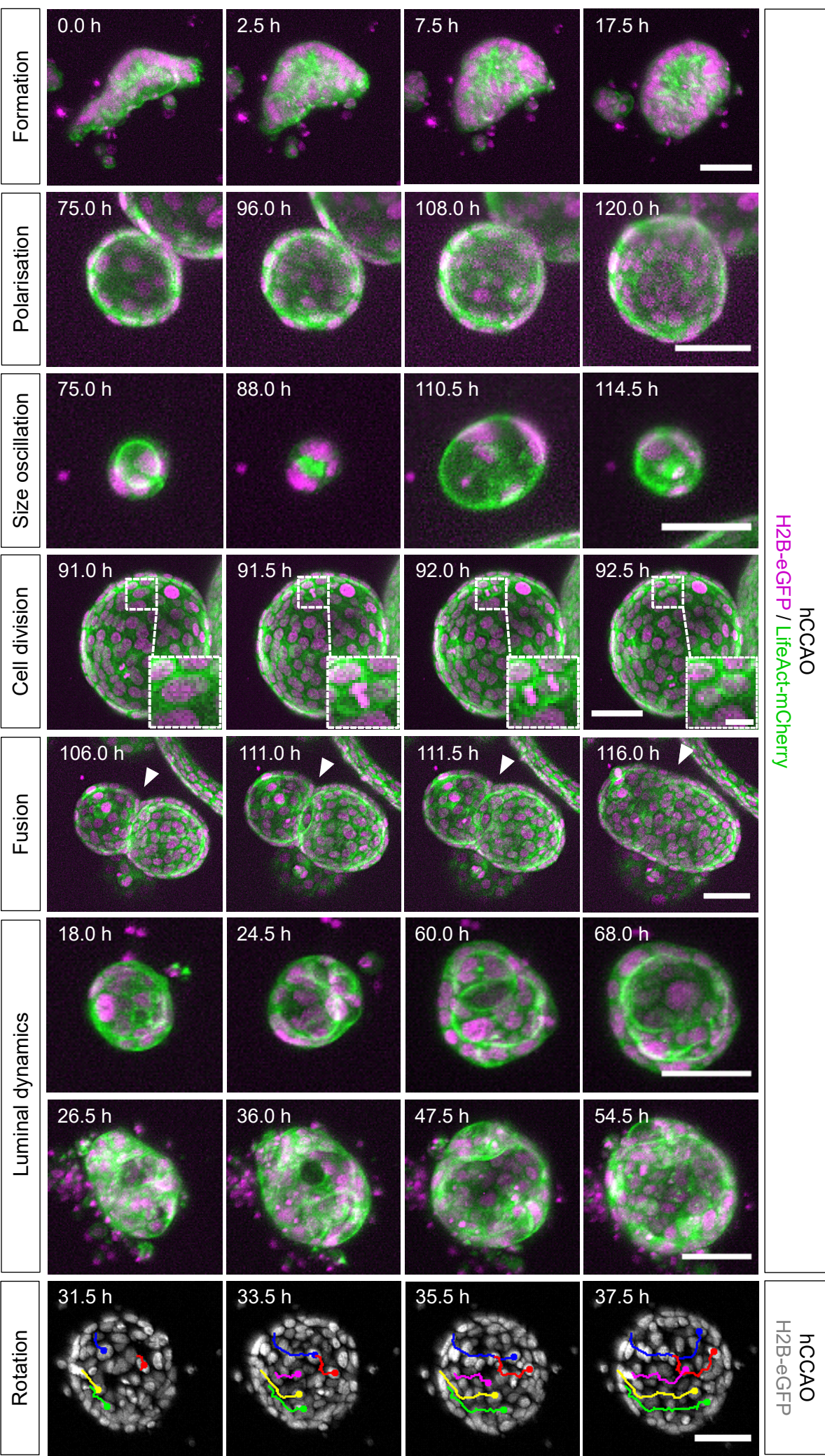

Supplement: Supplementary file 16 — Additional file 8: Fig. S7. Dynamic processes observed in organoid morphogenesis. hCCAOs expressed the nuclei marker H2B-eGFP (magenta) and the F-actin cytoskeletal marker LifeAct-mCherry (green) and were cultured in Z1-FEP-cuvettes for long-term live observation. The figure shows excerpts of maximum intensity z-projections. Manual tracking of cell nuclei was performed using the Manual Tracking plugin in Fiji. Microscope: Zeiss Lightsheet Z.1; objective lenses: detection: W Plan-Apochromat 20x/1.0, illumination: Zeiss LSFM 10x/0.2; laser lines: 488 nm, 561 nm; filters: laser block filter (LBF) 405/488/561; voxel size: 1.02 × 1.02 × 2.00 μm3; recording interval: 30 min; scale bars: 50 μm, 25 μm (inset). [file 12915_2021_958_MOESM8_ESM.pdf]

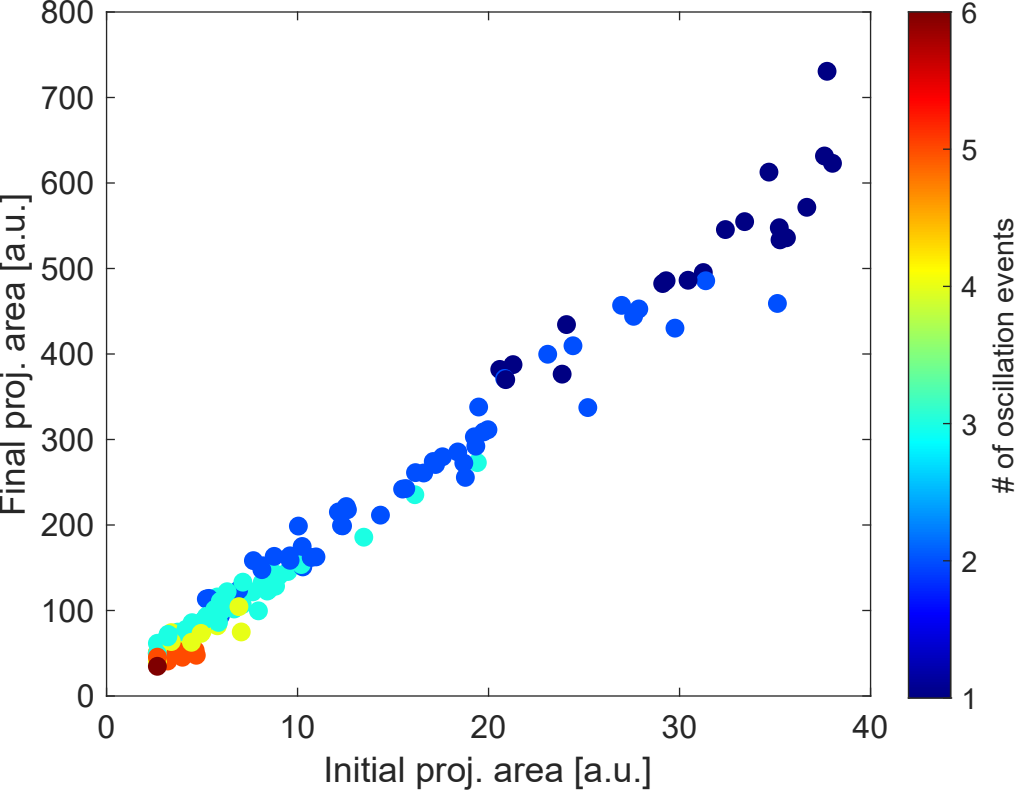

Supplement: Supplementary file 20 — Additional file 12: Fig. S11. Analysis of simulated monocystic mPOs. Simulations starting with heterogenous initial cell numbers confirm an influence of organoid size onto the number of oscillation events. The colour code signals the number of registered size oscillation events. The amount of size oscillations is dependent on the initial size of the organoids. While small organoids tend to show frequent inflation-deflation oscillations, initially larger organoids seem less prone to oscillation events. [file 12915_2021_958_MOESM12_ESM.pdf]

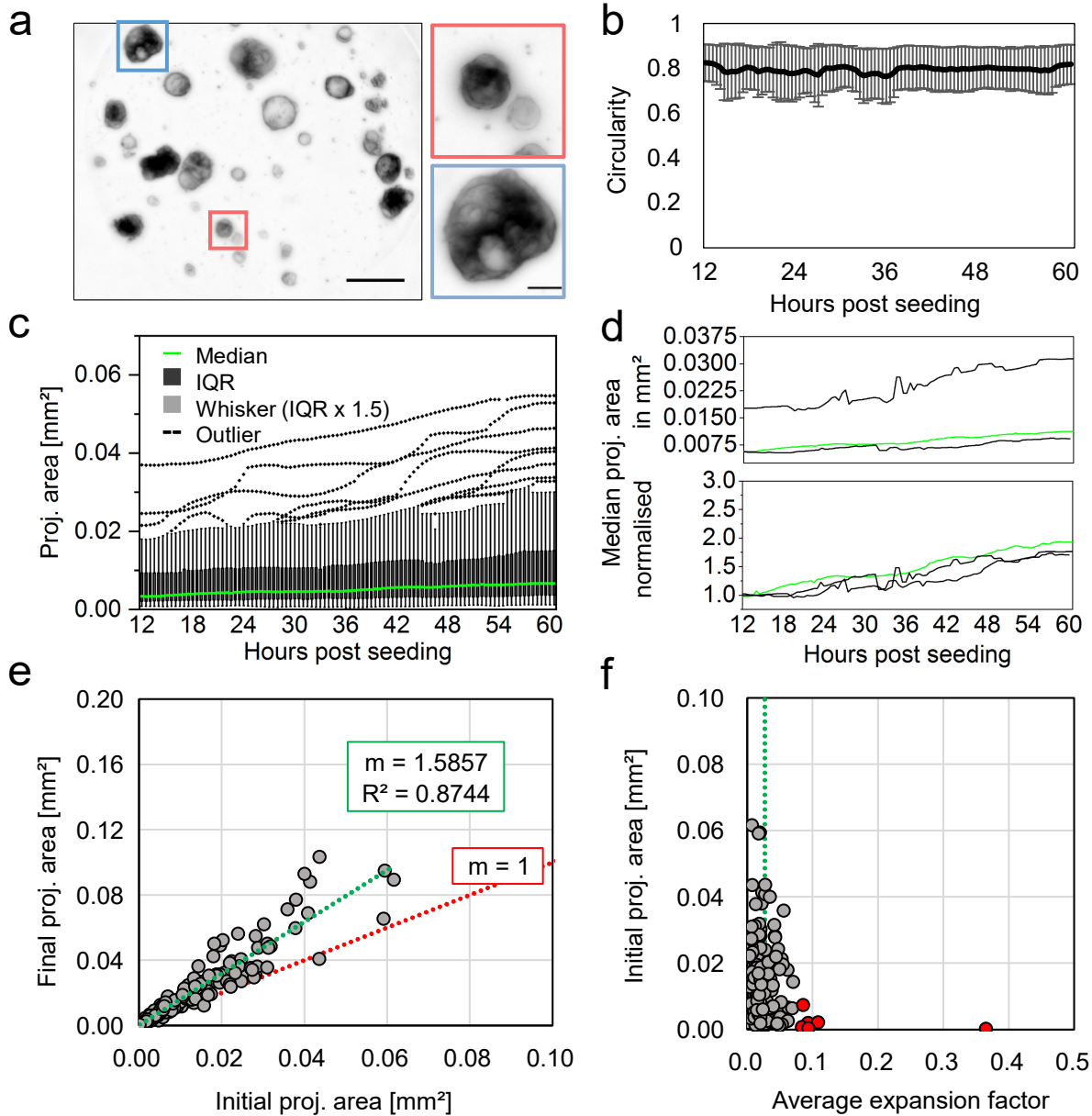

Supplement: Supplementary file 21 — Additional file 13: Fig. S12. mPO feature extraction using the bright field analysis pipeline. (a) The initial and final projected luminal areas correlate positively in healthy mPOs (R2-value = 0.7445). (b) The maximum slope of the expansion phases are in average higher than the average slope. (c) The minimum area falls in average slightly below the initial area. (d) Furthermore, the final area equals the maximum area, which indicates continuous growth – green: linear trend line, m: slope, red: f(x) = 1x. (e) Average circularity over time of organoids grown in three wells. Average standard deviation estimated within the three wells is indicated. Mathematically possible values range between 0 and 1. [file 12915_2021_958_MOESM13_ESM.pdf]

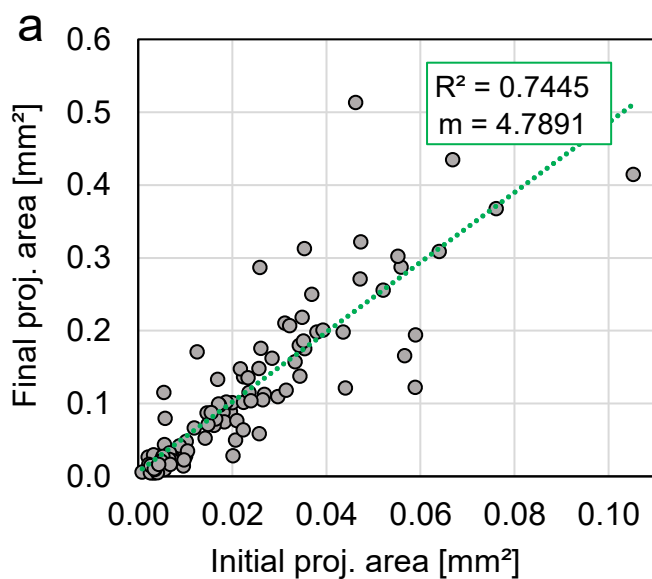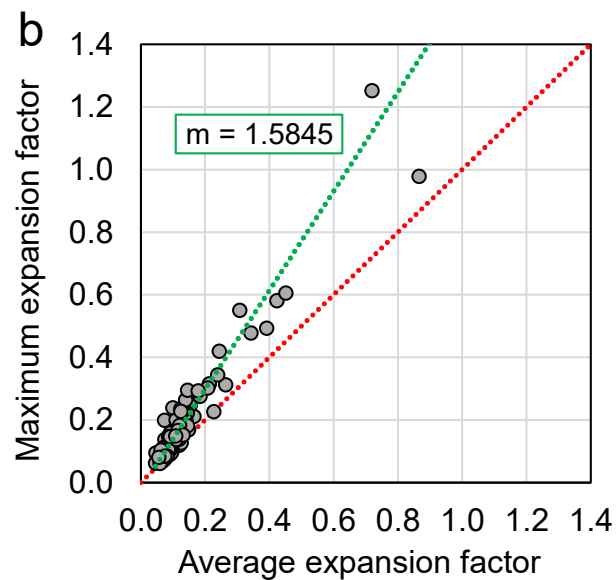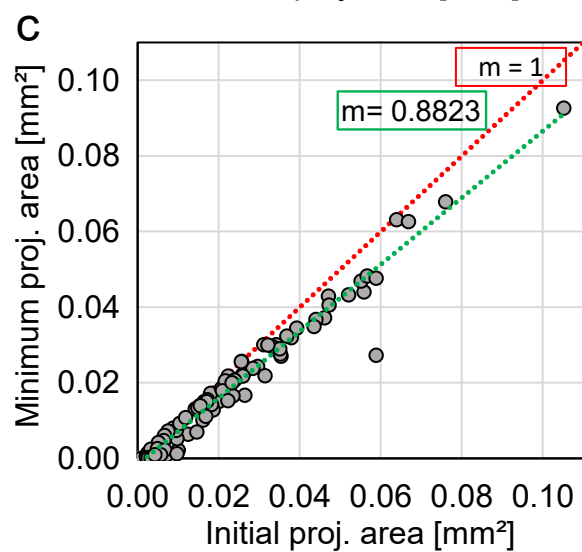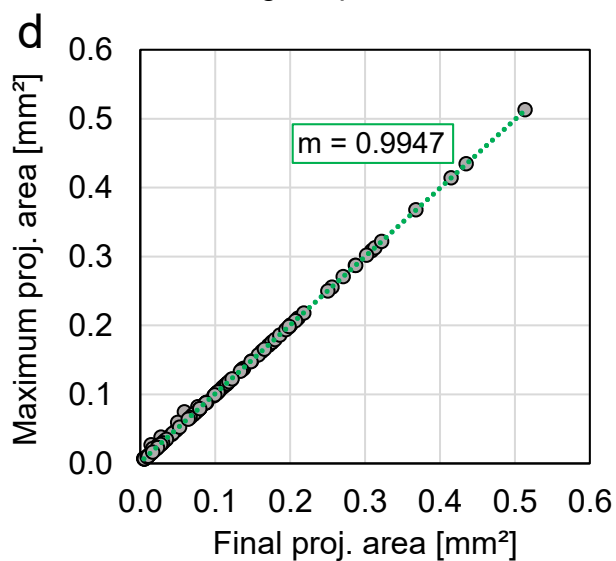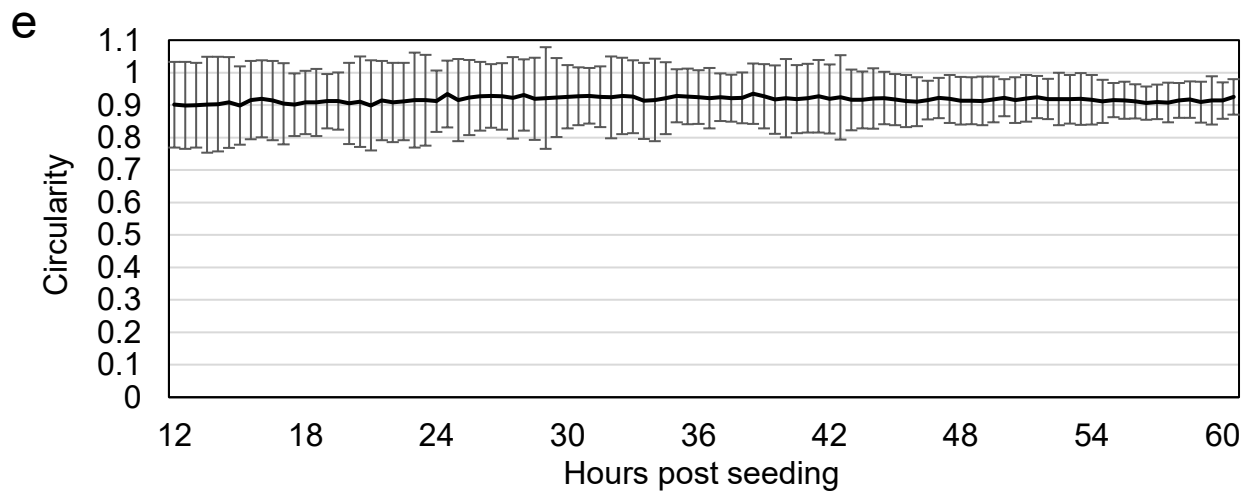

Supplement: Supplementary file 22 — Additional file 14: Fig. S13. Bright field pipeline allows detailed analysis of polycystic hCCAOs. (a) Polycystic hCCAOs display a dense phenotype. Microscope: Zeiss Axio Observer Z.1; objective lenses: Plan-Apochromat 5x/0.16, avg. z-projection, voxel size: 1.29 × 1.29 × 65 μm3, scale bar overview: 500 μm, close-up: 25 μm. (b) The average circularity is around 0.8 over time. (c) Similarly to monocystic organoid cultures, the detected projected luminal areas and growth behaviours are heterogeneous. Box plot analysis, median in green (n = 87) (d) While the median projected luminal areas of three different wells (technical replicates) vary, the normalised projected area increase is similar (n = 87, 34, 63). (e) The initial projected area correlates with the final projected area (R2 = 0.8744) with a linear regression slope m = 1.5857. (f) The organoids display a similar average expansion factor independent of their initial size with an average of 0.02 and outliers (red) lying above 0.078. [file 12915_2021_958_MOESM14_ESM.pdf]
