# Supplementary material for: Long-term live imaging and multiscale analysis identify heterogeneity and core principles of epithelial organoid morphogenesis
Source: BMC Biol. 2021 Feb 24;19:37. doi: 10.1186/s12915-021-00958-w (PMC7903752; doi:10.1186/s12915-021-00958-w)
Supplement: Supplementary file 17 — Additional file 9: Fig. S8. Time-resolved, representative images of the growth and heterogeneity in mPOs. Illustrated are single organoids extracted from three different cultures showing rotation, size oscillation, luminal dynamics, fusion and different cell nucleus sizes. In addition, cell division events at a late stage (day 4, 5, 6) and the formation of differently sized organoid fragments are shown. Besides the luminal dynamics, which are imaged by the use of a bright field microscope, all behaviour patterns and appearances were observed from organoids expressing the nuclei marker Rosa26-nTnG (grey) and grown within the Z.1-FEP-cuvette inside the Z.1 Lightsheet microscope. The red rectangles indicate the corresponding close-up, the red arrows indicate the position within the organoid where the event occurs, the curved arrow indicate the direction of rotation and the red circle indicates the volume change during a size oscillation event. Microscope fluorescence images: Zeiss Lightsheet Z.1; Plan S 1.0x FWD 81 mm, detection: W Plan-Apochromat 20x/1.0, illumination: Zeiss LSFM 10x/0.2; laser lines: 561 nm; filters: laser block filter (LBF) 405/488/561; voxel size: 1.02 × 1.02 × 2.00 μm3; recording interval: 30 min; Microscope bright field images: Zeiss Axio Observer Z.1; objective lenses: Plan-Apochromat 5x/0.16, voxel size: 1.26 × 1.26 × 4 μm3, avg. z-projection. [file 12915_2021_958_MOESM9_ESM.pdf]

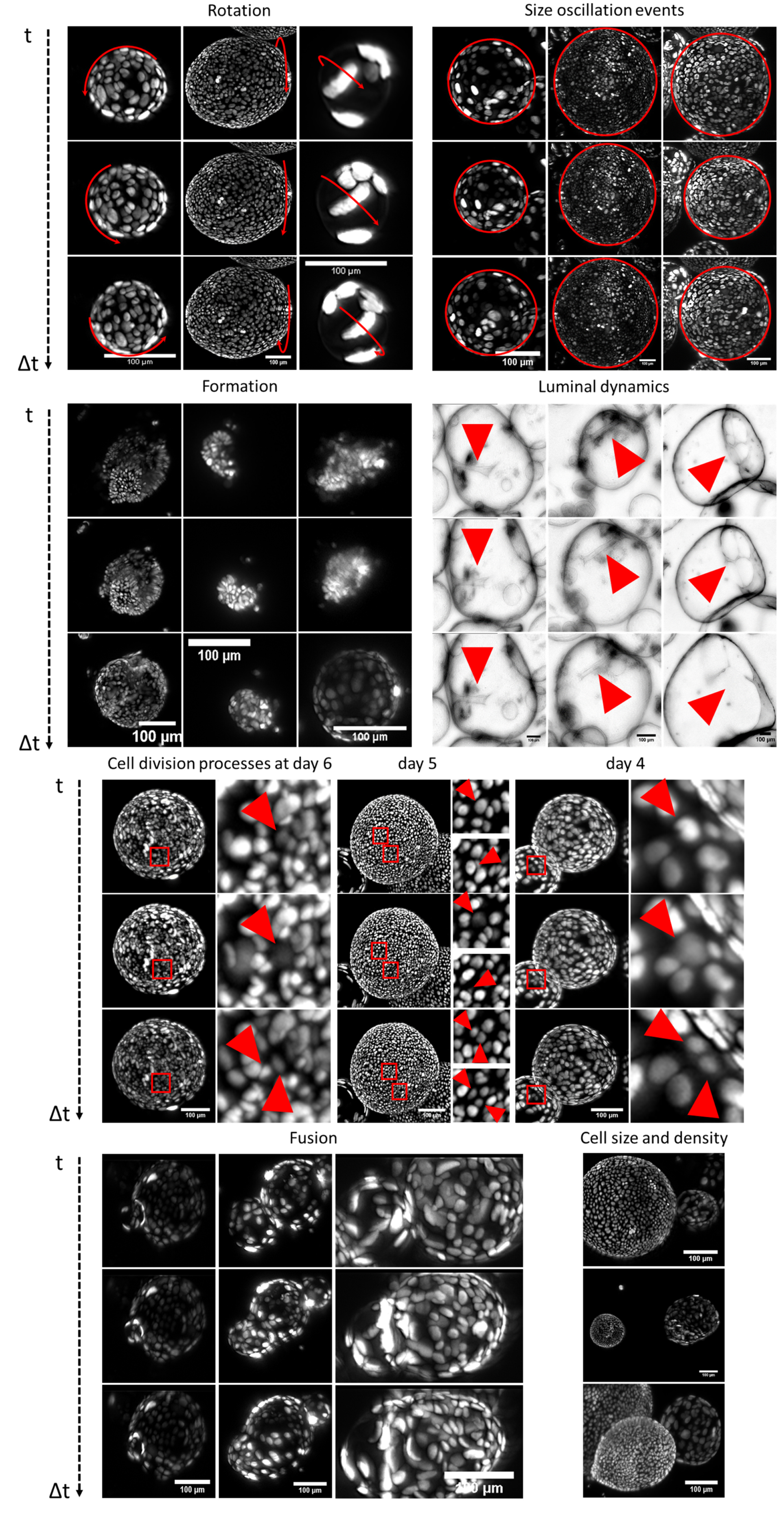

Rotation
Size oscillation events
t
Δt
100 µm
Formation
Luminal dynamics
Cell division processes at day 6
day 5
day 4
Fusion
Cell size and density
