# Supplementary material for: Long-term live imaging and multiscale analysis identify heterogeneity and core principles of epithelial organoid morphogenesis
Source: BMC Biol. 2021 Feb 24;19:37. doi: 10.1186/s12915-021-00958-w (PMC7903752; doi:10.1186/s12915-021-00958-w)
Supplement: Supplementary file 18 — Additional file 10: Fig. S9. Visualisation of segmentation performance in live mPOs expressing nuclear tdTomato. a) Maximum intensity z-projections of raw image stacks of three different mPOs. Image quality ensures a clear separation amongst the labelled nuclei, which is essential for semi-automated nuclei segmentation. Different colours indicate individual nuclei in overviews and close-ups of segmented nuclei. Microscope: Zeiss Lightsheet Z.1; objective lenses: detection: W Plan-Apochromat 20x/1.0, illumination: Zeiss LSFM 10x/0.2; laser lines: 561 nm; filters: laser block filter (LBF) 405/488/561; voxel size: 1.02 × 1.02 × 2.00 μm3; recording interval: 30 min, scale bar: 100 μm b) Evaluation of segmentation performance for different organoids. The performance was measured against a manually determined ground truth for the organoid I (red), II (blue) and III (green). The performance metrics recall, precision and F score are determined based on the number of true positives, false negatives and false positives (Supplementary Table 2). TP: true positives; FN: false negatives; FP: false negatives. [file 12915_2021_958_MOESM10_ESM.pdf]

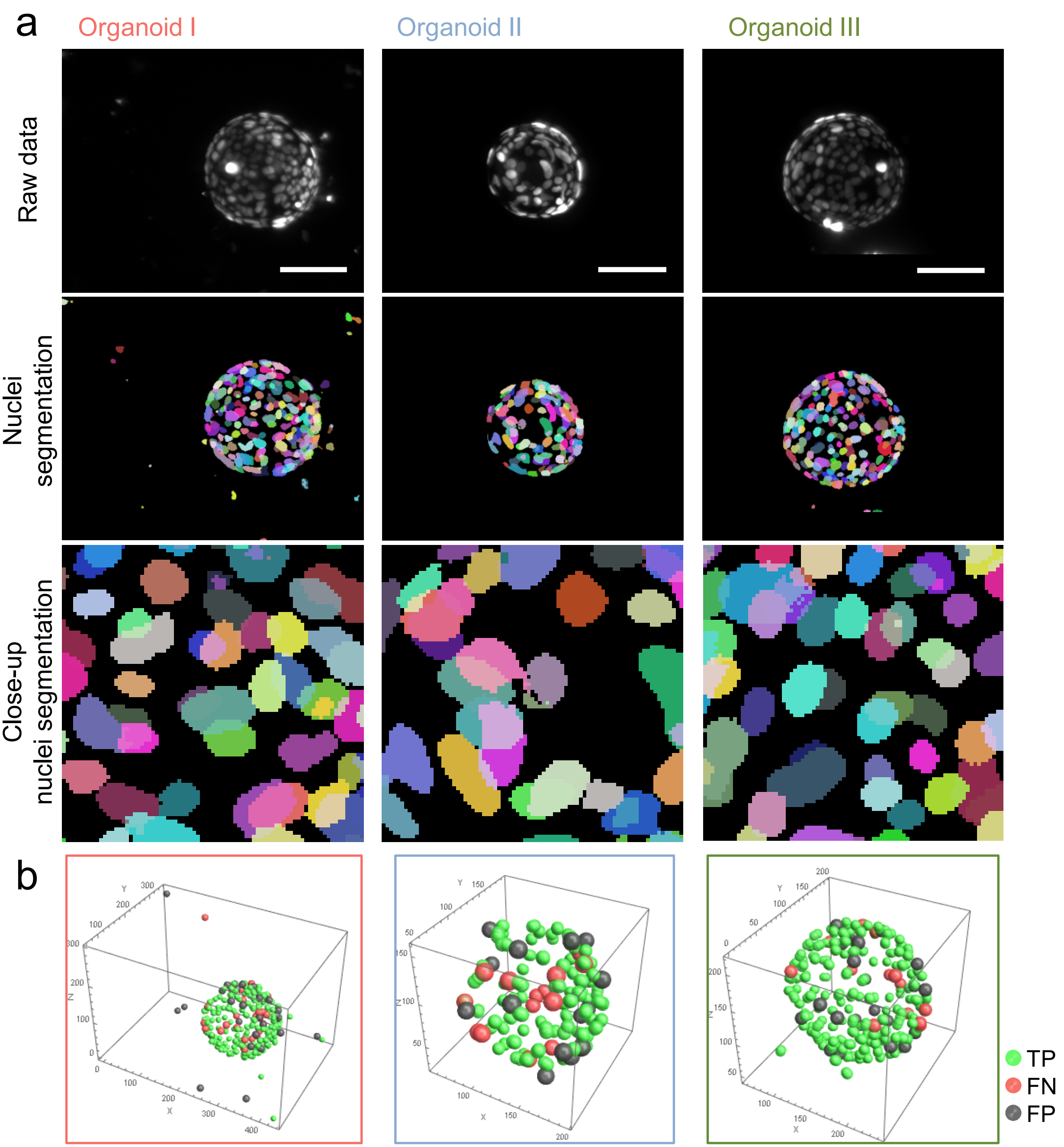

a
Organoid I
Organoid II
Organoid III
Raw data
Nuclei segmentation
Close-up nuclei segmentation
b
TP
FN
FP
