# Supplementary material for: Long-term live imaging and multiscale analysis identify heterogeneity and core principles of epithelial organoid morphogenesis
Source: BMC Biol. 2021 Feb 24;19:37. doi: 10.1186/s12915-021-00958-w (PMC7903752; doi:10.1186/s12915-021-00958-w)
Supplement: Supplementary file 19 — Additional file 11: Fig. S10. Illustration of the input, the assumptions and the output of the model. Measured cell counts and cell division dynamics are used to initialise the simulations. Organoid behaviour is based on the following main assumptions [1]. Each cell produces a substance with constant rate Jin, the substance leads to increase of internal pressure [2]. Cell displacement is driven by mechanical cell-cell-interactions, internal pressure and a surface energy of the organoid [3]. If the organoid shell ruptures, substance is released to the outside with flux Jout, releasing pressure and leading to a contraction of the sphere until the cell-cell connections are restored. The output of the model is the volume data as a function of time of the simulated organoids. [file 12915_2021_958_MOESM11_ESM.pdf]

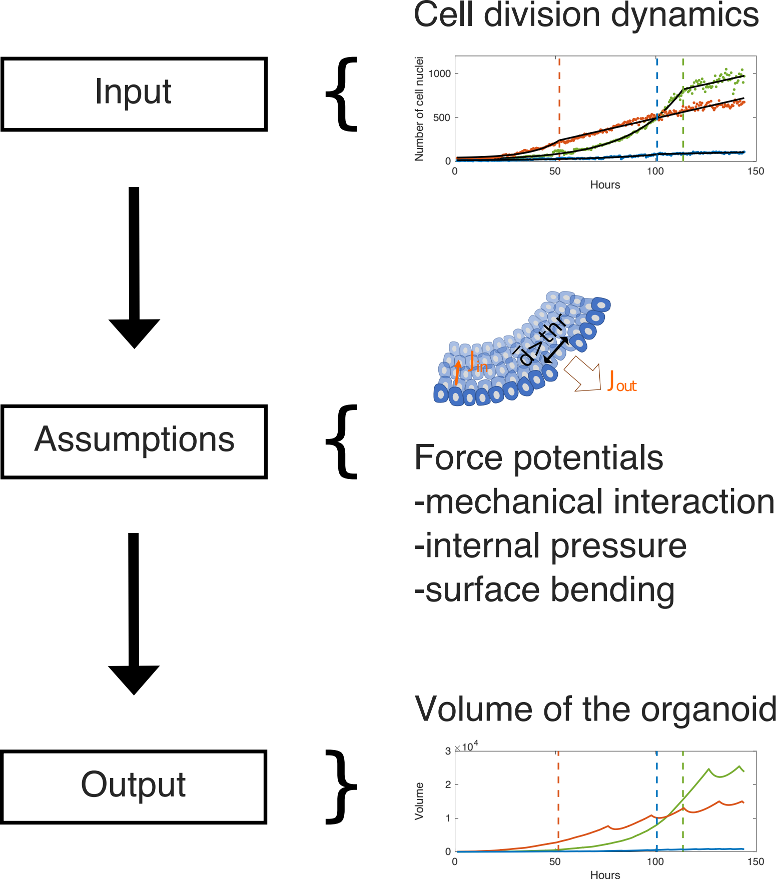
Input
Cell division dynamics
Number of cell nuclei
1000
500
0
0
50
100
150
Hours
Assumptions
J in
d>thr
J out
Force potentials
-mechanical interaction
-internal pressure
-surface bending
Output
Volume of the organoid
×10^4
3
2
1
0
Volume
0
50
100
150
Hours
