# Supplementary material for: Long-term live imaging and multiscale analysis identify heterogeneity and core principles of epithelial organoid morphogenesis
Source: BMC Biol. 2021 Feb 24;19:37. doi: 10.1186/s12915-021-00958-w (PMC7903752; doi:10.1186/s12915-021-00958-w)
Supplement: Supplementary file 23 — Additional file 23: Table S1. Used settings for the segmentation and post-processing of the data obtained with the light-sheet pipeline. Table S2. Evaluation of segmentation performance for different organoids. The performance was measured against a manually determined ground truth for organoid I (red), II (blue) and III (green). The performance metrics recall, precision and F score are determined from the number of true positives, false negatives and false positives. Values range from 0 (worst performance) to 1 (optimal performance). GT: number of cell nuclei in the ground truth; SC: number of cell nuclei determined by segmentation; TP: true positives; FN: false negatives; FP: false negatives. [file 12915_2021_958_MOESM23_ESM.pdf]

Additional file 21: Table S1-S2

Table S1: Used settings for the segmentation and post-processing of the data obtained with the light-sheet pipeline

Segmentation settings		Post processing settings	
NucleiFilterRange	2	Alpha	150
NucleiThresholdRange	10	OutlierDistanceThreshold	50
NucleiMeanFactor	1	EdgeDistanceThreshold	50
NucleiStandardDeviationFactor	0	NucleiMinCount	50
NucleiBackgroundFactor	0.9	NucleiMaxCount	10000
HoleFillingRange	1		
MaxDetectionRange	0.12		
NucleiSeedDetectionMinRadius	1		
NucleiSeedDetectionMaxRadius	4		
NucleiSeedDilation	1		

Table S2: Evaluation of segmentation performance for different organoids. The performance was measured against a manually determined ground truth for organoid I (red), II (blue) and III (green). The performance metrics recall, precision and F score are determined from the number of true positives, false negatives and false positives. Values range from 0 (worst performance) to 1 (optimal performance). GT: number of cell nuclei in the ground truth; SC: number of cell nuclei determined by segmentation; TP: true positives; FN: false negatives; FP: false negatives.

	GT	SC	TP	FN	FP	recall	precision	accuracy
organoid I	178	180	153	25	27	0.86	0.85	0.75
organoid II	93	92	79	14	14	0.85	0.85	0.74
organoid III	174	182	161	13	21	0.93	0.89	0.83
