# Supplementary material for: Long-term live imaging and multiscale analysis identify heterogeneity and core principles of epithelial organoid morphogenesis
Source: BMC Biol. 2021 Feb 24;19:37. doi: 10.1186/s12915-021-00958-w (PMC7903752; doi:10.1186/s12915-021-00958-w)
Supplement: Supplementary file 24 — Additional file 24: Supplementary Theoretical Considerations. A mechanical model to describe the dynamics of pancreas organoids. [file 12915_2021_958_MOESM24_ESM.pdf]

## Additional file 22: Supplementary theoretical considerations

## A mechanical model to describe the dynamics of pancreas organoids

To describe the growth and dynamics of a pancreas organoid, we assume it has a roughly spherical shape, with cells forming a monolayer filled with fluid at a different pressure relative to the environment. The volume of the organoid is changed by two mechanisms: a) the influx of liquid caused by an osmotic imbalance or active pumping, and b) cell division. The first mechanism increases the tension between the cells. Cell division, on the other hand, increases the surface of the organoid and reduces tension. If the stress is greater than a critical threshold, at least one cell connection breaks and leakage occur through the organoid shell. The leakage reduces the internal pressure, the monolayer can contract, which in turn allows the ruptured cells to reconnect. Subsequently, the whole process can repeat.

A triangulated-network model was used to simulate the membranes as an elastic surface consisting of cells. Here, the shell of the organoid is described as an infinitely thin elastic surface consisting of hard, spherical beads (the cell's centres) connected by dynamic bonds to form a triangulated network.

A spring potential acting on neighbouring beads is used to describe the elasticity of the shell and has the typical form

$$U_S = \frac{k_S}{2} \sum_{i,j} \left(d_{i,j} - r_0\right)^2,$$

where $k_S$ is the spring constant, $d_{i,j}$ is the distance between two neighbouring cells $i$ and $j$, and $r_0$ is their equilibrium bond length. The spring potential is minimised if $d_{i,j}$ between two neighbouring cells $i$ and $j$ corresponds to the equilibrium distance $r_0$. Since the method of finite elements is used in the simulation, the force must be derived from the potential used. The force acting on every cell is given as

$$F_S = -k_S \sum_{k,i\neq k} \left(d_{i,j} - r_0\right) e_{i,k},$$

where $k$ are the indices of the neighbours of the cell $i$, and $e_{i,j}$ is the normalised direction vector between $\boldsymbol{x}_i$ and its neighbour $\boldsymbol{x}_j$.

The surface bending energy acts on neighbouring triangles and is minimised when the angle between the neighbouring triangles is zero.

$$U_B = k_B(1 - \boldsymbol{n} \cdot \boldsymbol{m}),$$

where $\boldsymbol{n}$ and $\boldsymbol{m}$ are the normal vectors of two neighbouring triangles sharing a common edge b. The resulting force can be generated by deriving the bending potential after point $\boldsymbol{x}_i$.

$$F_B = k_B \frac{\boldsymbol{b} \times \boldsymbol{m}}{|\boldsymbol{n}|} \left( \underline{\hat{1}} - \left[ \frac{\boldsymbol{b} \times \boldsymbol{m}}{|\boldsymbol{n}|^2} \right] \otimes \boldsymbol{a} \right),$$

where $\boldsymbol{a}$ describes the direction vector from $\boldsymbol{x_i}$ to $\boldsymbol{x_j}$ in the triangle $\boldsymbol{x_i x_j x_k}$ and $\boldsymbol{a} \otimes \boldsymbol{b}$ denotes the dynamic product of the two vectors $\boldsymbol{a}$ and $\boldsymbol{b}$. For one cell $\boldsymbol{x_i}$ it is then summed over all $\boldsymbol{n}, \boldsymbol{m}$ pairs of the neighbouring triangles of $\boldsymbol{x_i}$. In order to compensate for the differences between cells with different numbers of neighbouring cells, a normalisation is made about the number of neighbouring cells.

In the simulation, the assumption is made that each cell pumps mass (e.g. water) or fluid through an osmotic imbalance into the lumen of the organoid and thus, an internal pressure that differs from the external pressure can build up. The internal pressure is one of the factors of organoid expansion. The force $F_P$, which affects each mass point from the resulting osmotic pressure $\Pi$, is given as

$$F_P = k_P P A \boldsymbol{n_v},$$

whereby

$$A = \frac{1}{3}\sum_{i=1}^{Nb} \frac{1}{2}\boldsymbol{n_i}\,.$$

The osmotic pressure $\Pi$ has an effect on the area $A$, which is understood as the sum of the adjacent triangular areas to the cell center $\boldsymbol{x_i}$, with the direction vector $\boldsymbol{n}$ (normal vector to cell $i$= summed normal vectors of the adjacent triangles). The osmotic pressure $\Pi$ is calculated using the van-'t-Hoff law for osmotic pressure

$$\Pi = \frac{n}{V} \cdot i_{vH} \cdot R \cdot T = \frac{n}{V} \cdot const,$$

where $n$ is the amount of substance, $i_{vH}$ is the van-'t-Hoff factor, $R$ is the ideal gas constant and $T$ is the temperature. The volume of the organoid $V$ is calculated for each time step over the convex shell of the cells. The amount of secreted substance changes over time with

$$\frac{dn}{dt} = NJ_{in} - J_{out}n.$$

$N$ indicates the number of cells in the organoid, $J_{in}$ the amount of substance produced per cell, and $J_{out}$ describes the substance drop through a hole in the organoid shell. If the organoid shell has a rupture, $J_{out}$ is greater than zero, otherwise $J_{out}$ is zero. The equation of motion used in the simulation applies to the overdamped case and contains stochastic fluctuations $F_t$ of the cells,

$$\lambda \boldsymbol{x} = -\nabla U + F_t,$$

whereby the potential $U$ is given as the sum of the above-mentioned potentials.

Cell division is adjusted to the experimental data, obtained by long-term single cell analysis of pancreas-derived organoids, but can easily be adapted to other growth dynamics. If cell division takes place a new cell is added to the system in the middle of a random triangle formed by three neighbouring cells.
